# Mitochondrial diseases: from molecular mechanisms to therapeutic advances

**DOI:** 10.1038/s41392-024-02044-3

**Published:** 2025-01-10

**Authors:** Haipeng Wen, Hui Deng, Bingyan Li, Junyu Chen, Junye Zhu, Xian Zhang, Shigeo Yoshida, Yedi Zhou

**Affiliations:** 1 Department of Ophthalmology, The Second Xiangya Hospital of Central South University, Changsha, Hunan 410011 China; 2 Xiangya School of Medicine, Central South University, Changsha, Hunan 410013 China; 3 Hunan Clinical Research Center of Ophthalmic Disease, Changsha, Hunan 410011 China; 4 Department of Ophthalmology, Kurume University School of Medicine, Kurume, Fukuoka, 830-0011 Japan

**Keywords:** Diseases, Medical genetics, Molecular medicine

## Abstract

Mitochondria are essential for cellular function and viability, serving as central hubs of metabolism and signaling. They possess various metabolic and quality control mechanisms crucial for maintaining normal cellular activities. Mitochondrial genetic disorders can arise from a wide range of mutations in either mitochondrial or nuclear DNA, which encode mitochondrial proteins or other contents. These genetic defects can lead to a breakdown of mitochondrial function and metabolism, such as the collapse of oxidative phosphorylation, one of the mitochondria’s most critical functions. Mitochondrial diseases, a common group of genetic disorders, are characterized by significant phenotypic and genetic heterogeneity. Clinical symptoms can manifest in various systems and organs throughout the body, with differing degrees and forms of severity. The complexity of the relationship between mitochondria and mitochondrial diseases results in an inadequate understanding of the genotype-phenotype correlation of these diseases, historically making diagnosis and treatment challenging and often leading to unsatisfactory clinical outcomes. However, recent advancements in research and technology have significantly improved our understanding and management of these conditions. Clinical translations of mitochondria-related therapies are actively progressing. This review focuses on the physiological mechanisms of mitochondria, the pathogenesis of mitochondrial diseases, and potential diagnostic and therapeutic applications. Additionally, this review discusses future perspectives on mitochondrial genetic diseases.

## Introduction

Mitochondria, often referred to as the powerhouses of cells, perform their essential function through oxidative phosphorylation (OXPHOS), which generates ATP as a vital energy source.^1^ Mitochondrial diseases are genetic disorders resulting from abnormalities of mitochondrial function.^2^ These disorders arise from mutations in either mitochondrial DNA (mtDNA) or nuclear DNA (nDNA), both of which encode subunits of OXPHOS as well as structural or functional mitochondrial proteins.^3^ These proteins are not only integral to classical mitochondrial metabolism—such as OXPHOS, the Krebs cycle, lipid metabolism, and nucleotide metabolism—but also play key roles in mitochondrial quality control, calcium homeostasis, cell death, and inflammation. Deficiencies in these proteins can lead to mitochondrial dysfunction and subsequent energy failure.^4^ Given mitochondria’s ubiquitous presence and critical role in cellular metabolism, any tissue in the body can be affected.^5^ However, organs and tissues with high energy demands, such as the brain, nerve, eye, cardiac, and skeletal muscles, are particularly susceptible to energy failure due to OXPHOS defects, with phenotypes often manifesting in neurological, ophthalmological, and cardiological systems.^6^ The symptoms of mitochondrial diseases are diverse, with developmental delay, seizure (encephalopathy), hypotonia (myopathy), and visual impairment (retinopathy) being prominent indicators.^6,7^ Despite recent advances, the molecular mechanisms underlying these diseases remain incompletely understood. The extreme phenotypic and genetic heterogeneity of mitochondrial diseases further complicates diagnosis, making misdiagnosis a common issue.^8^

Mitochondrial diseases have been recognized as pathway-based diseases rather than merely energy-deficit diseases.^7^ The variable clinical presentations and tissue specificity suggest that there are contributing factors beyond energy deficit during disease development.^9^ The reduction of ATP produced from OXPHOS can be compensated by enhanced anaerobic glycolysis, and thus mitochondrial genetic defects may not reduce ATP production.^10,11^ Furthermore, genetic defects are not always sufficient to cause cellular dysfunction as mitochondria can buffer against mitochondrial lesions, making environmental insults sometimes important to trigger these genetic disorders.^12^ Recently, the mitochondrial stress responses have gained close attention.^9^ Mitochondria have a comprehensive quality control system to maintain homeostasis, preventing dysfunction when facing stress. At the molecular level, mitochondria possess the quality control mechanisms of the proteome, such as mitochondrial integrated stress response (mt-ISR).^13^ At the organelle level, mitochondria can alter their morphology or sub-location through fusion, fission, and transport to adapt to stress or damage. At the cellular level, mitophagy coordinates with mitochondrial biogenesis, controlling the health of the mitochondrial population.^14,15^ Intercellular mitochondria transfer also plays a role in maintaining mitochondrial homeostasis.^16^ However, excessive stress can trigger mitochondria-related inflammation or apoptosis as well.^17^ In the context of mitochondrial diseases, genetic defects can lead to mitochondrial dysfunction. The subsequent responses to the stress induced by mitochondrial dysfunction may aid in understanding these genetic diseases.^9,18^ Hence, this review concludes the physiological processes of mitochondria and the potential pathogenesis of mitochondrial diseases. Significant progress in diagnosis and treatment is also summarized in this review.

## Historical review and Epidemiology of mitochondrial diseases

The history of mitochondrial diseases dates back to 1871 when *Theodor* *Leber* documented hereditary and congenital optic nerve diseases, marking the first known description of a genetic mitochondrial disorder, now recognized as Leber hereditary optic neuropathy (LHON).^19^ The concept of mitochondrial diseases was later introduced in 1962 by Luft et al.^20^, who identified a young woman with severe hypermetabolism caused by mitochondrial dysfunction due to defective OXPHOS coupling in skeletal muscle mitochondria.^20,21^ This pivotal discovery brought mitochondrial diseases into the scientific spotlight.

During the 1960s, research primarily focused on mitochondrial myopathies. *Milton Shy* and *Nicholas Gonatas* described megaconial and pleoconial congenital myopathies,^22,23^ hypothesizing that these conditions were linked to mtDNA defects.^24^ In 1963, *Engel* et al. introduced an improved Gomori trichrome staining method for muscle histopathology, which enabled the detection of abnormal mitochondrial proliferation as ragged-red fibers, thus advancing histochemical studies of mitochondrial diseases.^25^ The 1970s saw significant progress in identifying mitochondrial metabolism defects through histochemical assays, including deficiencies in pyruvate dehydrogenase, carnitine, cytochrome c oxidase, and carnitine palmitoyltransferase.^26–29^ In 1977, *Shapira* et al. coined the term “mitochondrial encephalomyopathies” to describe a group of neuromuscular disorders characterized by defects in oxidative metabolism.^30^ A major breakthrough came in 1981 when *Anderson* et al. successfully mapped the entire mitochondrial genome, establishing a foundation for subsequent mitochondrial research.^31^

In 1988, the discovery of single large-scale deletions of up to 7 kilobases in patients with mitochondrial myopathies^32^ and a point mutation in the NADH dehydrogenase subunit 4 gene in families with LHON^33^ underscored the importance of mtDNA mutations, heralding the beginning of the molecular era in mitochondrial research.^34^ By 1989, multiple mtDNA deletions had been identified in the muscle tissues of members from a family with autosomal dominant mitochondrial myopathy.^35^ Further advancements were made in 1991 when *Moraes* et al. confirmed mtDNA depletion in the affected muscle or liver tissues of infants with autosomal recessive disorders.^36^ This period also saw increased attention to the role of nDNA in mitochondrial diseases, particularly with the identification of Mendelian mitochondrial disorders. A landmark discovery in 1995 revealed the first nuclear gene mutation causing mitochondrial respiratory chain deficiency in humans: a mutation in the nuclear-encoded flavoprotein subunit gene of succinate dehydrogenase led to complex II deficiency in two sisters with Leigh syndrome.^37^ The creation of the first comprehensive mtDNA database, MITOMAP, in 1996 further facilitated the study of mitochondrial diseases.^38^ Soon after, *Nishino* et al. attributed mitochondrial neurogastrointestinal encephalomyopathy (MNGIE) to a defect in communication between nuclear and mitochondrial genomes.^39^ The 2000s saw the introduction of next-generation sequencing (NGS) technology in the diagnosis of mitochondrial diseases.^40^ By the 2010s, transcriptomics and other omics analyses had gained increasing attention, leading to the emergence of multi-omics approaches in the diagnosis of mitochondrial disorders.^41^

Scientists are actively pursuing potential treatments to address mitochondrial diseases. In 1997, *Taylor* et al. pioneered the use of peptide nucleic acid (PNA) in gene therapy to selectively inhibit the replication of mutated human mtDNA, thereby increasing the proportion of wild-type mtDNA and correcting defective phenotypes through heteroplasmy alteration.^42^ In 2006, *Spees* et al. discovered that intercellular mitochondrial transfer could restore aerobic respiration in mammalian cells.^43^ By 2009, *Tachibana* et al. had successfully separated the spindle-chromosome complex from mature metaphase II (MII) oocytes and transferred it into enucleated oocytes, resulting in the birth of healthy primate offspring with nDNA from the spindle donor and mtDNA from the cytoplasmic donor.^44^ In 2015, idebenone received approval from the European Medicine Agency (EMA) for treating LHON under specific conditions.^45^ In 2017, *Zhang* et al. reported the application of the spindle-chromosome complex transfer (ST) method in a woman carrying the m.8993 T > G mutation associated with Leigh syndrome, leading to the birth of a healthy child.^46^ In 2018, a gene therapy employing an allogeneic expression strategy was tested in a clinical trial for patients with LHON, demonstrating both safety and good tolerability.^47^ More recently, in 2023, Omaveloxolone became the first drug approved by the Food and Drug Administration (FDA) for treating Friedreich’s ataxia.^48^

**Fig. 1:**
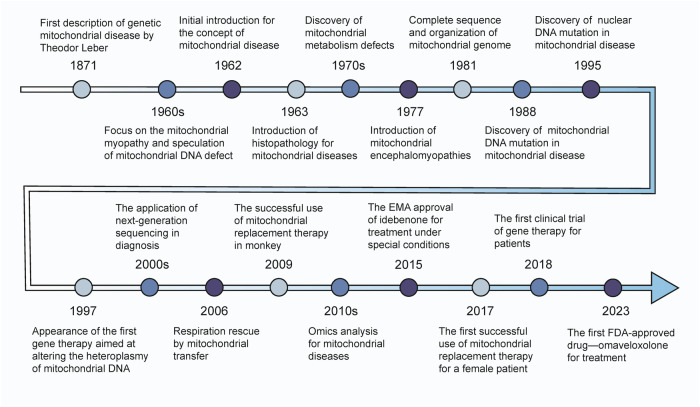
Timeline of Major Historical Events in the Study of Mitochondrial Diseases. From the initial discovery to current advancements, our understanding of the mechanisms underlying mitochondrial diseases has continually deepened. Over time, research explorations and progress have contributed to the development of diagnostic and treatment methods, ultimately providing insights into more efficient and accurate diagnostic and therapeutic strategies

As our understanding of mitochondria deepens, so does our knowledge of the mutant genes and pathogenesis underlying mitochondrial genetic disorders. Figure 1 presents a timeline summarizing key milestones in mitochondrial disease research. Beyond the focus on primary or secondary OXPHOS, significant attention is being directed toward gene mutations that impair mitochondrial structure and function.^2,3,41^

Previous studies estimate the global prevalence of mitochondrial diseases at approximately 1 in 5,000 births,^49^ with pathogenic mtDNA mutations affecting at least 12.48 per 100,000 individuals.^50^ Table 1 lists the regional and global incidences of specific mitochondrial diseases.

**Table 1:** The prevalence of overall or individual mitochondrial diseases

Category	Prevalence (95% CI)	Population	Region	Reference
Mitochondrial diseases (caused by mtDNA mutation)	12.48(10.75–14.23)/100,000	Female (age<60) Male (age<65)	Northeast England	^50^
	9.2 (6.5–12.7)/100,000	Age≥18	Southwest Finland	^881^
Mitochondrial diseases	12.5 (11.1–14.1)/100,000	Female (16<age<60) Male (16<age<65)	Northeast England	^882^
	4.7 (4.1–5.4)/100,000	Total	New Zealand	^883^
	1.02 (0.81–1.28)/100,000	Total	Hong Kong, China	^884^
	2.9 (2.8–3.0)/100,000	Total	Japan	^885^
	2.3 (2.14–2.47)/1,000,000	Total	Spain	^886^
	7.5 (5.0–10.0)/100,000	Age≤18	Northwest Spain	^887^
LHON	2491 (1996–2986)/126,167,000	Total	Japan	^888^
	3.22 (2.47–3.97)/100,000	Age<65	Northeast England	^889^
	2.06 (1.8–2.4)/100,000	Age≥5	Finland	^890^
	1/54,000	Total	Denmark	^891^
	1/68,403	Age<85	Australia	^892^
	1/39,000	Total	Netherlands	^893^
	1.9/1,000,000	Total	Serbia	^894^
MELAS	0.18 (0.02–0.34)/100,000	Total	Japan	^895^
	4.7 (2.8–7.6)/100,000	Age<16	Western Sweden	^896^
Mitochondrial myopathy	0.58 (0.54–0.62)/100,000	Total	Japan	^895^
Leigh syndrome	2.05 (0.72–3.40)/100,000	Age≤18	Northwest Spain	^887^
Friedreich’s ataxia	1/176,000	Total	Norway	^897^
	1.2 (0.9–1.6)/100,000	Total	Italy	^898^
ADOA	2.87 (2.54–3.20)/100,000	Total	North England	^899^
	1/12,301	Total	Denmark	^900^
MNGIE	1–9/1,000,000	Total	World	^488^
MIDD	0.5–2.8/100	Diabetic patients	World	^901^
Barth syndrome	1/1,000,000	Male	World	^902^

*LHON* Leber hereditary optic neuropathy, *MELAS* mitochondrial myopathy, encephalopathy, lactic acidosis and stroke-like episodes, *ADOA* autosomal dominant optic atrophy, *MNGIE* mitochondrial neurogastrointestinal encephalomyopathy, *MIDD* maternally inherited diabetes and deafness

Determining the exact global incidence of mitochondrial diseases is challenging due to their rarity, high mortality, and clinical and genetic heterogeneity.^51^ Additionally, symptoms typically manifest only when a certain mutation threshold is reached—usually 80–90%—though this threshold can vary between different cells and patients.^52,53^ As a result, the clinical phenotypes of mitochondrial diseases caused by mtDNA mutations can differ significantly among individuals and are influenced by the level of heteroplasmy, making these diseases difficult to diagnose.^54^ Notably, mtDNA mutations are not exclusive to those with mitochondrial diseases; they are present in the general population as well. At least 1 in 200 healthy individuals carries a pathogenic mtDNA mutation, often with no or only mild symptoms.^55^ These mutations can be maternally inherited, and it is estimated that nearly 2473 women in the UK and 12,423 women in the US, aged 15 to 44, carry pathogenic mtDNA mutations.^56^ Interestingly, approximately 80% of mitochondrial diseases in adults are linked to mtDNA mutations, while most mitochondrial diseases in children are associated with nDNA mutations, with only 20–25% stemming from mtDNA mutations.^2^ These factors underscore the complexity and prevalence of mitochondrial diseases, which are more common and intricate than previously understood. Consequently, further epidemiological studies are essential to improve our understanding and prediction of mitochondrial disease prevalence.

## Molecular basis of mitochondria

### General characteristics of mitochondria

The mitochondrion is a double-membrane organelle present in nearly all eukaryotic organisms.^57^ It is widely believed that mitochondria originated from bacteria, specifically α-proteobacteria.^58^ The human mitochondrion contains a genome of 16,569 base pairs, distinct from the nuclear genome.^31^ Notably, fragments of mtDNA can integrate into the nuclear genome, forming nuclear-mitochondrial segments (NUMTs).^59^ The mtDNA is a circular, double-stranded molecule with multiple copies and is maternally inherited. It encodes 37 genes, including 2 rRNAs, 22 tRNAs, and 11 mRNAs. Of these, 14 tRNAs, 2 rRNAs, and 10 mRNAs are encoded on the heavy (H) strand, while the remaining 1 mRNA and 8 tRNAs are encoded on the light (L) strand.^60–62^

Mitochondrial non-coding RNAs (ncRNAs), such as microRNAs, long non-coding RNAs, circular RNAs, and piwi-interacting RNAs, have been identified as potential mediators of mitochondrial homeostasis.^63^ These ncRNAs are key messengers in mito-nuclear communication and have garnered significant attention.^64^ Most mitochondrial ncRNAs originate from the nuclear genome and are translocated into mitochondria *via* Ago2, PNPase, or associated mitochondrial proteins. These nuclear-derived ncRNAs can indirectly regulate mitochondrial homeostasis by influencing nDNA-encoded mitochondrial proteins.^63^ Conversely, mitochondria-derived ncRNAs, which include a limited number of long non-coding RNAs and circular RNAs, can directly regulate mtDNA expression or mitochondrial protein transport.^65–67^ The biogenesis, processing, and functional mechanisms of ncRNAs encoded by mtDNA remain largely unclear.^63^ Intriguingly, recent studies suggest the epigenetic inheritance-influenced transfer of mitochondrial tRNA (mt-tRNA) from sperm to oocyte at fertilization, highlighting the potential importance of paternal factors in mitochondrial inheritance.^68^ Additionally, mt-tRNA fragments have been implicated in mitochondrial diseases.^69^

Mitochondrial proteomes, comprising approximately 1000 to 1500 proteins, are encoded by both nDNA and mtDNA.^70,71^ Among these, metabolism-related proteins constitute the largest number and abundance.^72^ While mtDNA encodes only 13 proteins involved in OXPHOS, the vast majority of mitochondrial proteins (>99%) are encoded by the nuclear genome, synthesized on ribosomes, and subsequently imported into mitochondria.^73^

Mitochondrial-derived peptides (MDPs) are encoded by short open reading frames within mtDNA, including humanin, small humanin-like peptides 1-6 (SHLP1-6), MOTS-c, and CYTB-187AA.^74–76^ These MDPs play a pivotal role in cellular protection by maintaining homeostasis and cellular function.^75^ Each peptide exhibits distinct biological effects; for instance, humanin, SHLP2, and SHLP3 inhibit apoptosis and promote cell viability, whereas SHLP6 induces apoptosis.^75,77^ SHLP2 and SHLP4 also enhance cell proliferation.^75^ Humanin is essential for maintaining mitochondrial homeostasis and function by increasing mtDNA copy number and mitochondrial mass and promoting mitochondrial biogenesis.^77^ Similarly, SHLP2 and SHLP3 contribute to mitochondrial biogenesis, enhancing mitochondrial metabolism and function.^74,75,78^ MOTS-c, the first MDP discovered to enter the cell nucleus, plays a role in mito-nuclear communication.^74^ MOTS-c transcripts originate in mitochondria, are exported to the cytosol for translation into peptides, and then return to mitochondria.^74^ Under stress, AMPK activation triggers the translocation of MOTS-c to the nucleus, where it binds to nuclear DNA and interacts with transcription factors such as NRF2 and ATF1, modulating nuclear gene expression to restore cellular metabolic homeostasis.^74,79^ Recently, *Hu* et al. demonstrated that the cytochrome b transcript, encoded by mtDNA, is translated by cytosolic ribosomes using the standard genetic code to produce a 187-amino acid protein, CYTB-187AA.^76^ CYTB-187AA localizes to the mitochondrial matrix and interacts with SLC25A3 to regulate ATP production.^76^ Single nucleotide polymorphisms in the mtDNA coding regions for MDPs may facilitate the discovery of new MDPs, such as SHMOOSE.^80^

Without the protective presence of histones, mtDNA is more susceptible to external factors, leading to a higher likelihood of mutations. These mutations can result in various diseases, given the critical role of mitochondria in nucleated cells. The severity of such conditions often depends on the ratio of mutant to wild-type mtDNA.^81^ Because mtDNA exists in multiple copies, two scenarios are possible: homoplasmy, where all mtDNA copies are identical, or heteroplasmy, where the copies differ.^82^

Mitochondrial membrane potential (Δψm) is a fundamental property generated during OXPHOS by the respiratory chain. The stability of Δψm is essential for cell viability. Under normal conditions, Δψm may fluctuate slightly in the short term; however, prolonged changes in Δψm can lead to pathological outcomes. As a result, cells activate mechanisms to eliminate mitochondria with abnormal Δψm.^83,84^

### Mitochondrial metabolism

**Fig. 2:**
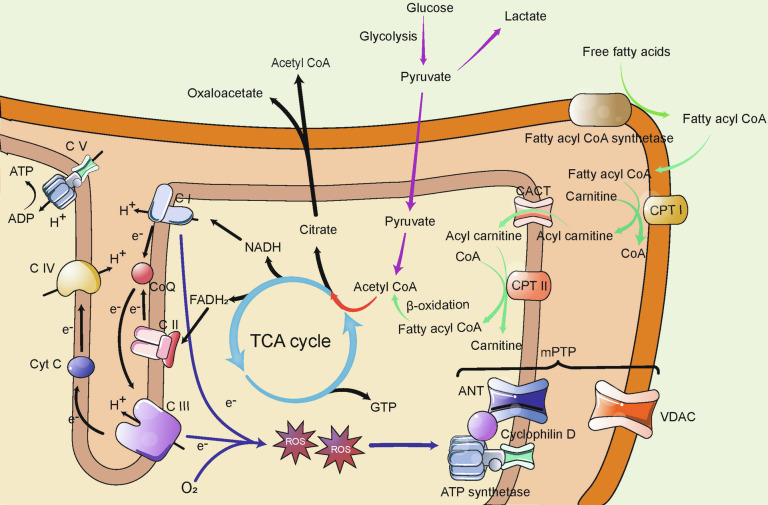
Overview of Mitochondrial Metabolism. As the central hub of bioenergetics, the mitochondrion utilizes NADH and FADH_2_ produced by the TCA cycle to generate ATP through electron transfer and the H^+^ gradient across the respiratory chain complexes. Complexes I and III are the primary sources of mtROS, which cause oxidative damage or signaling transduction. The mtROS also can induce the opening of mPTP. Glucose and lipids (*via* β-oxidation) both contribute to the TCA cycle. Citrate can cross the mitochondrial membrane, allowing acetyl-CoA to be transported into the cytoplasm for various functions. TCA cycle tricarboxylic acid cycle; CACT carnitine-acylcarnitine translocase; CPT I/II carnitine palmitoyltransferase I/II; mtROS mitochondrial reactive oxygen species; ANT adenine nucleotide translocator; VDAC voltage-dependent anion channel; mPTP mitochondrial permeability transition pore

Mitochondria play a central role in substance metabolism, overseeing a vast array of metabolic processes as depicted in Fig. 2.

#### Oxidative phosphorylation

The primary function of mitochondria is energy production, with the majority of ATP being generated through OXPHOS.^85^ The OXPHOS system, essential for mitochondrial respiration, consists of five multimeric protein complexes located in the cristae of the inner mitochondrial membrane (IMM).^86^ The respiratory chain complexes (Complexes I–IV), collectively known as the electron transport chain (ETC), facilitate the transfer of electrons from nicotinamide adenine dinucleotide (NADH) and flavin adenine dinucleotide (FADH_2_) to oxygen through a series of redox reactions. This process contributes to the formation of an electrochemical (proton) gradient across the IMM, ultimately reducing oxygen to H_2_O.^87^ To enhance stability and efficiency, these respiratory chain complexes often assemble into supramolecular structures.^88^ The proton gradient drives the translocation of protons from the intermembrane space (IMS) to the matrix *via* ATP synthase (Complex V), which catalyzes the conversion of ADP to ATP.^85,89^ Of the polypeptides involved in OXPHOS, 13 are encoded by mtDNA, while the remainder are encoded by the nuclear genome.^53,89^

#### Glucose metabolism

In glucose metabolism, glucose is initially converted to pyruvate through glycolysis in the cytoplasm. Pyruvate is then either transported into the mitochondria and converted to acetyl-CoA by pyruvate dehydrogenase or converted to lactate by lactate dehydrogenase in the cytoplasm.^90^ Acetyl-CoA, the principal substrate, enters the tricarboxylic acid (TCA) cycle.^91^ Within mitochondria, citrate synthase catalyzes the condensation of acetyl-CoA and oxaloacetate to form citrate. Citrate can either proceed through the TCA cycle, generating NADH, FADH_2_, and guanosine triphosphate (GTP), or be transported to the cytoplasm where it regenerates acetyl-CoA and oxaloacetate.^92^ When carbohydrate supply is excessive, acetyl-CoA is converted to citrate, which can then exit the mitochondria to participate in lipid synthesis or histone acetylation in the cytoplasm or nucleus.^92–94^ Additionally, certain gluconeogenesis processes occur in mitochondria, such as the conversion of pyruvate to oxaloacetate, followed by its conversion to malic acid and aspartic acid to facilitate gluconeogenesis.^95^

#### Lipid metabolism

The β-oxidation of fatty acids is another major energy source.^96^ Initially, free fatty acids (FFAs) are activated to fatty acyl-CoA in the cytosol by fatty acyl-CoA synthetase. Fatty acyl-CoA then combines with carnitine to form acylcarnitine before crossing the outer mitochondrial membrane (OMM) and IMM to enter the mitochondrial matrix.^97,98^ In the mitochondrial matrix, acylcarnitine regenerates into fatty acyl-CoA, which then undergoes β-oxidation to produce NADH/FADH_2_ and acetyl-CoA.^97^ The transport of fatty acyl-CoA is facilitated by carnitine palmitoyltransferase I (CPTI) on the OMM, carnitine palmitoyltransferase II (CPTII), and carnitine-acylcarnitine translocase (CACT) on the IMM.^99^ Some acetyl-CoA generated through β-oxidation is converted into ketone bodies in the liver, which serve as a key energy source.^98^

#### Oxidative stress

Oxidative stress occurs when the formation of reactive oxygen species (ROS) exceeds the capacity of the antioxidant defense system, which includes enzymes such as superoxide dismutase (SOD), catalase (CAT), glutathione reductase (GR), and glutathione peroxidase (GPx).^100^ This imbalance, primarily due to an excess of ROS, leads to cellular damage. ROS includes molecules such as superoxide anion (O^2−•^), hydrogen peroxide (H_2_O_2_), singlet oxygen (^1^O_2_), and hydroxyl radicals (OH•),^101,102^ which can impair mitochondrial function by damaging mtDNA, proteins, membrane lipids, and other cellular components.^103^

Mitochondria are a major source of ROS, and even under normal conditions, the ETC generates mitochondrial ROS (mtROS) as a byproduct.^104,105^ However, ROS can also originate from other sources, such as NADPH oxidase (Nox), monoamine oxidase (MAO), p66Shc, α-glycerophosphate dehydrogenase, electron transfer flavoprotein (ETF) and ETF dehydrogenase, and aconitase.^102^ The contribution of these sources varies depending on the type of ROS, the cell type, and the specific physiological or pathological conditions.^106^ Despite these other sources, mitochondria remain a significant producer of ROS and therefore merit considerable attention.

ROS plays a complex and essential role in cellular physiology. Although high concentrations of ROS are typically viewed as harmful byproducts of aerobic metabolism, at physiological levels, ROS function as key secondary messengers. They regulate various signaling pathways, including PI3K, MAPK, AMPK, NRF2, NF-κB, and p53, and influence enzyme activity, gene expression, cell proliferation, differentiation, immune responses, apoptosis, and mitochondrial quality control, allowing cells to adapt to environmental changes.^107–109^ For example, low ROS levels are critical for maintaining the self-renewal potential of stem cells.^110^ In addition, moderate ROS concentrations are vital for promoting axonal and dendritic growth, maintaining neuronal function, and supporting the self-renewal of neural stem cells and neurogenesis.^107,109^

A significant consequence of OXPHOS dysfunction is the increased production of mtROS due to decreased electron transfer efficiency in the ETC, which results in more electrons leaking and interacting with O_2_.^88^ Oxidative stress is known to play a role in the pathophysiology of mitochondrial diseases.^111^ Complexes I and III are the primary sites of mtROS production (Fig. 2). At Complex I, flavin mononucleotide (FMN) and possibly CoQ can transfer an electron to O_2_, generating O^2−•^, while at Complex III, ubisemiquinone (CoQH•) in the Qo site also contributes to O^2−•^ production.^101^ However, the amount of mtROS produced at these sites varies between tissues: the brain primarily generates mtROS at Complex I, while the heart and lungs mainly rely on Complex III.^101,104,112^ O^2−•^ can be dismutated to H_2_O_2_ by SOD or react with NO• to form peroxynitrite. H_2_O_2_ can be fully reduced to H_2_O or partially reduced to OH•.^113^

ROS disrupts cellular homeostasis by damaging lipids, proteins, and DNA.^114^ Due to their proximity, mtROS pose a significant threat to mtDNA, which, lacking histone protection, is especially vulnerable. When mtROS accumulate beyond a certain threshold, they can lead to a reduction in Δψm, which in turn may trigger the opening of the mitochondrial permeability transition pore (mPTP).^115^ The mPTP is a complex structure which spans the IMM and OMM, comprising the voltage-dependent anion channel (VDAC), adenine nucleotide translocator (ANT), ATP synthetase, and cyclophilin D.^88,116,117^ Additionally, β-tubulin regulates mPTP opening through its interaction with VDAC.^118^

Transient opening of the mPTP allows mitochondria to release excess ROS and Ca^2+^, preventing the harmful accumulation of these molecules. However, prolonged mPTP opening can lead to a secondary burst of ROS, a process known as ROS-induced ROS release.^102^ While RIRR can help eliminate irreversibly damaged mitochondria to maintain cellular homeostasis, it can also result in pathological consequences. The mPTP can also be activated by elevated Ca^2+^ levels.^102,119^ Persistent mPTP opening severely disrupts mitochondrial membrane function, becoming an important factor in mitochondrial dysfunction and ultimately leading to the activation of mitochondrial apoptosis.^118^ Beyond apoptosis, the mPTP plays a role in regulating other forms of cell death.^17^ The tendency for mPTP opening increases with aging, which further exacerbates the reduction in Δψm.^84^

### Mitochondrial quality control

**Fig. 3:**
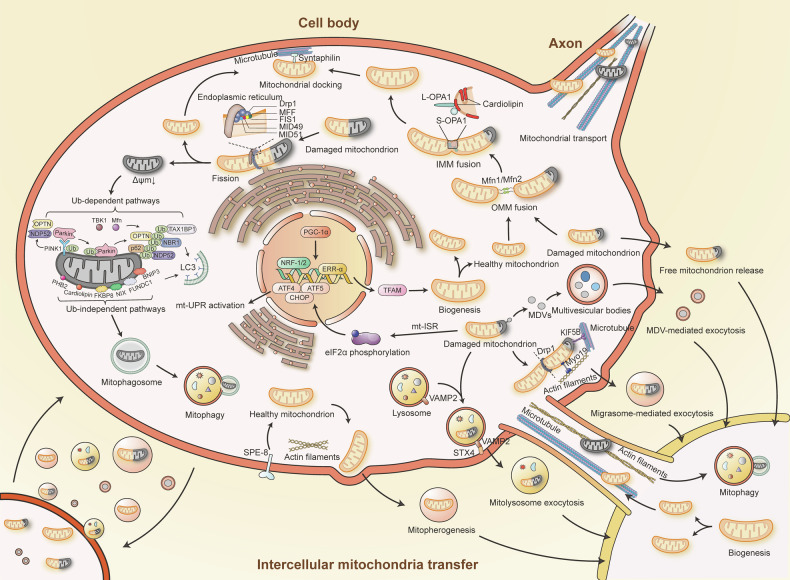
Mitochondrial Quality Control Network. Mitochondria employ both intercellular and intracellular quality control mechanisms to maintain homeostasis and redox balance. These mechanisms include mitochondrial biogenesis, fusion, fission, axonal transport, docking, mitophagy, the mitochondrial integrated stress response, and intercellular mitochondrial transfer. IMM inner mitochondrial membrane; OMM outer mitochondrial membrane; Δψm mitochondrial membrane potential; Ub ubiquitin; mt-ISR mitochondrial integrated stress response; mt-UPR mitochondrial unfolded protein response; MDV(s) mitochondria-derived vesicle(s)

Mitochondrial quality control (MQC) is a complex and integrated network that monitors mitochondrial integrity, responds to damage or stress, and maintains mitochondrial homeostasis. This system coordinates various processes, including mt-ISR, biogenesis, dynamics, mitophagy, and intercellular mitochondria transfer (Fig. 3). MQC plays a pivotal role in a wide range of human diseases, including cancer, cardiovascular diseases, and neurodegenerative disorders.^120–124^

#### Mitochondrial integrated stress response

The mt-ISR in mammals is a multifaceted mechanism that includes a transcriptional response, metabolic remodeling, and the mitochondrial unfolded protein response (mt-UPR).^125^ This response can be triggered by various mitochondrial stressors such as OXPHOS defects, reduction in Δψm, increased ROS level, amino acid deprivation, or the accumulation of unfolded proteins.^126,127^ The mt-ISR is essential for maintaining OXPHOS function and mitochondrial homeostasis through mito-nuclear communication. Key to mt-ISR are the eIF2α kinases—HRI, PKR, PERK, and GCN2—which are activated in response to mitochondrial stress.^127,128^ Activation of these kinases leads to the phosphorylation of eukaryotic translation initiation factor 2 alpha (eIF2α), which reduces global protein synthesis while selectively enhancing the translation of stress-related transcription factors like ATF4, ATF5, and CHOP, thereby activating the mt-UPR.^129–131^ This translational reprogramming helps reduce cellular energy consumption and protects cells from mitochondrial dysfunction. However, mt-ISR can also trigger apoptosis to eliminate severely damaged cells under intense stress.^128^ In the context of mitochondrial diseases, genetically defective respiratory chain complexes or mitochondrial dysfunction can activate mt-ISR, which aims to enhance ATP production and antioxidant capacity, thereby attempting to restore mitochondrial homeostasis before mitophagy is activated.^132^ While mt-ISR activation could be a common event in mitochondrial diseases, its overactivation may be detrimental, exacerbating the disease condition.^125^

#### Mitochondrial biogenesis

Mitochondrial biogenesis is the process by which mitochondria increase in number and size.^133^ This process is primarily regulated by PGC-1α (PPAR-γ coactivator-1α), a key regulator that activates nuclear respiratory factors NRF-1 and NRF-2, as well as oestrogen-related receptor-α (ERR-α).^134^ These factors enhance the expression of mitochondrial transcription factor A (TFAM, also known as mtTFA),^135,136^ which is critical for the replication and transcription of mtDNA and subsequent mitochondrial function.^135^ PGC-1β, which shares structural and functional similarities with PGC-1α, also promotes mitochondrial biogenesis.^133^ Additional regulators of this process include AMPK (AMP-activated protein kinase), nitric oxide (NO), SIRTs (sirtuins), TORCs (transducers of regulated CREB-binding protein), CaMK (calcium/calmodulin-dependent protein kinase), calcineurin, p38 MAPK, RIP140 (receptor-interacting protein 140), and Sin3A, which all influence mitochondrial biogenesis through the activation of PGC-1α.^15,133^

#### Mitochondrial dynamics

The structure of mitochondria includes the mitochondrial matrix, OMM, IMM, and IMS. The IMM is further divided into the inner boundary membrane and mitochondrial cristae. The shape and size of mitochondria vary and are closely linked to their function.^137^ Mitochondrial dynamics involve two primary processes: fusion and fission, both of which are essential for maintaining mitochondrial homeostasis and are associated with mtDNA stability, oxidative stress, apoptosis, mitophagy, and cell division.^138,139^

Fission is the process by which a single mitochondrion divides into two or more daughter mitochondria. This process allows for the segregation of healthy mitochondria from those that are old or damaged, thereby eliminating mitochondria with irreversible mtDNA damage or low Δψm to maintain cellular homeostasis.^140^ Fission is closely associated with mitophagy, the selective degradation of damaged mitochondria, which will be discussed further in the next section.^141^ Additionally, fission helps meet increased energy demands by producing more daughter mitochondria.^142^ In endothelial cells, fission also aids in the localization of mitochondria near cytoskeletal proteins to support metabolic needs.^142^ Functional fission can mitigate mitochondrial damage in mitochondrial diseases.^143^ However, excessive fission is linked to pathological outcomes, including impaired mitochondrial bioenergetics as well as induction of oxidative stress and cell death.^144^ The GTPase dynamin-related protein 1 (Drp1) is a key regulator of fission, and the endoplasmic reticulum also plays a role in this process.^145^ Drp1 is recruited to mitochondria from the cytoplasm, where it interacts with mitochondrial fission factor (MFF), mitochondrial dynamics protein of 49 kDa (MID49), MID51, and mitochondrial fission 1 protein (FIS1) on the OMM to drive the fission process.^146–148^ Additionally, FIS1 inhibits mitochondrial fusion by interacting with mitofusins, thereby inhibiting their GTPase activity.^149^

Mitochondrial fusion is the process where two or more mitochondria come into close contact and merge their IMM and OMM. This process relies on two key GTPases: optic atrophy protein 1 (OPA1) and mitofusins 1 and 2 (Mfn1 and Mfn2).^150,151^ Fusion begins with the merging of the OMM, driven by Mfn1 and Mfn2, which are localized on the OMM. After the OMM fusion, long OPA1 (L-OPA1) interacts with cardiolipin to facilitate the fusion of the IMM.^152,153^ There is also a short form of OPA1 (S-OPA1) produced by the proteolytic cleavage of L-OPA.^153^ The interaction between S-OPA1 and L-OPA1 promotes the fusion of the IMM.^154,155^ Fusion plays a pivotal role in mitochondrial heteroplasmy, which refers to the co-existence of mutant and wild-type (healthy) mitochondria.^156^ Defective mitochondria with mutant mtDNA can fuse with healthy mitochondria, compensating for defects by sharing components such as transcripts, thus mitigating the effects of mutations (heteroplasmy). Similarly, two defective mitochondria can fuse to cross-complement each other. Therefore, mitochondrial fusion can rescue certain dysfunctions if the mutation remains within a critical threshold.^157,158^ Fusion is often viewed as a defensive response, enabling mitochondria to adapt to cellular stress by reducing mtDNA heteroplasmy, bridging Δψm, and exchanging various metabolic intermediates.^142^

Mitochondrial transport is an ATP-dependent process that is especially critical in neurons. This transport occurs in both anterograde and retrograde directions along microtubules.^159^ Anterograde transport provides healthy and robust mitochondria from soma for distal axon, while damaged mitochondria in these distal regions are retrogradely transported to the soma for repair and degradation.^160^ Long-distance mitochondrial transport along microtubules is facilitated by two types of motor proteins: kinesin and dynein. In neurons, axonal microtubules are oriented with their minus ends toward the soma and their plus ends toward the distal axon. The minus-end-directed dynein drives retrograde transport, while the plus-end-directed kinesin (mainly KIF5) controls anterograde transport.^161^ The kinesin and dynein should interact with their motor adaptors before transport. For anterograde transport, the motor adaptor complex consisting of Miro (an atypical Rho GTPase) and Milton (TRAK in mammals) connects kinesin to mitochondria. Retrograde transport is primarily mediated by dynein and its motor adaptor, dynactin. The initiation of retrograde transport is believed to involve cooperation between the dynein-dynactin complex, VDAC on the OMM, and the Milton-Miro complex.^162^ Additionally, Drp1, Mfn1, and Mfn2 are thought to play roles in mitochondrial transport.^160^ There is also short-distance mitochondrial movement along actin filaments within dendritic spines, which is mediated by myosins.^162^

In contrast to transport, mitochondrial docking ensures that mitochondria remain in place to maintain stable mitochondrial numbers, adequate ATP production, and meet metabolic demands, particularly in regions with high energy requirements and metabolic stress.^161^ Syntaphilin, an anchoring protein, binds to the OMM and attaches axonal mitochondria to microtubules, resulting in mitochondrial docking.^160^

#### Mitophagy

Mitophagy is the process by which damaged mitochondria are delivered to lysosomes for degradation, a concept first introduced by Lemasters as a specific form of organelle autophagy.^163^ Unlike mitochondrial biogenesis, which generates new mitochondria, mitophagy removes damaged or unnecessary mitochondria to maintain cellular homeostasis, often through a selection process mediated by mitochondrial fission.^164^ However, excessive mitophagy can lead to a significant loss of mitochondrial content, potentially triggering cell death.^165,166^ The interaction between Drp1 and Zip1 (a mitochondrial zinc transporter) at the fission site facilitates Zn^2+^ entry into the mitochondrial matrix, resulting in a localized reduction of Δψm, which subsequently initiates mitophagy in the affected mitochondria.^167^ Interestingly, mtDNA mutations alone may not be sufficient to trigger mitophagy.^168^ ROS plays a critical role in activating mitophagy, and in turn, mitophagy helps regulate ROS levels.^169^ Excessive ROS can induce non-selective autophagy in response to oxidative stress, while mild oxidative stress typically triggers selective mitophagy that is dependent on mitochondrial fission.^170^ The mitophagy process involves several key steps: the reduction of Δψm, formation of the mitophagosome, delivery of the mitophagosome to the lysosome, and finally, the degradation and recycling of mitochondrial components.^171,172^

Mitophagy mechanisms are generally categorized into ubiquitin (Ub)-dependent and Ub-independent pathways.

Among the Ub-dependent pathways, the phosphatase and tensin homolog-induced putative kinase 1 (PINK1)-Parkin pathway is the most extensively studied. In mitochondria with normal Δψm, PINK1 is transported to the IMM, where it is cleaved and degraded.^173^ However, when Δψm is reduced, PINK1 cannot reach the IMM and instead accumulates on the OMM. There, PINK1 phosphorylates ubiquitin and recruits and phosphorylates the E3 ubiquitin ligase Parkin.^174,175^ Once phosphorylated, Parkin binds to Ser65-phosphorylated ubiquitin on the OMM, fully activating its E3 ubiquitin ligase activity. This activation amplifies the pathway, promoting mitophagy.^176^ Mitochondria tagged with phosphorylated poly-Ub chains by Parkin are recognized by ubiquitin-binding receptor proteins such as OPTN, NDP52, SQSTM1/p62, TAX1BP1, and NBR1,^177–179^ which then bind with LC3 to initiate mitophagy. Additionally, TBK1 and Mfn2 have been shown to participate in this pathway.^180,181^ Beyond the PINK1-Parkin pathway, there are Parkin-independent, ubiquitin-dependent pathways where PINK1 directly recruits NDP52 and OPTN.^177^

Mitophagy receptors such as Fun14 domain containing 1 (FUNDC1), BCL2 interacting protein 3 (BNIP3), BCL2 interacting protein 3 like (BNIP3L/NIX), FKBP prolyl isomerase 8 (FKBP8), and ATAD3B can directly interact with LC3 through their LIR (LC3-interacting region) motifs, thereby initiating ubiquitin-independent mitophagy. Additionally, PHB2 and cardiolipin also participate in the ubiquitin-independent pathway due to their translocation to the OMM.^171,182,183^

#### Intercellular mitochondria transfer

Intercellular mitochondria transfer, where mitochondria are exchanged between donor and recipient cells, is another key component of MQC. The mitochondria transfer between cells with normal mitochondria and cells with dysfunctional mitochondria can rescue mitochondrial respiration defects.^43^ Mitochondria transfer is believed to aid in coping with cellular stress by facilitating intercellular communication under both physiological and pathological conditions.^16^ In this process, stressed donor cells transfer damaged mitochondria to healthy recipient cells. The recipient cells, upon receiving the damaged mitochondria, trigger mitochondrial biogenesis and fission to regenerate healthy mitochondria, which can then be re-transferred to the stressed donor cells. Additionally, stressed donor cells can transfer damaged mitochondria to other cells to initiate transcellular mitophagy (autophagy), especially when the stress or damage exceeds their metabolic capacity.^16,184,185^ There are three major routes of mitochondria transfer: tunneling nanotubes (TNT), mitochondrial extracellular vesicles (mitoEVs), and free mitochondria release.

Mitochondria can be shuttled across TNTs along either microtubules or actin filaments. Kinesin and its motor adaptor, the Miro-Milton complex, facilitate movement along microtubules, while myosin mediates transfer along actin filaments by interacting with Miro and anchoring mitochondria to the actin filaments.^184,185^

In addition to conventional extracellular vesicles (EVs), mitochondria produce specialized vesicles known as mitochondria-derived vesicles (MDVs), which encapsulate mtDNA and other mitochondrial components.^186^ MDVs primarily fuse with multivesicular bodies, such as late endosomes and lysosomes, although a select few are secreted into the extracellular space *via* a process driven by OPA1 and Snx9.^187^

Mitolysosome exocytosis, a mitoEVs-related MQC mechanism first observed in flunarizine-induced Parkinsonism-like symptoms, eliminates mitochondria through a mitophagy-independent pathway.^188^ During this process, mitochondria are directly engulfed by lysosomes and extruded from the cell without the formation of autophagosomes.^189^ Flunarizine-induced impairment of OXPHOS and the collapse of Δψm are believed to trigger this process. Proteins such as BAX, a mediator of mitochondrial outer membrane permeabilization (MOMP), and NDUFS4, a complex I subunit, may facilitate mitochondrial entry into lysosomes.^188^ Once inside lysosomes, the extracellular secretion of mitochondria is mediated by a VAMP2 (vesicle-associated membrane protein 2)-STX4 (syntaxin-4)-dependent mechanism.^188^

Migrasome-mediated exocytosis is also an emerging MQC mechanism involving mitoEVs.^190^ Migrasomes, defined as vesicles containing cytosolic contents, form on retraction fibers during cell migration.^191,192^ This process enables cells to clear damaged mitochondria, which may harbor detrimental mutant mtDNA, reduced Δψm, or elevated ROS levels, ensuring mitochondrial quality. Key factors in this process include Myosin19 (Myo19), KIF5B, and Drp1. Damaged mitochondria are transported to the plasma membrane by KIF5B, where Myo19 anchors them to cortical actin before Drp1-driven fission occurs. The reduced recruitment of dynein, the inward motor on microtubules, prevents damaged mitochondria from retracting back, ultimately leading to their incorporation into migrasomes.^190^ Migrasome formation relies on a reconstituted membrane system rich in tetraspanins and cholesterol.^193^ Migrasome-mediated exocytosis plays a role in maintaining homeostasis, particularly when the damage is insufficient to trigger mitophagy, and is especially important in migrating cells.

Mitopherogenesis is a specialized form of mitochondria-specific ectocytosis identified in sperm, which functions to control mitochondrial quantity. Unlike other exocytosis processes, mitopherogenesis involves the secretion of healthy mitochondria through EVs, with each vesicle carrying a single healthy mitochondrion. Proper actin-filament dynamics, extracellular protease activity, and the tyrosine kinase SPE-8 significantly influence this process,^194^ highlighting its potential impact on MQC.

Finally, free mitochondria released without membrane encapsulation occur in cases of mitophagy dysfunction.^195^ Damaged mitochondria within mitophagosomes lacking mammalian ATG8 conjugation cannot be degraded by lysosomes and instead undergo autophagic secretion, transferring the damaged mitochondria to healthy cells for transcellular degradation.^195^

### Mitochondrial apoptosis

**Fig. 4:**
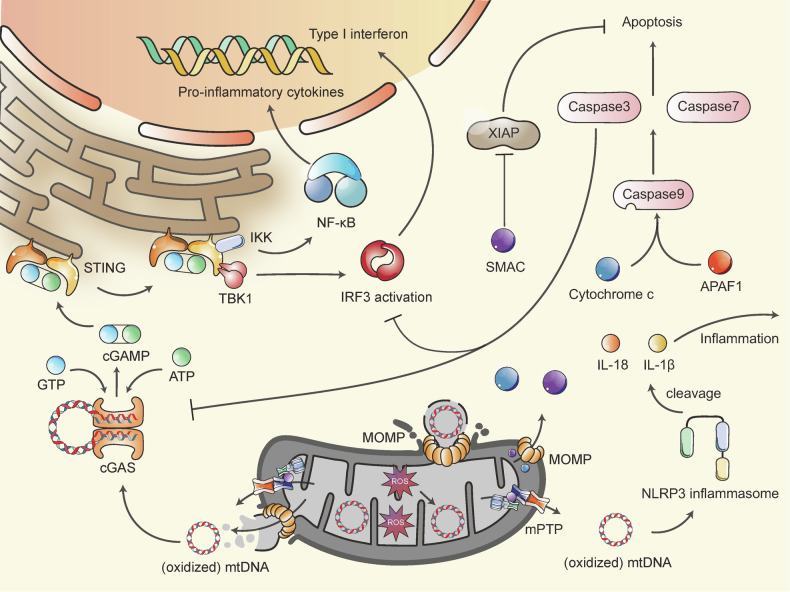
Mitochondrial Apoptosis and Inflammation. Components within the IMS or matrix can trigger apoptosis or inflammation if leaked into the cytosol, primarily due to the mPTP and MOMP. Upon MOMP formation and mPTP opening, cytochrome C, SMAC, and mtDNA are released into the cytosol. Cytochrome C interacts with APAF1, activating caspase 9 to initiate the caspase cascade leading to apoptosis. SMAC accelerates this process by inhibiting XIAP. After binding with mtDNA, the cGAS enzyme produces cGAMP from ATP and GTP, activating the cGAS-STING signaling pathway and inducing type I interferon expression and NF-κB activation. The NLRP3 inflammasome can also bind with (oxidized) mtDNA to promote IL-1β and IL-18 cleavage. However, caspase 3 cleaves cGAS and IRF3 during apoptosis, inhibiting inflammation. MOMP mitochondrial outer membrane permeabilization; mPTP mitochondrial permeability transition pore; SMAC second mitochondrial-derived activator of caspases; APAF1 apoptosis protease activating factor 1; XIAP X-linked inhibitor of apoptosis protein; cGAS cyclic GMP-AMP synthase; STING stimulator of interferon genes; cGAMP cyclic guanosine monophosphate–adenosine monophosphate; TBK1 TANK binding kinase 1; IKK IκB kinase; IRF3 interferon regulatory factor 3

Under stress, cells may initiate apoptosis as a mechanism to manage these adverse situations.^196^ Apoptosis can occur through mitochondrial pathway (Fig. 4), thereby playing a role in the pathogenesis of mitochondrial diseases.^111,197,198^

The mitochondrial apoptosis pathway is intricately linked to MOMP, which is predominantly regulated by BCL-2 family proteins, though it can also be triggered by the mPTP.^116,196^ The BCL-2 family is divided into pro-apoptotic and anti-apoptotic members. Pro-apoptotic BCL-2 proteins include BH3-only proteins (such as BID, BIK, and BIM) and effectors like BAK, BAX, and BOX, while anti-apoptotic proteins include BCL-2, BCL-X, BCL-W, A1, and MCL1. BH3-only proteins respond to apoptotic stimuli by activating effectors, typically BAX and BAK.^199,200^ Once activated, BAX and BAK accumulate on the OMM, dimerize, and form higher-order oligomers that create pores in the OMM, leading to MOMP.^201^ MOMP results in the release of IMS proteins, such as cytochrome c and the second mitochondrial-derived activator of caspases (SMAC), into the cytoplasm. Cytochrome c binds to apoptosis protease activating factor 1 (APAF1) in the cytoplasm, and together, they activate caspase 9. Activated caspase 9 then activates caspases 3 and 7, which ultimately drive the apoptotic process. Additionally, SMAC enhances the caspase cascade by inhibiting the X-linked inhibitor of apoptosis protein (XIAP).^199,202,203^ Other forms of programmed cell death, such as necroptosis, pyroptosis, and ferroptosis, are also related to mitochondria.^204^

### Mitochondrial inflammation

Mitochondria can initiate inflammation by releasing damage-associated molecular patterns (DAMPs) due to their evolutionary similarities to bacterial pathogen-associated molecular patterns when subjected to stress or damage. These DAMPs include N-formyl peptides, TFAM, cardiolipin, ATP, ROS, and mtDNA.^205^ In addition to the inflammatory effects of mtDNA, which will be discussed in detail, other DAMPs have also been recognized for their roles in promoting inflammation.^206,207^

Under conditions of mitochondrial stress or dysfunction, mtDNA can be released into the cytosol or extracellular space.^208,209^ MtDNA can trigger pro-inflammatory and type I interferon responses, which vary depending on the cell type and context.^210^ The release of mtDNA into the cytoplasm occurs *via* MOMP and the opening of the mPTP.^210^ It is likely that mPTP and MOMP work together to facilitate mtDNA release.^208^ The inflammation mediated by mtDNA is primarily driven by the cGAS (cyclic GMP-AMP synthase)-STING (stimulator of interferon genes) signaling pathway (Fig. 4). When cGAS binds to mtDNA, it catalyzes the production of cyclic guanosine monophosphate–adenosine monophosphate (cGAMP) from ATP and GTP.^211^ cGAMP then activates STING, which subsequently recruits and activates TANK binding kinase 1 (TBK1). TBK1 phosphorylates STING, leading to the activation of transcription factors interferon regulatory factor 3 (IRF3), leading to type I interferon expression.^212–214^ STING also phosphorylates IκB kinase (IKK) to initiate NF-κB pathway, resulting in increased production of pro-inflammatory cytokines.^208^ In addition to the cGAS-STING pathway, the mtROS and mtDNA can activate NLRP3 inflammasome to promote IL-1β and IL-18 cleavage.^17^ Notably, low levels of mtDNA-induced inflammation, caused by minor MOMP, can aid in fighting infections,^215^ while simultaneous activation of caspase 3 following MOMP can inhibit mtDNA-induced inflammation by cleaving cGAS and IRF3.^216,217^

A connection between inflammation and mitochondrial bioenergetics has been established, indicating that mitochondrial dysfunction can exacerbate inflammation, which in turn impairs OXPHOS and disrupts MQC.^218^ An inflammatory transcriptomic profile has been observed in peripheral blood mononuclear cells of patients with mitochondrial diseases.^219^ Although research on the role of inflammation in mitochondrial diseases is still limited, inflammation is a critical factor in many human diseases, such as neurodegenerative disorders.^220^ Its potential pathological effects on mitochondrial diseases warrant further investigation.

In summary, mitochondria are central to metabolism, stress response, inflammation, and other critical cellular processes. Energy stress, characterized by reduced intracellular ATP levels, is a key feature of many human diseases.^221,222^ Mitochondrial genetic defects that disrupt energy metabolism lead to protein synthesis defects, ATP insufficiency, and mtROS overproduction.^81^ Despite the genetic heterogeneity of mitochondrial diseases, the resulting protein, energy, and oxidative stress are consistent.^223^ Cells initiate various MQC mechanisms to restore normal bioenergetics and redox balance in response to stress.^224^ However, when damage exceeds the capacity of MQC system, stress-induced mPTP opening and MOMP can release mtDNA or other DAMPs into the cytoplasm, triggering inflammation and cell death.^209,225^ Understanding how cells respond to stress induced by mitochondrial genetic defects may provide insights into the onset and progression of these diseases.

## Molecular mechanisms of mitochondrial diseases

**Fig. 5:**
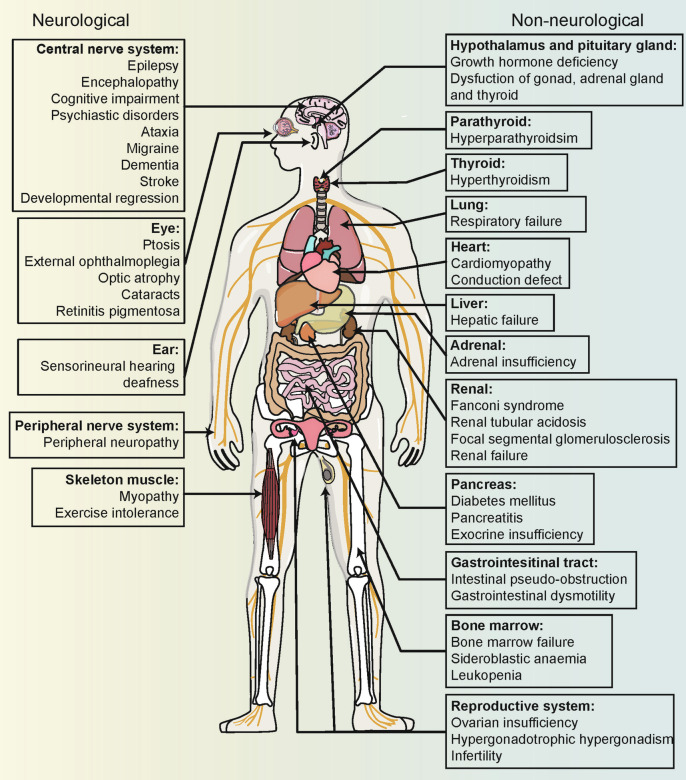
Multisystem Clinical Presentation of Mitochondrial Diseases. Due to the ubiquitous presence of mitochondria, mitochondrial diseases can present in any tissue of the body. Tissues and organs with high energy demands, such as skeletal muscle and brain, are particularly susceptible to oxidative phosphorylation defects, leading to common manifestations like myopathy and encephalopathy in mitochondrial diseases. The diverse and variable symptoms associated with these conditions increase the risk of misdiagnosis

Mitochondrial diseases are characterized by primary or secondary defects in mitochondrial function or structure, resulting from mutations in either nDNA or mtDNA.^226^ Specifically, 36 pathogenic genes (11%) are encoded by mtDNA, while 302 (89%) are encoded by nDNA.^227^ Heteroplasmy is present in most healthy individuals without leading to mitochondrial disease.^228^ However, symptoms manifest only when the proportion of mutant mtDNA surpasses a certain threshold.^229^ Due to the considerable genetic and clinical heterogeneity, mitochondrial diseases can affect either single or multiple organ systems, leading to a wide range of clinical manifestations (Fig. 5). Tissues with high energy demands are particularly susceptible to energy deficits and are therefore most commonly affected.^230^ Additionally, the age of onset and severity of these diseases can vary significantly depending on the degree of heteroplasmy and individual differences.^4^

### The primary mitochondrial diseases caused by mtDNA mutations

The hallmark of mitochondrial diseases resulting from mtDNA mutations is the primary disturbance of OXPHOS. This disturbance typically leads to energy deficits, increased oxidative stress, and a collapse of the Δψm.

#### Leber hereditary optic neuropathy

LHON is the most prevalent mitochondrial disease, characterized by maternal inheritance and a pronounced male sex bias.^231^ It was the first disease definitively linked to mtDNA mutations.^33^ The primary clinical manifestation of LHON is the bilateral, severe loss of central vision, caused by degeneration of retinal ganglion cells (RGCs).^232^ The high energy demands of RGCs may explain the cell-specific vulnerability in the eye. Retina is one of the most energy-consuming tissues in the body.^233^ RGCs are located in the retina, and their axons form the optic nerve. Based on mitochondrial distribution and energy consumption, RGCs can be divided into four subcellular components: dendrites, cell body, unmyelinated axon (including intraocular and optic nerve head segments), and myelinated axon (post-lamina cribrosa).^233^ RGCs, with their exceptionally long axons and high frequency of action potentials, require substantial energy. Mitochondrial transport along these extensive axons consumes significant amounts of ATP to sustain axonal function.^197,233^ Additionally, the continuous conduction of action potentials in unmyelinated axons demands more energy than the saltatory conduction in myelinated axons,^197,234^ making the unmyelinated segments before the lamina cribrosa particularly susceptible to energy deficits.^235^ Mitochondrial density correlates with energy demand,^233^ resulting in an uneven distribution where mitochondria are abundant in the cell body and unmyelinated axons but sparse in the myelinated axons.^236^ Compounding this vulnerability, exposure to light, particularly short-wave or blue light (400–480 nm),^237^ can negatively impact OXPHOS in RGCs, leading to decreased ATP production and increased ROS levels.^237^

Over 95% of patients with LHON carry one of three common mtDNA point mutations: m.3460 G > A, m.11778 G > A, or m.14484 T > C.^231^ These mutations occur in the *MT-ND1*, *MT-ND4*, and *MT-ND6* genes, respectively, all of which encode subunits of complex I.^238^ While ATP synthesis deficits are evident, energy failure alone may not be the primary cause of RGC degeneration in LHON.^231^ Instead, the dysfunctional complex I likely increases mtROS production, which in turn induces cellular apoptosis, playing a critical role in RGC degeneration.^235,239^ Mitochondrial apoptosis is pivotal in the pathogenesis of LHON.^240^ For instance, the m.3460 G > A mutation in *MT-ND1* is associated with elevated mtROS production and increased levels of pro-apoptotic proteins such as cytochrome c, BAK, BAX, PARP, and cleaved caspases 3, 7, and 9.^241^ In addition to ROS, energy failure can activate apoptosis-inducing factors and endonuclease G, triggering apoptosis through a caspase-independent pathway.^242^ Overproduction of mtROS can directly induce cytochrome c release by prolonging mPTP opening or indirectly trigger cytochrome c release through caspase cascade activation and MOMP, leading to apoptosis in RGCs.^243^ Mito-nuclear communication also plays a role in LHON pathogenesis, as evidenced by the activation of mt-ISR transcripts in LHON, which leads to chronic inhibition of protein synthesis, affecting synaptic and oligodendroglial function, and contributing to disease progression.^244,245^ Besides, the respiratory chain complex I defect can cause remodeling of one-carbon metabolism through mt-ISR.^125,246^ This one-carbon metabolic remodeling undermines NADPH production, which sensitizes affected cells to oxidative stress and may facilitate inflammation and cell death.^10^ However, the anti-apoptotic protein XIAP can prevent RGC apoptosis by inhibiting mitochondrial apoptosis.^247^

Excessive mitophagy, driven by AMPK activation, has been shown to promote apoptosis and may lead to the widespread and near-synchronous death of RGCs in subacute LHON. In contrast, overexpression of PGC-1α, which facilitates mitochondrial biogenesis, has been found to prevent cell apoptosis.^248^ Increased mitochondrial biogenesis is thought to contribute to the phenomenon of incomplete penetrance in LHON and serves as a compensatory mechanism to restore mitochondrial turnover in LHON carriers.^249^ Thus, balancing mitochondrial biogenesis and mitophagy could represent a potential therapeutic target. Additionally, mitochondrial transport, an ATP-dependent process critical for maintaining axonal mitochondrial homeostasis, has been found to be impaired in LHON, potentially contributing to axonal loss.^250^

Significant progress has been made in developing therapies for LHON. Techniques such as allotopic expression and gene editing hold great promise for future treatments. Currently, Idebenone is the only drug approved by the European Medicines Agency for improving visual impairment in patients with LHON.^198,251^

#### Mitochondrial myopathy, encephalopathy, lactic acidosis, and stroke-like episodes

Mitochondrial myopathy, encephalopathy, *lactic acidosis*, and stroke-like episodes (MELAS) manifest as lactic acidosis, headaches accompanied by nausea and vomiting, epilepsy, and stroke-like episodes, all inherited maternally.^252^ Additional symptoms observed in MELAS include deafness, diabetes mellitus, gastrointestinal disturbances, ataxia, growth failure, myopathy, and cardiomyopathy.^253,254^ The primary genetic cause of MELAS is the m.3243 A > G mutation in the *MT-TL1* gene, which encodes mt-tRNA^Leu(UUR)^,^255^ accounting for over 80% of cases.^255^ Another mutation, m.3271 T > C in *MT-TL1*, is responsible for 7–15% of MELAS cases,^256,257^ while other mtDNA mutations associated with MELAS are rare. Mutations in the *MT-TL1* gene can lead to defects in the aminoacylation of mt-tRNA^Leu(UUR)^, disrupting the interaction between mRNA and ribosomes.^258^ This abnormal translation impairs the synthesis of respiratory chain complexes, particularly complexes I and IV.^259^

Oxidative stress is believed to play a significant role in MELAS pathogenesis.^111^ Increased levels of ROS, apoptosis, and inflammation have been observed in diseased endothelial cells from patients with MELAS carrying the m.3243 A > G mutation.^260^ Similar endogenous oxidative stress has been found in fibroblasts and induced pluripotent stem cells (iPSCs) from patients with MELAS.^183^

In neuronal cells, complex I defects predominate, and active degradation of complex I *via* mitophagy is a protective response to mitochondrial dysfunction during neuronal differentiation.^261^ This degradation reduces mtROS production, acting as a protective mechanism.^261^ However, normal lysosomal function and the sequestration of cytosolic components during autophagy depend heavily on adequate ATP levels.^262^ In MELAS fibroblasts, although mitophagy is activated, autophagic flux is reduced, and autophagosome elimination is defective, likely due to ATP deficiency.^263^ This reduction in mitophagy can lead to the accumulation of damaged mitochondria with defective complex I. Interestingly, mt-tRNA fragments, a class of ncRNAs, have been implicated in extracellular lactate accumulation in MELAS, possibly through the downregulation of mitochondrial pyruvate carrier 1.^69^ Furthermore, the oxidative stress induced by primary tRNA^Leu(UUR)^ defects can exacerbate impaired tRNA modification.^264^ Stress-induced microRNA-9/9* has been shown to post-transcriptionally suppress mt-tRNA-modification enzymes, leading to reduced U34 modification of non-mutant tRNAs and promoting mitochondrial dysfunction.^264^

Current treatments for MELAS are symptomatic and include the supplementation of antioxidants and cofactors, anti-epileptic drugs, and lactate-lowering agents. However, there remains no consensus on the optimal treatment approach for MELAS.^265,266^

#### Maternally inherited diabetes and deafness

Maternally inherited diabetes and deafness (MIDD) is another mitochondrial disease frequently associated with the m.3243 A > G mutation in the *MT-TL1* gene.^267^ The most prominent clinical features of MIDD are diabetes and hearing loss,^268^ although other complications such as myopathy, neuropathy, oculopathy, cardiac disease, and nephropathy are also observed.^269^ Approximately 85% of MIDD cases are caused by the m.3243 A > G point mutation.^269^ Notably, this same mutation is also responsible for MELAS. Interestingly, there have been case reports of MIDD evolving into MELAS over time,^270^ suggesting that these conditions represent different phenotypes within the same disease spectrum influenced by the level of mtDNA heteroplasmy: MELAS typically occurs with higher levels of mtDNA heteroplasmy (typically >85%), while MIDD is associated with lower levels (typically <45%).^271^

Insulin production demands significant ATP, and the decreased ATP generation coupled with increased ROS production in pancreatic β-cells with dysfunctional mitochondria leads to a gradual decline in insulin secretion, eventually resulting in insulin deficiency.^272^ Oxidative stress also plays a critical role in hearing loss,^273^ with ROS-induced activation of the AMPK-E2F1 pathway promoting apoptosis in the stria vascularis and spiral ganglion neurons.^274^ Additionally, the role of nuclear factors in the phenotypic variability of the m.3243 A > G mutation has been increasingly recognized.^275^

Managing blood glucose levels is the primary focus of treatment for MIDD. Since insulin sensitivity is generally preserved, oral hypoglycemic drugs, including insulin secretagogues, should be considered. However, metformin should be avoided due to the increased risk of lactic acidosis.^272^

#### Myoclonic epilepsy with ragged red fiber

Myoclonic epilepsy with ragged red fibers (MERRF) is a mitochondrial disorder defined by the presence of progressive myoclonus epilepsy and ragged-red fibers (RRF) observed in muscle biopsies.^276^ This disease also manifests with symptoms like ataxia, cardiomyopathy, lipomatosis, and dementia.^277^ In 1990, a significant association was identified between MERRF and the m.8344 A > G point mutation in the *MT-TK* gene, responsible for encoding mt-tRNA^Lys(UUR)^.^278^ This mutation, m.8344 A > G, is the most prevalent pathogenic variant, accounting for over 80% of cases.^279^ It hinders the N^1^-methyladenosine (m^1^A) modification at position 58 in mt-tRNA^Lys(UUR)^,^280^ leading to defects in mt-tRNA^Lys(UUR)^, which subsequently disrupts the synthesis of respiratory chain complexes, primarily complexes I and IV, resulting in mitochondrial dysfunction.^281^ Recently, mutations in the *MT-RNR1* and *MT-RNR2* genes, which encode 12S rRNA and 16S rRNA, respectively, have also been linked to MERRF.^282^ These rRNAs are integral to mitochondrial ribosomes and the mtDNA-encoded mRNAs.^282^

The neurological defects observed in MERRF may be related to compromised synaptic plasticity in excitatory neurons, a consequence of mitochondrial dysfunction and synaptic impairment.^283^ The study utilizing human iPSCs derived from patients harboring the m.8344 A > G mutation reveals mitochondrial fragmentation, reduced oxygen consumption, and elevated ROS production.^284^ Furthermore, neurons induced from patients with MERRF exhibit smaller, rounded, and fragmented mitochondria with decreased Δψm, increased ROS. The activated mitophagy and impaired autophagy flux are also present in these neurons.^285^ Despite the activation of autophagy and mitophagy in MERRF, their flux remains impaired, which is associated with ATP deficiency.^286^ Treatment with coenzyme Q10 (CoQ10) has been shown to enhance mitochondrial function in MERRF fibroblasts and cybrids by promoting autophagy flux,^286^ indicating that reduced mitophagy and autophagy flux might be critical contributors to MERRF pathogenesis. Notably, no apoptotic changes are observed in the muscles of mitochondrial encephalomyopathies, possibly due to sarcoplasmic expression of XIAP.^287,288^ However, the exact mechanisms underlying this apoptosis suspension require further investigation. Importantly, defective tRNAs can lead to the accumulation of damaged and unfolded mitochondrial proteins,^289,290^ suggesting that targeting mt-ISR could be a potential therapeutic strategy for MERRF and other mitochondrial disorders caused by tRNA defects. Continued research into the molecular etiology of these conditions is essential.

#### Neurogenic muscle weakness, ataxia, and retinitis pigmentosa syndrome

Neurogenic muscle weakness, ataxia, and retinitis pigmentosa (NARP) syndrome is a maternally inherited disorder characterized by a range of symptoms including muscle weakness, sensory neuropathy, ataxia, seizures, dementia, retinitis pigmentosa, optic atrophy, and developmental delay.^291^

Mutations m.8993 T > G or m.8993 T > C in the *MT-ATP6* gene are implicated in the pathogenesis of NARP.^291–293^ These mutations are also associated with Leigh syndrome,^294,295^ with the specific phenotype determined by the level of heteroplasmy. A heteroplasmy level exceeding 85% for the m.8993 T > G mutation is predominantly linked to childhood-onset Leigh syndrome, whereas a level of 60–70% typically results in adult-onset NARP. Both diseases may occur with heteroplasmy levels between 70–85%.^234,296^ The m.8993 T > G mutation leads to the substitution of a highly conserved leucine with arginine (while m.8993 T > C replaces leucine with proline) in the ATPase6-encoded proton channel, impairing the proton translocation mechanism of ATP synthase (complex V) and subsequently disturbing OXPHOS.^234^

In addition to role in OXPHOS, the ATP synthase subunits e and g are important to the formation of mitochondrial cristae.^297^ Dissolution of mitochondrial cristae has been observed in patients with the m.8993 T > G mutation.^298^ Additionally, ATP synthase is a component of mPTP.^88^ Aberrant mitochondrial cristae and mPTP opening may lead to the release of mtDNA and IMS proteins, such as cytochrome c, thereby triggering mitochondrial apoptosis and inflammation.^299^ This hypothesis warrants further investigation. In yeast cells harboring defective *MT-ATP6*, IMM fusion is inhibited. It is plausible that dysfunctional IMM fusion could be a common feature in all diseases with genetically defective OXPHOS, potentially initiating mitophagy to eliminate defective mtDNA.^300^ The molecular basis underlying cerebellar atrophy in patients with the m.8993 T > G or m.8993 T > C mutations remains unclear. However, in a mouse model of inherited Purkinje cell degeneration, increased mitophagy and autophagy have been associated with Purkinje cell loss.^301^

#### Progressive external ophthalmoplegia

Progressive external ophthalmoplegia (PEO), also known as chronic progressive external ophthalmoplegia (CPEO), is a prevalent clinical syndrome within mitochondrial diseases, characterized by progressive bilateral ptosis and diffuse, symmetric ophthalmoparesis.^302^ PEO can be classified into three phenotypes: pure PEO (isolated occurrence), Kearns-Sayre syndrome, and PEO-plus syndrome, where myopathy or other extraocular symptoms are present.^303^ The most common cause of PEO is a single large-scale mtDNA deletion,^303^ placing it within the category of mitochondrial diseases caused by mtDNA mutations.

Typically, single large-scale mtDNA deletions arise sporadically rather than through maternal inheritance, resulting from the amplification of a single mutation associated with spontaneous errors in DNA polymerase γ during early embryonic development.^304,305^ The age of onset and severity of the disease are correlated with the size of the deletion, the level of heteroplasmy, and the specific region affected within the mtDNA.^306^ These deletions, whether singular or multiple, can impair the function of one or more mtDNA-encoded proteins, resulting in OXPHOS disturbance.

PEO can also be attributed to multiple mtDNA deletions or depletion, secondary to nDNA mutations, which follow either autosomal recessive or dominant inheritance patterns and involve genes such as *POLG, POLG2, SLC25A4, C10orf2, SPG7, DNA2, RNASEH1, TOP3A, TK2, DGUOK, RRM2B, GMPR, LIG3*, and *RRM1*.^307^ Generally, mtDNA depletion is associated with early-onset disorders that typically have a fatal course, whereas adult-onset disorders are more commonly linked to multiple mtDNA deletions.^308^

The minimal mitochondrial replisome consists of polymerase γ, Twinkle, the mitochondrial single-stranded DNA-binding protein (mtSSB), and the mitochondrial RNA polymerase.^309^ DNA polymerase γ, the only polymerase responsible for mtDNA replication,^310^ comprises three subunits encoded by two nuclear genes: the p140 catalytic subunit encoded by *POLG* (*POLG1*) and the p55 accessory subunit encoded by *POLG2*.^311^ Mutations in *POLG* or *POLG2* genes can lead to mtDNA defects. Specifically, mutations in *POLG* compromise mitochondrial genetic integrity, resulting in multiple deletions that contribute to PEO.^310^ Similarly, *POLG2* mutations impair the proper stimulation of p140, disrupting mtDNA replication.^312^ The deletion pattern associated with *POLG* mutations may result from faulty strand displacement replication, initiated by replication fork stalling.^313^

Twinkle, encoded by *C10orf2*, is essential for nascent H-strand synthesis in the D-loop and is thus indispensable for mtDNA replication, despite the existence of other potential mtDNA helicases.^314^ A PEO mouse model with a *C10orf2* defect has been established,^315^ demonstrating that type IIB fibers of extraocular muscles are particularly vulnerable to mtDNA deletions, likely due to their relatively low mitochondrial content, which allows even a few mutant mtDNA to surpass the onset threshold.^316^ The endosomal-mitophagy pathway, involving ATAD3, VPS35, SAMM50, and BAK/BAX, plays a role in mtDNA deletion caused by *C10orf2* mutations.^317^ Furthermore, persistent activation of the mTORC1-mt-ISR pathway due to mtDNA replication defects disrupts cellular metabolic homeostasis, thereby contributing to disease progression.^125^ Thymidine kinase 2 (TK2) is a mitochondrial enzyme responsible for catalyzing the conversion of deoxycytidine and deoxythymidine nucleosides to their nucleoside monophosphates, which are then converted into deoxynucleoside triphosphates.^318^ Mutations in the *TK2* gene disrupt the maintenance of the mitochondrial deoxyribonucleotide pool, leading to mtDNA depletion or multiple mtDNA deletions, and subsequently resulting in PEO.^318,319^

For patients with pure PEO, surgery remains the primary treatment option, whereas symptomatic treatment is recommended for extraocular symptoms in PEO-plus syndrome.^302^

#### Kearns-Sayre syndrome

Kearns-Sayre syndrome was first identified in 1958 by Kearns and Sayre, characterized by the triad of retinitis pigmentosa, PEO, and complete heart block.^320^ It is classified as one of the mitochondrial encephalomyopathies and presents with additional common symptoms, including cerebellar ataxia, cerebrospinal fluid protein levels exceeding 100 mg/dL, deafness, dementia, diabetes, delayed puberty, and amenorrhea.^321,322^ An onset before the age of 20 is a defining feature of Kearns-Sayre syndrome.^234^

The syndrome is predominantly caused by spontaneous single large-scale deletions of mtDNA,^323,324^ with the 4977 bp deletion being the most prevalent.^325^ Symptom manifestation requires the accumulation of mtDNA deletions beyond a pathogenic threshold, implying that the defective mtDNA must retain replication capability. This likely explains why most deletions occur within the long arc between heavy and light strands but preserve the replication sites.^326^ Additionally, during cellular differentiation, mtDNA deletions are preferentially replicated over wild-type mtDNA, potentially due to a kinetic advantage.^327^ These deletions often result in the loss of genes encoding respiratory chain proteins and tRNAs essential for translation, leading to ATP deficiency and multi-tissue dysfunction.^328^ For instance, the common 4977 bp deletion typically disrupts complexes I, III, IV, and mt-tRNA.^323^

Oxidative stress is evident in cells harboring the 4977 bp deletions.^329^ Respiratory chain complex defects may be independent of the deletion sites, with aberrant translation playing a key role in the pathogenesis of single large-scale mtDNA deletions.^289^ Increased oxidative damage and misfolded mitochondrial proteins inhibit both the ubiquitin-proteasome system and the OXPHOS system.^330^ Inhibition of ubiquitin-proteasome leads to decreased amino acid salvage, which triggers eIF2-α phosphorylation and induces mt-ISR. Under conditions of energy deficit and oxidative stress, genes involved in mt-ISR and autophagy are upregulated.^330^ Prolonged mt-ISR activation propagates and maintains mtDNA deletions, exacerbating the disease condition.^331^ Moreover, amino acid depletion, combined with ATP insufficiency, collectively inhibits the mTOR pathway, thereby increasing autophagy.^330^ The protein synthesis inhibition and autophagy increase, reducing mitochondrial contents, could be pathomechanisms of Kearns-Sayre syndrome.^330^

Notably, alterations in tau protein levels are observed in the cerebrospinal fluid of patients with Kearns-Sayre syndrome.^332^ Tau protein plays a role in ROS generation, mitochondrial dynamics, and mPTP opening,^333^ making its impact on Kearns-Sayre syndrome particularly intriguing. Cardiomyocytes in patients with Kearns-Sayre syndrome display increased and enlarged mitochondria.^334^ Arrhythmias in mitochondrial diseases are closely linked to dysfunctional ion channels, transporters, and membrane excitability caused by ATP deficiency, excessive ROS production, and Δψm collapse.^335,336^ Future research should focus on ionic dysregulation mediated by the mitochondrial Ca2^+^ uniporter complex, uncoupling proteins, and mPTP.

Furthermore, nDNA mutations in *RRM2B*, which encodes the ribonucleotide reductase p53R2 subunit, can lead to multiple mtDNA deletions in Kearns-Sayre syndrome through defective ribonucleotide reductase assembly.^337^ This defective assembly disrupts deoxynucleotide provision and the maintenance of dNTP pools.^338^

For patients with heart block, pacemaker implantation or implantable cardioverter defibrillators are recommended.^339^ In cases where heart failure develops, heart transplantation has been employed.^340^ The long-term safety and feasibility of human retinal progenitor cell transplantation for retinitis pigmentosa have been demonstrated.^341^ Gene therapy also holds promise as a potential treatment for retinitis pigmentosa associated with Kearns-Sayre syndrome.^342^

#### Pearson syndrome

Pearson syndrome, a fatal multisystem mitochondrial disorder, was first identified in 1979 by Pearson, who described it as a condition primarily affecting the bone marrow and exocrine pancreas.^343^ This disease is linked to defects in OXPHOS caused by sporadic single large-scale deletions (or rearrangements) of mtDNA.^344,345^ These deletions vary in size and location, ranging from 1.3 to 10 kb, with the size of the deletion potentially serving as a predictor for disease progression.^306,346^ Approximately 40–50% of patients with Pearson syndrome harbor the “common deletion,” which is 4977 bp in length.^345,347^ Sideroblastic anemia is typically the first and most prominent symptom of Pearson syndrome.^348^ Additional symptoms may include intracerebral bleeding, pancreatic exocrine insufficiency, lactic acidosis, and congenital malformations.^348,349^ The prognosis for Pearson syndrome is poor, with an average age of death being 5.44 years in individuals aged 0–15 and 7.41 years in those aged 0–19.^350^ Patients with the 4977 bp deletion have a higher mortality risk.^349^

Single large-scale mtDNA deletions exhibit phenotypic heterogeneity and contribute to a spectrum of diseases, including Pearson syndrome, Kearns-Sayre syndrome, and PEO.^351^ It is hypothesized that the timing of the mtDNA deletion during fetal development influences the clinical phenotype: late-stage deletions may result in PEO,^4^ while earlier-stage deletions may manifest as Kearns-Sayre syndrome or Pearson syndrome, affecting multiple systems.^4^ Interestingly, Pearson syndrome can evolve into Leigh syndrome or Kearns-Sayre syndrome over time,^352,353^ indicating that the phenotype of an mtDNA deletion disorder may change with age and is influenced by the concentration of mtDNA with deletions.^354^ Patients with Pearson syndrome typically have a higher proportion of mtDNA deletions compared to those with Kearns-Sayre syndrome or PEO.^347,355^

Spontaneous hematological recovery is observed in some Pearson syndrome cases, with a decrease in the amount of deleted mtDNA in blood cells corresponding with an improvement in anemia.^356^ This recovery is attributed to the positive selection of hematopoietic stem cells (HSCs), where HSCs with a higher load of deleted mtDNA are hard to survive, while those with a lower load are selected for survival.^347,348,357^ This concept aligns with findings from a mouse model study, which showed a decrease in the mtDNA deletion load with age in affected tissues, such as peripheral blood and liver.^358^ However, the study also indicated that mtDNA deletions may accumulate in muscle and other tissues, potentially leading to the development of Kearns-Sayre syndrome.^358^ This tissue-specific change in mtDNA deletion load partially explains the progression from Pearson syndrome to Kearns-Sayre syndrome.

In addition to ATP deficiency and oxidative stress caused by mtDNA deletions, iron accumulation may play a significant role in Pearson syndrome. The abnormal iron deposit is a feature in patients with Pearson syndrome, while the molecular mechanisms behind abnormal iron metabolism remain unclear.^359^ In a mouse model of large-scale mtDNA deletion, Pearson syndrome-like anemia worsened with the knockout of Drp1, and Drp1 knockout alone also caused anemia.^359^ Drp1 knockout decreases the pathogenic threshold of mtDNA deletion in erythrocytes,^359^ which drives us to think about the role of MQC in this anemia. Loss of Drp1 leads to HSC quiescence, reducing their regenerative potential.^360^ Interestingly, mitochondria with impaired fission ability are retained and accumulate with HSC divisions, potentially preventing unlimited self-renewal of HSCs.^360^ During erythropoiesis, mitochondria exhibit a specific pattern: increased fusion at early stages and heightened fission at later stages, associated with mPTP opening.^361^ Besides, the quiescence of HSCs is partly mediated by the regulation of mitochondrial content and activity.^362^ Thus, mitochondrial content and activity, which are influenced by mitophagy, are closely linked to HSC differentiation.^363^ A low Δψm is a key trigger for mitophagy, and there is a distinct difference in Δψm between quiescent and cycling-primed HSCs, with quiescent HSCs exhibiting low Δψm.^362^ Moreover, HSC quiescence is supported by an abundance of large lysosomes, and maintaining this quiescence requires restrained lysosomal activity.^362^ Interestingly, while suppressed mitophagy may specifically impair terminal erythrocyte maturation without affecting erythroid progenitor differentiation, hyperactivated mitophagy can hinder the differentiation of erythroid lineages.^364^ Thus, the fine regulation of mitophagy appears crucial during HSC differentiation, highlighting the potential importance of MQC in the anemia associated with Pearson syndrome. Further investigation is needed to determine whether and how energy and oxidative stress lead to MQC alterations, impacting anemia in Pearson syndrome.

Despite the possibility of spontaneous recovery from anemia in some patients with Pearson syndrome, others may require transfusions during infancy and early childhood.^347^ Hematopoietic stem cell transplantation is a potential option for those with persistent transfusion dependency or severe neutropenia.^347,365^ Additionally, mitochondrial augmentation therapy—where autologous CD34^+^ hematopoietic cells are augmented with maternally derived healthy mitochondria—has shown promising outcomes for mtDNA deletion syndromes like Pearson syndrome and Kearns-Sayre syndrome. This therapy has been observed to increase mtDNA content and improve aerobic function, suggesting it may be a viable treatment option.^366^

### The primary mitochondrial diseases caused by nDNA mutations

Given that the majority of mitochondrial proteins are encoded by nDNA, mitochondrial diseases resulting from nDNA mutations encompass not only disturbances in OXPHOS but also defects in various structural or functional proteins essential for mtDNA maintenance, mitochondrial function and structure, and mito-nuclear communication. Consequently, the pathogenesis of mitochondrial diseases caused by nDNA mutations is inherently more complex than those arising from mtDNA mutations.

#### Autosomal dominant optic atrophy

Autosomal dominant optic atrophy (ADOA) is marked by progressive bilateral vision loss and color vision deficits, typically manifesting before the age of 20.^231^ The primary histopathological features of ADOA include the degeneration and demyelination of RGCs.^367^ Mutations in the *OPA1* gene, which encodes a dynamin-related GTPase involved in IMM fusion, are the most common cause of ADOA.^197^ In many cases, haploinsufficiency is the primary pathogenic mechanism, as these mutations often result in premature translation termination.^368^ *OPA1* mutations are typically heterozygous, as bi-allelic homozygous mutations, which lead to a complete loss of OPA1 function, are likely embryonically lethal.^369,370^ Although no cases of bi-allelic homozygous *OPA1* mutations have been reported, bi-allelic compound heterozygous mutations have been observed and are associated with ADOA-plus (or Behr syndrome),^371,372^ which presents with additional multisystemic features beyond the optic neuropathy.^372^ Other genes involved in mitochondrial dynamics, such as *OPA3*, *Drp1*, and *Mfn2*, can also cause ADOA or ADOA-plus.^197^

The specific vulnerability of RGCs to *OPA1* mutations remains unclear. However, research has shown that *OPA1* mutations lead to defective differentiation and impaired mitochondrial function in RGCs, as demonstrated in human retinal organoids.^373^ The distinct mitochondrial morphology observed in RGCs and optic nerves in mouse models may provide some insights. In the unmyelinated segments of RGC axons, mitochondria are typically round before the lamina cribrosa, whereas they become elongated after crossing this structure in the myelinated segments, suggesting dynamic changes during mitochondrial transport.^197^ Elongated mitochondria are generally associated with enhanced ATP production, decreased fission, or increased fusion.^374^ It is hypothesized that *OPA1* mutations may impair mitochondrial fusion after crossing the lamina cribrosa, leading to mitochondrial fragmentation. The clustering of fragmented mitochondria could cause traffic jams, obstructing the axonal transport of mitochondria.^375^ This theory aligns with observations that small RGCs with thin axons, which have limited mitochondrial transport capacity, are the first to be lost in ADOA.^197^ This suggests that disrupted mitochondrial transport may contribute to the RGC-specific susceptibility to *OPA1* mutations. OPA1 also plays a role in intercellular mitochondria transfer.^187^ A significant proportion of mitochondria in RGC axons are not degraded by lysosomes within the RGC soma but are instead transferred to astrocytes at the optic nerve head for transcellular degradation.^376^ The potential impairment of mitochondrial transfer and transport due to *OPA1* mutations should be considered as a contributing factor to the vulnerability of RGCs. Further research is needed to verify whether these mitochondrial properties specifically facilitate the susceptibility of RGCs to *OPA1* mutations.

The *OPA1* mutation has been shown to increase Drp1 expression, thereby promoting mitochondrial fission. Inhibiting Drp1 can help balance mitophagy and improve mitochondrial abnormalities associated with ADOA.^377^ Mitophagy, which can be activated by AMPK, is another critical factor; inhibiting both AMPK and mitophagy has been found to preserve mitochondrial content in RGC axons and mitigate visual loss caused by *OPA1* mutations.^378^ Additionally, *OPA1* mutations can lead to an increase in PINK1-Parkin-independent mitophagy, which may be directly driven by the excessive presence of fragmented mitochondria.^375^ This overactivation of mitochondrial fission and mitophagy could be central to the pathogenesis of ADOA caused by *OPA1* mutations. Chronic inhibition of mitochondrial fusion due to OPA1 loss results in mtDNA depletion, exacerbating mitochondrial dysfunction,^379^ and excessive mitophagy further contributes to this depletion.^375^ The haploinsufficient *OPA1* mutations not only impact mtDNA maintenance but also downregulate nuclear genes encoding mitochondrial components, implying the potential role of mito-nuclear communication in ADOA.^373^ Given OPA1’s role in mediating intercellular mitochondrial transfer *via* MDVs,^187^ the ability to secrete damaged mitochondria into the extracellular space might be impaired in ADOA. The dysfunctional MQC resulting from OPA1 loss reduces mitochondrial content and leads to secondary mtDNA depletion, disrupting OXPHOS.

Beyond its influence on mitochondrial fusion, OPA1 also plays a key role in preventing remodeling of cristae structure and mobilization of cytochrome c during apoptosis.^380,381^ Loss of OPA1 disturbs the structure and integrity of the IMM, leading to the release of cytochrome c in a which is mainly sequestered within the tight cristae junctions.^380–382^ Cytochrome c release is a well-known trigger of apoptosis. Excessively fragmented mitochondria can induce mtROS overproduction,^144^ making oxidative stress correlated with ADOA.^383,384^ The secondary mtDNA depletion may exacerbate OXPHOS disturbance, increasing the production of mtROS. Consequently, *OPA1* mutations might facilitate stress-induced cytochrome c release by promoting the remodeling of cristae and mobilization of cytochrome c, thereby leading to RGCs degeneration.^380,381,383,385^

Given the critical role of mitochondrial cristae architecture in preventing mtDNA release and inflammation,^299^ mitochondrial inflammation in ADOA warrants significant attention. OPA1 loss has been associated with muscle inflammation due to mitochondrial dysfunction,^386^ even in the absence of mtDNA leakage into the cytosol. In this context, OPA1 deficiency induces NF-κB-mediated inflammation through the TLR9 pathway.^386^ Additionally, *OPA1* defects reduce muscle mass and lead to premature death.^379^ The stress-induced mt-ISR, triggered by OPA1 loss, also contributes to muscle loss, and the reduction of FGF21 (a downstream hormone of mt-ISR) can mitigate this muscle wasting.^379^ Interestingly, while *OPA1* loss impairs mitophagy in muscle cells,^386^ it increases mitophagy in RGCs. Understanding these tissue-specific differences in response to *OPA1* mutations could help explain the phenotypic heterogeneity and tissue-specificity observed in ADOA.

#### Alpers-Huttenlocher syndrome

Alpers-Huttenlocher syndrome is the most prevalent mitochondrial disease caused by nDNA mutations. It is an autosomal recessive hepatocerebral syndrome with early onset,^387^ typically presenting with a triad of intractable seizures, developmental regression, and liver dysfunction.^388^ Notably, hepatic dysfunction can be aggravated by exposure to valproate, sometimes leading to a misdiagnosis of valproate hepatotoxicity.^389^ The disease is primarily caused by mutations in the *POLG* gene, such as p.A467T, p.W748S, and p.G848S, which result in mtDNA depletion.^387,390^ Alpers-Huttenlocher syndrome is likely the most common *POLG*-related disorder.^391^ Recessive *POLG* mutations can be homozygous or compound heterozygous, with compound heterozygous mutations in trans often associated with a more severe and earlier-onset phenotype, whereas homozygous recessive mutations tend to result in a milder and later-onset presentation.^392^ Additionally, ecogenetic structural nucleotide variants can influence the clinical phenotype.^393^ It’s worth noting that compound heterozygous mutations in *C10orf2*, which encodes the mitochondrial replicative helicase Twinkle, can also cause mtDNA depletion and present as Alpers-Huttenlocher syndrome.^394^ Mutations in *C10orf2* disrupt mtDNA maintenance, leading to secondary mtDNA depletion and subsequent mitochondrial dysfunction.^309^

Another *POLG*-related disorder, Myocerebrohepatopathy spectrum (MCHS), presents with a triad of hypotonia, developmental delay, and hepatopathy.^395^ Other clinical manifestations may include renal tubulopathy, choreoathetosis, neuropathy, ataxia, and cataracts.^2,396^ MCHS is the earliest *POLG*-related disorder with mtDNA depletion, with a median onset age of 4.7 months.^391,396^ A case report documented the clinical progression of a child from infantile MCHS to Alpers-Huttenlocher syndrome, suggesting that MCHS and Alpers-Huttenlocher syndrome may represent different stages or severities of the same disorder.^395^

The histopathological features of mitochondrial encephalopathies include astrocyte activation, cortical degeneration, and neuron loss.^397^ In iPSC-derived neural stem cells with compound heterozygous *POLG* mutations, BNIP3-mediated mitophagy is increased, likely due to elevated ROS levels and a low NAD^+^/NADH ratio. Concurrently, decreased SIRT1 signaling and increased UCP2 signaling contribute to neuronal senescence.^398^ The combination of mitochondrial dysfunction and senescence leads to neuron loss in *POLG*-related disorders.^398^ Astrocytes, which play a pivotal role in supporting neurons,^399^ also suffer from dysfunction due to mtDNA defects, contributing to the development of mitochondrial encephalopathy. Indeed, astrocyte dysfunction has been demonstrated in patients with Alpers-Huttenlocher syndrome,^400^ and astrocytic neurotoxicity caused by mitochondrial dysfunction associated with *POLG* mutations also has been observed.^401^ The failure of mtDNA maintenance due to *POLG* mutations results in the loss of complexes I and IV in astrocytes,^402^ impairing their ability to proliferate and respond effectively to neuronal damage.^403^ *POLG* mutations can also disrupt mitochondrial biogenesis, as well as mitophagy, leading to astrocytic neurotoxicity and a loss of supportive functions.^402^ Similarly, mtDNA depletion caused by *C10orf2* mutations leads to chronic astrocyte activation and dysfunction, which may produce neurotoxic factors and promote neuronal morphology changes and progressive spongiotic encephalopathy.^404^

Valproic acid is contraindicated in Alpers-Huttenlocher syndrome due to its hepatotoxicity, which is linked to increased apoptotic sensitivity through mPTP opening.^405^ In iPSC-derived hepatocytes from patients with Alpers-Huttenlocher syndrome, mitochondria exhibit reduced mtDNA content, cristae disorganization, and OXPHOS disturbances.^405^ However, the full extent of functional changes in hepatocytes remains unknown.

Currently, there are no effective treatments for Alpers-Huttenlocher syndrome. Symptomatic treatments, such as anti-epileptic therapy (excluding valproic acid and divalproex) and ventilation support, should be considered.^393^ Due to the multisystem involvement, liver transplantation alone is contraindicated.^393^ Supportive therapies, such as a ketogenic diet, may provide some benefit.^406^

#### Ataxia neuropathy spectrum

Ataxia Neuropathy Spectrum encompasses a group of mitochondrial disorders, including sensory ataxia, neuropathy, dysarthria, and ophthalmoplegia (SANDO), as well as mitochondrial recessive ataxia syndrome (MIRAS). SANDO was first identified as a syndrome involving multiple mtDNA deletions in muscle and peripheral nerve tissues by *Fadic* et al. in 1997.^407^ It is primarily caused by recessive *POLG* mutations and is considered part of the PEO-plus syndrome spectrum.^408,409^ While SANDO is most commonly associated with multiple mtDNA deletions due to *POLG* mutations, it can also be caused by mutations in the *C10orf2* and *RNASEH1* genes.^410–413^ RNase H1, which cleaves the RNA component of RNA: RNA hybrids as an endonuclease,^414^ plays a critical role not only in nDNA replication but also in mtDNA replication.^415^ In mitochondria, RNase H1 is essential for directing RNA primer formation for origin-specific initiation of mtDNA replication and for removing primers at the origin of replication to complete mtDNA replication.^416,417^ The absence of RNase H1 activity leads to defective mtDNA replication, resulting in linear deletions and depletion of mtDNA.^418^

MIRAS, an adult-onset mitochondrial disease, is characterized by clinical manifestations including ataxia, headache, axonal neuropathy, late-onset ophthalmoplegia, partial epilepsy in the occipital lobe, and a high risk of status epilepticus.^410^ Mutations in the *POLG* gene, such as W748S and A467T, have been identified in patients with MIRAS exhibiting multiple mtDNA deletions.^410,419–421^ Recent research has shown that *POLG* also plays a role in antiviral defense, and mutations in this gene can compromise antiviral tolerance, leading to epilepsy and liver disease in MIRAS and other *POLG*-related disorders.^422^

The spectrum of *POLG*-related disorders includes conditions such as myoclonic epilepsy myopathy sensory ataxia (MEMSA, characterized by epilepsy, myopathy, and ataxia without ophthalmoplegia), MCHS, Alpers-Huttenlocher syndrome, SANDO, MIRAS, and PEO.^423^ Despite being linked by common *POLG* mutations, these disorders differ in their onset age and the specific mtDNA defects they involve, leading to distinct clinical presentations.^423^ For example, MCHS typically manifests earliest, in neonates or infants, Alpers-Huttenlocher syndrome appears in infancy or childhood, and the remaining disorders are more likely to present in adolescence or adulthood.^396^ Early-onset and juvenile-onset *POLG*-related disorders generally result from biallelic pathogenic variants with autosomal recessive inheritance, whereas late-onset disorders (mainly PEO) may arise from a heterozygous *POLG* pathogenic variant with autosomal dominant inheritance.^424^

Another mitochondrial ataxia, infantile-onset spinocerebellar ataxia (IOSCA), is an autosomal recessive disorder characterized by sensory axonal neuropathy and progressive atrophy of the cerebellum, brain stem, and spinal cord.^425–427^ IOSCA is caused by two point mutations in the *C10orf2* gene, which encodes the mitochondrial helicase Twinkle, as well as a rarer splice variant known as Twinky.^428^ These mutations result in a preponderance of messenger RNAs encoding Y508C polypeptides, leading to a Y508C alteration in the helicase domain of Twinkle or Twinky.^428,429^ IOSCA is classified as a mtDNA depletion syndrome, as patients’ brains show significant mtDNA depletion without mtDNA deletions or an increased number of mtDNA point mutations.^429^

The pathogenesis of *POLG*-related disorders has been discussed previously, but it’s important to note that different mutant genes involved in mtDNA replication may lead to distinct patterns of mtDNA deletions, varying in size and location. These variations can affect mitochondrial function differently depending on the nature of the mutations.^313^ Mitochondrial dysfunction in cerebellar tissue, mediated by impaired mitophagy, has been confirmed as a key factor in the development of ataxic diseases.^430^ Further research is needed to elucidate the molecular and functional changes in mitochondria that underlie mitochondrial ataxias.

As with most mitochondrial diseases, there are no specific treatments or cures for MIRAS, SANDO, IOSCA, and MEMSA. Treatment is primarily supportive and symptomatic, with clinical management focused on alleviating symptoms and improving quality of life.

#### Barth syndrome

Barth syndrome, an X-linked mitochondrial disorder, is characterized by a clinical triad of cardiomyopathy, skeletal myopathy, and neutropenia, along with aberrant cristae morphology and respiratory chain abnormalities.^431^ The gene responsible for Barth syndrome, *TAZ* (also known as *G4.5*), was identified as an X-linked gene encoding an acyl-specific phospholipid transacylase involved in remodeling cardiolipin acyl chains within mitochondrial membranes.^432,433^ In highly oxidative tissues such as the heart and skeletal muscle, tetralinoleoyl cardiolipin is the predominant form.^434,435^ Mutations in *TAZ* lead to a deficiency of tetralinoleoyl cardiolipin and an accumulation of monolysocardiolipins, which lack a linoleoyl acyl group.^436,437^ The elevated ratio of monolysocardiolipins to tetralinoleoyl-cardiolipin represents not only a biochemical hallmark but also the underlying molecular mechanism of Barth syndrome.^438^ This cardiolipin imbalance disrupts the phospholipid composition of the IMM, compromising the function of respiratory chain complexes and other mitochondrial proteins.^439^ Cardiolipin is essential for the stability of respiratory chain supercomplexes, which are crucial for the efficient operation of OXPHOS.^88^ In Barth syndrome, *TAZ* mutations disrupt supercomplex formation, resulting in reduced ATP production.^440^ Moreover, cardiolipin imbalances may interfere with the coupling of respiration to ATP synthesis, further diminishing energy production.^439^ The loss of respiratory chain supercomplexes also contributes to increased ROS, a phenomenon observed in Barth syndrome.^441^ Cardiolipin also plays a pivotal role in mitophagy, acting as a mediator in the process.^442^ Defective cardiolipin remodeling due to *TAZ* mutations hinders the initiation of mitophagy, leading to dysfunctional OXPHOS and heightened oxidative stress.^443^ Restoration of mitophagy through mTOR complex 1 inhibition has been shown to alleviate cardiomyopathy in Barth syndrome.^444^ The failure of dysfunctional mitophagy to remove and recycle mitochondria with defective components may exacerbate mitochondrial stress.

Current treatment strategies for Barth syndrome focus on managing heart failure, cardiac arrhythmias, and neutropenia.^445^ Physical therapy and nutritional support are also important considerations. Beyond gene or cell therapy, lipid replacement therapy aimed at restoring mitochondrial cardiolipin levels presents a promising therapeutic approach.^445^

#### Friedreich’s ataxia

Friedreich’s ataxia is the most common spinocerebellar ataxia with autosomal recessive inheritance.^446^ The disease is classically characterized by progressive and unremitting ataxia of the limbs and trunk, typically presenting before the age of 25.^447^ Additional symptoms include dysarthria, absent tendon reflexes in the lower extremities, loss of deep sensation, scoliosis, and cardiomyopathy.^447^ This condition is most commonly associated with a homozygous unstable guanine-adenine-adenine (GAA) trinucleotide expansion in the first intron of the *Frataxin* gene (X25) on chromosome.^446,448^ The age of onset is correlated with the number of GAA repeats.^449^ Some cases also result from a compound heterozygous expansion combined with a point mutation or deletion.^450,451^ Atypical phenotypes of Friedreich’s ataxia have also been reported.^450^ The GAA expansion within the intron silences the *Frataxin* gene, leading to reduced production of Frataxin protein and the associated disease phenotypes.^452^ The silencing mechanism may involve the formation of sticky DNA (a novel DNA structure) and epigenetic modifications.^452,453^

Frataxin is a mitochondrial protein essential for maintaining mitochondrial iron homeostasis.^454^ Its critical role in the assembly or transport of iron-sulfur (Fe-S) clusters means that Frataxin deficiency leads to aconitase and mitochondrial Fe-S respiratory enzyme deficiencies (respiratory chain complexes I, II, and III), resulting in mitochondrial iron accumulation.^455–457^ The Fe-S clusters mediate electron transfer and ROS production in complexes I, II, and III.^458^ Specifically, in response to Frataxin deficiency, the activation of iron-responsive element binding protein 1 (Fe-S protein) increases cellular iron uptake, with the iron being translocated into mitochondria *via* mitochondrial iron transporters in an attempt to compensate for impaired Fe-S cluster biogenesis.^459^ However, due to the *Frataxin* defect, mitochondrial iron cannot be effectively utilized, leading to iron accumulation and oxidation, which in turn causes severe oxidative stress and subsequent cell death.^459,460^

The development of Friedreich’s ataxia within the nervous system is believed to be driven by oxidative stress, iron neurotoxicity, and neuroinflammation.^461^ In Drosophila models, reduced *Frataxin* expression impairs mitochondrial transport in neuronal regions, potentially affecting axonal function.^462^ Other MQC processes appear robust, with increased mitochondrial turnover and dynamics observed in the hearts of *Frataxin*-knockout mouse models, suggesting an effort to re-establish mitochondrial energetic and redox homeostasis.^463^ The interplay of oxidative stress and disordered iron metabolism raises questions about the role of ferroptosis, a form of cell death linked to iron-dependent lipid peroxidation, in Friedreich’s ataxia. While ferroptosis is indeed implicated in this disease,^464,465^ the precise contribution of mitochondrial dysfunction to ferroptosis in Friedreich’s ataxia remains unclear.

#### Fatty acid oxidation disorder

Fatty acid oxidation disorder represents a spectrum of syndromes primarily caused by defects in β-oxidation, inherited in an autosomal recessive manner.^466^ Mutations in specific genes result in defective enzymes, such as acylcarnitine translocase, carnitine palmitoyltransferase, and medium-chain acyl-CoA dehydrogenase.^467^ Among these, medium-chain acyl-CoA dehydrogenase deficiency, due to mutations in the *ACADM* gene, is the most prevalent form of fatty acid oxidation disorder.^468^ Clinically, the disorder is characterized by metabolic symptoms, including non-ketotic hypoglycemia, vomiting, encephalopathy, and acidosis, as well as muscular symptoms such as rhabdomyolysis and exercise intolerance.^466^ The critical role of β-oxidation in energy production means that its failure results in an ATP deficit. Beyond energy stress, mitochondrial dysfunction—driven by the lipotoxicity of accumulating fatty acids and carnitine derivatives—plays a central role in disease progression.^467,469^ Those toxic lipids can lead to respiratory chain complex inhibition, OXPHOS uncoupling, ROS overproduction, and persistent mPTP opening, resulting in ultimate cell death.^467,469^

#### Leigh syndrome

Leigh syndrome, also known as subacute necrotizing encephalopathy, is the most common mitochondrial disorder in childhood. It typically manifests before the age of 2 years and presents with a range of symptoms including hypotonia, epilepsy, respiratory distress, neurodevelopmental delay, ataxia, ophthalmological abnormalities, and lactic acidosis.^470^ Over 75 genes in both nDNA and mtDNA have been identified as causes of this condition,^471^ most of which are involved in OXPHOS or other energy production processes.^470^

Complex I deficiency is the leading cause of Leigh syndrome,^472^ with mutations in the nuclear gene *NDUFS4* being the most common cause of complex I-associated Leigh syndrome. These mutations directly impair complex I function.^470^ Recent research has shown that *NDUFS4* mutations also disrupt direct neuronal reprogramming of proliferating astrocytes through mechanisms involving endoplasmic reticulum stress, mt-UPR, and mt-ISR.^473^ Besides *NDUFS4*, other nuclear genes contribute to Leigh syndrome by causing deficiencies in assembly factors, cofactors, or biosynthetic pathways essential for energy metabolism.^474^ For instance, pyruvate dehydrogenase complex deficiency, often caused by mutations in *PDHA1* (which encodes the E1 alpha subunit of pyruvate dehydrogenase),^475,476^ is another significant contributor to the disease.^477^ This deficiency disrupts the TCA cycle by depleting substrates needed for OXPHOS, leading to a collapse of energy production. Mutations in nDNA that cause mtDNA depletion are also implicated in Leigh syndrome, particularly those affecting the *SUCLA2* or *SUCLG1* genes, which encode subunits of succinyl-CoA ligase.^470^ Defective succinyl-CoA ligase not only hinders the TCA cycle by blocking the conversion of succinyl-CoA but also interferes with mitochondrial nucleotide salvage pathways by disrupting interactions with mitochondrial nucleotide diphosphate kinase, resulting in both metabolic dysfunction and mtDNA depletion.^478,479^ Primary mtDNA mutations can also lead to Leigh syndrome, with mutations in *MT-ND5* (encoding a complex I subunit) and *MT-ATP6* (encoding a complex V subunit) being common culprits.^476,480^ These mutations universally cause a breakdown in mitochondrial energy metabolism, leading to energy failure. MQC mechanisms are also implicated in the pathogenesis of Leigh syndrome-related gene mutations.^143,473,481–485^

Notably, although hypoxia might reduce energy production of OXPHOS, it alleviates Leigh syndrome in disease models by reducing mtROS generation and activating hypoxia-inducible factor pathway,^12^ indicating the downstream pathways of genetic mitochondrial dysfunction are promising therapeutic targets.

#### Mitochondrial neurogastrointestinal encephalomyopathy

Mitochondrial neurogastrointestinal encephalomyopathy (MNGIE), an autosomal recessive disorder first identified in 1978,^486^ is characterized by a range of clinical symptoms including cachexia, gastrointestinal dysmotility, peripheral neuropathy, ophthalmoparesis, and leukoencephalopathy.^487^ Mutations in the *TYMP* gene, which result in the loss of thymidine phosphorylase (TP) activity, are the primary cause of MNGIE.^39^ TP is responsible for catalyzing the reversible phosphorylation of deoxythymidine and deoxyuridine into thymine, uracil, and 2-deoxyribose 1-phosphate.^39,488^ The deficiency in TP activity leads to the accumulation of deoxythymidine and deoxyuridine, which disrupts the balance of mitochondrial nucleotide pools, thereby impairing mtDNA replication.^489^ This imbalance results in the accumulation of point mutations, multiple deletions, and depletion of mtDNA,^490,491^ ultimately leading to a failure in OXPHOS. The defective TP caused by *TYMP* mutations leads to the excessive accumulation of nucleosides in both mitochondria and lysosomes.^492^ The buildup of nucleosides, which possess weak alkaline properties, can alter the acidic environment of lysosomes, potentially suppressing lysosomal activity.^492^ Consequently, defective mitochondria are not adequately degraded and recycled, undermining the mitochondria’s ability to cope with stress, which may exacerbate mitochondrial dysfunction.

In addition to *TYMP* mutations, MNGIE-like phenotypes can also arise from mutations in other genes. For example, mutations in *RRM2B* can cause MNGIE-like symptoms by disrupting the docking interface of the ribonucleoside reductase small subunit homodimer, thereby impairing ribonucleoside reductase activity and damaging the mitochondrial nucleotide pool, which leads to mtDNA depletion.^493^ Mutations in *LIG3*, the only mtDNA ligase essential for mtDNA replication and repair, also affect mtDNA maintenance and have been reported in patients with MNGIE, leading to mtDNA depletion.^494^

Current treatment options for MNGIE include hemodialysis and peritoneal dialysis, enzyme replacement therapy, orthotopic liver transplantation, hematopoietic stem cell transplantation (HSCT), celiac plexus neurolysis, and splenic nerve blockage.^495^ However, while HSCT can restore biochemical homeostasis, it often fails to alleviate gastrointestinal symptoms.^496^ Encouragingly, gene therapy has shown efficacy in MNGIE murine models,^497,498^ and its potential effectiveness in human patients is highly anticipated.

#### Myopathy, lactic acidosis and sideroblastic anemia

Myopathy, lactic acidosis, and sideroblastic anemia (MLASA) is an autosomal recessive mitochondrial disorder affecting skeletal muscle and bone marrow, characterized by mitochondrial myopathy, lactic acidosis, and sideroblastic anemia.^499,500^ The condition is primarily caused by mutations in the *PUS1* gene, which encodes pseudouridine synthase 1.^500^ *PUS1* mutations result in a defective PUS1p, the catalytic center of pseudouridine synthase 1, which is responsible for pseudouridylating mt-tRNAs.^500^ In patients with MLASA, the loss of pseudouridylation at tRNA sites typically modified by PUS1p has been observed.^501^ Recent research using MLASA patient-specific iPSCs and mouse models has shown that PUS1p defects lead to a reduction in mt-tRNA due to the loss of pseudouridylation, which subsequently causes abnormal mitochondrial translation.^502,503^ Moreover, the pseudouridylation of mt-tRNA-derived fragments is also affected by *PUS1* mutations, further contributing to defective mitochondrial protein synthesis.^504^ This mitochondrial dysfunction, stemming from abnormal mitochondrial proteins, disrupts erythropoiesis, leading to anemia.^502,503^ Another mutation associated with MLASA involves the *YARS2* gene, which encodes mitochondrial tyrosyl-tRNA synthetase, an enzyme that catalyzes the covalent linkage of tyrosine to its corresponding tRNA.^505^ Mutations in *YARS2* reduce the aminoacylation activity of this enzyme, leading to faulty translation of OXPHOS subunits, particularly complexes I and IV.^506^ Both *PUS1* and *YARS2* mutations ultimately result in the collapse of OXPHOS.

#### Sengers syndrome

Sengers syndrome is another autosomal recessive mitochondrial disease, characterized by hypertrophic cardiomyopathy, myopathy, lactic acidosis, and congenital cataracts.^507^ Mutations in the *AGK* gene, located in nDNA, have been identified as the cause of this syndrome.^508^ The *AGK* gene encodes mitochondrial acylglycerol kinase (AGK), a multi-substrate lipid kinase that phosphorylates monoacylglycerol and diacylglycerol to produce lysophosphatidic acid and phosphatidic acid, thus playing a role in phospholipid synthesis and various signaling pathways.^508^ AGK is also involved in the synthesis of IMM-specific cardiolipin.^509^ Beyond lipid metabolism, AGK acts as a subunit of the TIM22 complex, promoting the import of mitochondrial carrier proteins independently of its kinase activity.^510,511^ AGK is also thought to interact with complex I of the respiratory chain, a function that appears to be disrupted by *AGK* deficiency rather than mutation, leading to complex I dysfunction.^512^ This interaction requires further validation. The pathogenesis of Sengers syndrome likely involves disruptions in mitochondrial membrane phospholipid metabolism and the protein import machinery, with further studies needed to elucidate the detailed molecular mechanisms.

#### Perrault Syndrome

Perrault syndrome is an autosomal recessive disorder characterized by sensorineural hearing loss and ovarian dysgenesis, though other neurological symptoms may also be present.^513^ The syndrome is associated with mutations in several genes, including *CLPP, ERAL1, HARS2, LARS2*, and *C10orf2*.^514^ The CLPP protein is an endopeptidase component of a mitochondrial ATP-dependent proteolytic complex involved in the degradation of defective proteins through mt-UPR.^515^ The functional CLPP tetradecamer interacts with the hexameric caseinolytic peptidase X to proteolyze specific protein substrates.^516^ Mutations in *CLPP* that reduce its proteolytic activity or disrupt its interaction with caseinolytic peptidase X lead to a breakdown in mitochondrial protein quality control, resulting in mitochondrial dysfunction.^517^ CLPP plays a critical role in the turnover of complex I, providing protection against mtROS overproduction and related damage.^518^ This protective function of CLPP may serve as an important supplementary mechanism to mitophagy in clearing ROS-damaged mitochondria.^519^ However, the role of CLPP in mt-UPR in mammals is debated.^520^ Some studies suggest that CLPP loss may even alleviate mitochondrial diseases caused by defective DARS2, the mitochondrial aspartyl tRNA synthase.^520,521^ CLPP also participates in other aspects of mitochondrial metabolism, though its precise role in Perrault syndrome remains unclear.^522^

*HARS2* and *LARS2* encode mitochondrial histidyl tRNA synthetase and leucyl tRNA synthetase, respectively, which are responsible for catalyzing the covalent attachment of histidine and leucine to their corresponding tRNAs. The activity of these aminoacyl tRNA synthetases is essential for mtDNA translation.^523,524^ Mutations in *HARS2* or *LARS2* reduce the aminoacylation activity of these tRNA synthetases, resulting in impaired mitochondrial translation and subsequent mitochondrial dysfunction.^523,524^ ERAL1 binds to mitochondrial 12S rRNA as a chaperone and is essential for the assembly of the small 28S subunit of the mitochondrial ribosome.^525^ Mutations in *ERAL1* impair RNA processing and mitochondrial translation.^526^ In other words, the collapse of mitochondrial protein homeostasis appears to be the predominant mechanism in Perrault syndrome.^514^

Symptomatic treatments for Perrault syndrome include cochlear implantation for hearing loss and estrogen replacement therapy. For women desiring pregnancy, options such as in vitro fertilization and oocyte cryopreservation may be considered.^514^

### Age-related diseases

Beyond mitochondrial diseases directly caused by hereditary mtDNA and nDNA mutations, aging significantly contributes to the gradual decline in mitochondrial function and the efficiency of oxidative phosphorylation, resulting in decreased ATP production and elevated ROS generation.^527^ This mitochondrial dysfunction is intricately linked to the onset and progression of various age-related diseases, particularly neurodegenerative disorders such as Alzheimer’s disease (AD) and Parkinson’s disease (PD).^528,529^ Pathogenic factors, including mitochondrial genome defects, increased oxidative stress, disrupted MQC, impaired mitochondrial proteostasis, and neuroinflammation, are central to the development and progression of these neurodegenerative disorders.^529,530^ Targeted mitochondrial therapies show promise as potential treatments for these conditions.^529^ Furthermore, MDPs and mtDNA single nucleotide polymorphism within coding regions are strongly associated with age-related diseases.^78,531^ For example, humanin has been found to prevent synaptic loss and reduce inflammation, offering therapeutic potential in AD.^78^ Similarly, SHLP2 and its variants can mitigate mitochondrial dysfunction and protect dopaminergic neurons, thereby lowering the risk of PD.^531^ Additionally, dysregulation of mitochondrial microRNAs is implicated in mitochondrial dysfunction and is associated with neurodegenerative diseases.^532^

Damage to mitochondrial antioxidant enzymes, combined with excessive ROS, triggers oxidative stress that leads to mtDNA point mutations and deletions accumulating over time. These genetic alterations disturb MQC, and ultimately result in energy depletion, oxidative damage, and apoptosis.^527,529,532^ Moreover, ROS accelerates the accumulation of oxidative byproducts through mitochondrial proteases and the mt-UPR.^527^ Released mtDNA, fragmented mitochondria, and other substances from dying neurons can initiate inflammatory responses, further driving the progression of neurodegenerative diseases.^209^ Elevated ROS levels, along with mtDNA polymerase mutations that cause replication errors, contribute to mtDNA mutations, multiple deletions, and reduced copy numbers, all of which accumulate with age, leading to mitochondrial dysfunction and cell death.^209,530^ Notably, AD is associated with mtDNA mutations and reduced copy numbers, while PD is closely linked to mtDNA deletions.^530,533^

In AD, neuronal damage and dysfunction are closely associated with increased oxidative stress due to amyloid-β accumulation and tau aggregation, as well as impaired mitochondrial bioenergetics and MQC networks.^532,533^ Mitochondrial and autophagic dysfunctions also contribute to microglial activation and neuroinflammation, further advancing AD pathogenesis.^534^ Similarly, PD is characterized by increased oxidative stress, abnormal mitochondrial dynamics, impaired biogenesis, and autophagy defects.^535,536^ These dysfunctions are primarily associated with mutations in the genes of certain proteins, such as α-synuclein, Parkin, and PINK1.^3,529^ Accumulation of α-synuclein in mitochondria can lead to the formation of oligomers that interact with mitochondrial membranes, inhibiting complex I activity and causing excessive mtROS production, which induces neuronal apoptosis.^3,529^ Mutations in PINK1 or Parkin disrupt PINK1-Parkin-dependent mitophagy, leading to the accumulation of defective mitochondria.^533,536^ Moreover, PD-related neurotoxins and mutations can induce mitochondrial fission, exacerbating neuroinflammation.^537^ The examples of AD and PD illustrate the multifaceted pathological roles that mitochondria play in age-related diseases.

In addition to genetic factors and aging, several other elements contribute to the progression of neurodegenerative diseases, including lifestyle choices and environmental exposures. Genomic instability, telomere attrition, and epigenetic modifications may all increase disease susceptibility.^528^ Moreover, factors such as dysregulated nutrient sensing, stem cell exhaustion, altered intercellular communication, and immune dysfunction also play significant roles in the development of these diseases.^528^ Future research should focus on further exploring these factors and their interactions to enhance understanding of the underlying pathogenic mechanisms.

It is evident that age-related diseases are intricately linked to mitochondrial dysfunction associated with aging. Their pathogenesis typically results from the long-term accumulation of factors such as pathological protein aggregation and disrupted MQC. In contrast, inherited mitochondrial diseases arise from specific mutations in mtDNA or nDNA that directly impair mitochondrial function, often leading to significant clinical symptoms in infancy or adolescence. Although both types of diseases share mitochondrial dysfunction as a common feature, they differ in their pathogenesis, age of onset, and clinical phenotypes.

Overall, mitochondrial diseases are increasingly recognized as pathway-based disorders rather than merely energy-related conditions.^7^ Numerous biological processes, such as autophagy, are energy-dependent, and severe energy stress can suppress these processes.^538^ Consequently, a genetic deficit in ATP can induce cellular dysfunction. ATP deficit can also serve as a signal to trigger downstream pathways such as AMPK activation, resulting in alterations of mitochondrial function.^330^ The pathological consequences of the downstream pathways activated by ATP shortage may be of importance for disease development, despite the currently incomplete understanding. As previously discussed, the overproduction of mtROS plays a critical signaling role in triggering MQC, apoptosis, and inflammation. Moreover, elevated mtROS levels can exacerbate the maintenance of mtDNA.^539^ Oxidative stress may be a common contributing factor in mitochondrial diseases. Additionally, the maintenance of a normal Δψm is essential for ATP synthesis, mitochondrial protein and ion transport, and mito-nuclear communication signaling.^83^ For example, reduction in Δψm disturbs mitochondrial calcium homeostasis, which may be a pathomechanism in mitochondrial diseases.^11^ Certain MQC processes, such as IMM fusion, also rely on a healthy Δψm.^300^ A reduction in Δψm is a key initiator of mt-ISR and mitophagy; however, chronic activation of mt-ISR or mitophagy may accelerate disease progression.^540^ Thus, the collapse of Δψm can further deteriorate cellular functions. Changes in MQC are observed in mitochondrial diseases. Although these processes are intended to help affected cells resist stress, pathological and chronic activation of them can aggravate mitochondrial dysfunction and promote disease progression.^125,248^ Despite the significant attention given to MQC in mitochondrial diseases, the underlying molecular mechanisms behind these changes remain largely unknown. The roles of mitochondrial apoptosis and inflammation in these diseases are also not fully understood now. Oxidative stress has been emphasized in the fields.^10^ Given their established influence in age-related diseases, the potential contributions of mitochondrial apoptosis and inflammation to primary mitochondrial diseases warrant close investigation.

Under OXPHOS defects or mitochondrial stress, the mito-nuclear communication pathways like mt-ISR will be activated. These activations could be significant in those mitochondrial diseases caused by mutations in mt-tRNAs due to defects of mitochondrial protein synthesis.^9^ Notably, research by *Burr* et al. has demonstrated cell lineage-specific mitochondrial resilience to mutations during mammalian organ development.^541^ The specific modes of mito-nuclear communication in response to genetic mitochondrial dysfunction, which determine tissue-specific vulnerability to mtDNA defects, are independent of mt-UPR and mt-ISR.^541^ Different triggers and degrees of mt-ISR activation in response to identical mutational loads in various cell types may also contribute to this specificity,^540^ potentially in the later phases.^541^ A deeper exploration of mito-nuclear communication is essential for understanding the pathogenesis and heterogeneity underlying mitochondrial diseases. In addition to the roles of mt-UPR and mt-ISR in facilitating mito-nuclear communication to manage cellular stress,^542^ the role of epigenetics, an integral component of mito-nuclear communication, is gaining increasing attention.^543^ Future research should investigate the pathological role of epigenetic modifications, such as lactylation, particularly given that elevated lactate levels are a common characteristic of mitochondrial diseases.^544^

While various phenotypic syndromes and their associated gene mutations have been discussed, the genetic and phenotypic heterogeneity of mitochondrial diseases is notably complex. The relationship between common pathogenic variants and the phenotypic syndromes of mitochondrial diseases is summarized in Table 2.

**Table 2:** The common mutant genes of mitochondrial phenotypic syndrome

Phenotype	Mutant gene	Protein	Function/role	Reference
Alpers-Huttenlocher syndrome	POLG	MtDNA polymerase gamma	MtDNA replication maintenance	^390,392^
ADOA	OPA1	OPA1	Mitochondrial dynamics and homeostasis	^903,904^
Barth syndrome	TAZ	Phospholipid transacylase	Mitochondrial membrane phospholipid maintenance	^432^
Friedreich’s ataxia	Frataxin	Frataxin	Mitochondrial iron-sulphur cluster synthesis	^446,448^
Fatty acid oxidation disorders	nDNA, for example, ACADM	Enzymes involved in fatty acid oxidation	Mitochondrial fatty acid oxidation	^468^
IOSCA	C10orf2	Twinkle or Twinky	MtDNA replication maintenance	^428^
Kearns-Sayre Syndrome	Single large-scale mtDNA deletions	OXPHOS subunits, mitochondrial tRNA and rRNA	Oxidative phosphorylation and mitochondrial translation maintenance	^323^
Leigh syndrome	nDNA and mtDNA such as, NDUFS4, MT-ATP6, and PDHA1	Subunits of complex I ~ V, pyruvate dehydrogenase, and other enzymes in energy metabolism	Oxidative phosphorylation and the broader process of energy production	^470,474^
LHON	mtDNA, for example, m.3460 G > A, m.11778 G > A, m.14484 T > C	Subunits of complex I	Oxidative phosphorylation	^238^
MELAS	MT-TL1	tRNA^Leu(UUR)^	Mitochondrial translation maintenance	^255,256^
MERRF	MT‑TK	tRNA^Lys(UUR)^	Mitochondrial translation maintenance	^278^
MIDD	MT-TL1	tRNA^Leu(UUR)^	Mitochondrial translation maintenance	^267^
MLASA	YARS2	Mitochondrial tyrosyl-tRNA synthetase	Mitochondrial translation maintenance	^505^
	PUS1	Pseudouridine synthase 1	Mitochondrial translation maintenance	^500^
MIRAS	POLG	MtDNA polymerase gamma	MtDNA replication maintenance	^410^
MNGIE	TYMP	Thymidine phosphorylase	Mitochondrial nucleotide pools maintenance	^39^
MCHS	POLG	MtDNA polymerase gamma	MtDNA replication maintenance	^905^
MEMSA	POLG	MtDNA polymerase gamma	MtDNA replication maintenance	^905^
NARP	MT-ATP6	Subunits of complex V	Oxidative phosphorylation	^291,292^
PEO	Single large-scale mtDNA deletions	OXPHOS subunits, mitochondrial tRNA and rRNA	Oxidative phosphorylation and mitochondrial translation maintenance	^303^
	nDNA, for example, POLG, PLOG2, C10orf2	Proteins involved in mtDNA maintenance	MtDNA replication maintenance, mitochondrial nucleotide pools maintenance, mitochondrial dynamics	^302^
Pearson syndrome	Single large-scale mtDNA deletions	OXPHOS subunits, mitochondrial tRNA and rRNA	Oxidative phosphorylation and mitochondrial translation maintenance	^345^
Perrault syndrome	ERAL	ERAL	Mitochondrial RNA processing and translation maintenance	^526^
	HARS2, LARS2	Aminoacyl tRNA synthetase	Mitochondrial translation maintenance	^523,524^
	CLPP	Caseinolytic protease P	Mitochondrial protein quality control	^516^
Sengers syndrome	AGK	Mitochondrial acylglycerol kinase	Mitochondrial membrane phospholipids metabolism and protein import machinery	^508^
SANDO	POLG	MtDNA polymerase gamma	MtDNA replication maintenance	^408^
	C10orf2	Twinkle	MtDNA replication maintenance	^412^

*mtDNA* mitochondrial DNA, *nDNA* nuclear DNA, *PEO* progressive external ophthalmoplegia, *MNGIE* mitochondrial neurogastrointestinal encephalomyopathy, *SANDO* sensory ataxia, neuropathy, dysarthria, and ophthalmoplegia, *MIRAS* mitochondrial recessive ataxia syndrome, *MEMSA* myoclonic epilepsy myopathy sensory ataxia, *MCHS* myocerebrohepatopathy spectrum, *IOSCA* infantile-onset spinocerebellar ataxia, *NARP* neurogenic muscle weakness, ataxia and retinitis pigmentosa, *LHON* Leber hereditary optic neuropathy, *ADOA* autosomal dominant optic atrophy, *MELAS* mitochondrial myopathy, encephalopathy, lactic acidosis and stroke-like episodes, *MERRF* myoclonic epilepsy with ragged red fibers, *MIDD* maternally inherited diabetes and deafness, *MLASA* myopathy, lactic acidosis and sideroblastic anemia

## Diagnostic methodology of mitochondrial diseases

**Fig. 6:**
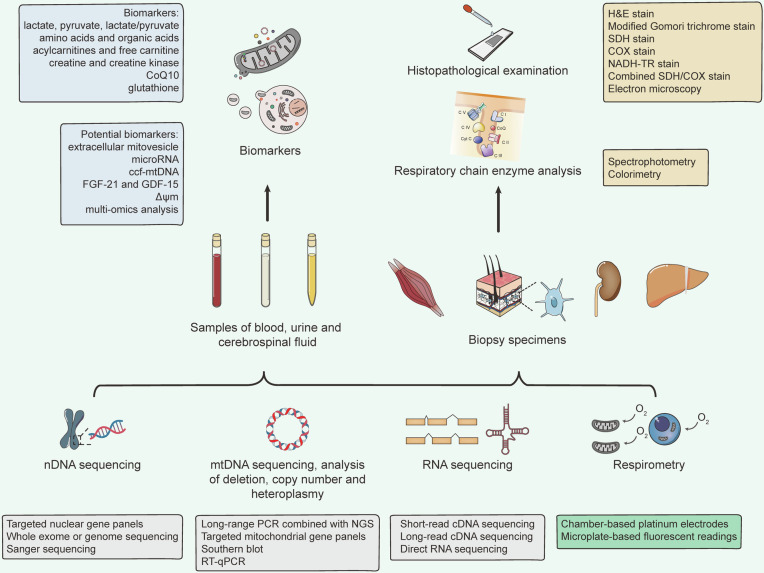
Diagnostic Methodology for Mitochondrial Diseases. Diagnostic strategies have evolved from a biopsy-first approach to a genetic-first approach. Initial screenings should utilize blood, urine, and cerebrospinal fluid samples. Biomarker testing is essential in this process. For suspected mitochondrial diseases, mtDNA sequencing and analysis are the preferred methods, while nDNA sequencing should be considered in cases of mtDNA multiple deletions, depletion, or early-onset symptoms. RNA sequencing (transcriptomics) and respirometry also contribute to accurate diagnosis. Biopsy specimens, typically obtained from muscle or skin, remain valuable for confirming tissue-specific mtDNA mutations that may not be detected in blood or urine samples. Histopathological examination and respiratory chain enzyme analysis can be applied to these tissue samples, revealing abnormal mitochondrial structure, morphology, and function. Thus, biopsy retains significant diagnostic value. mtDNA mitochondrial DNA; nDNA nuclear DNA; ccf-mtDNA circulating cell-free mitochondrial DNA; Δψm mitochondrial membrane potential; SDH succinate dehydrogenase; COX cytochrome c oxidase; NADH-TR nicotinamide adenine dinucleotide tetrazolium reductase

Given the genetic and phenotypic heterogeneity of mitochondrial genetic disorders, accurately diagnosing these diseases remains a significant challenge.^545^ Beyond careful and detailed clinical observation, comprehensive testing is essential, including biochemical analyses of body fluids, neuroimaging, DNA and RNA sequencing, as well as biochemical or pathological testing of tissues (Fig. 6). When a mitochondrial disease is suspected, initial biochemical testing should be performed on blood, urine, and cerebrospinal fluid.^545^ For instance, a blood count can reveal anemia or neutropenia, which are critical indicators in certain mitochondrial genetic disorders, such as Pearson syndrome.^343^

### Biomarkers

Certain biomarkers for mitochondrial diseases are garnering increasing attention due to their potential diagnostic value. The rise in anaerobic glycolysis, a compensatory response to energy shortages caused by OXPHOS dysfunction, leads to lactate accumulation, making lactic acidemia or lactic acidosis a common feature of these diseases.^544^ Since lactate is produced from pyruvate by lactate dehydrogenase, elevations in lactate are typically paralleled by increases in pyruvate.^546^ While lactate elevation exhibits high specificity (83–100%) among patients with mitochondrial diseases, its sensitivity is relatively low (34–62%), compared to pyruvate elevation, which shows a sensitivity of 75% and a specificity of 87.2%.^547,548^ The balance between lactate and pyruvate is regulated by the cytosolic NAD^+^/NADH ratio.^549^ Impaired oxidation of NADH, a function of respiratory complex I, increases NADH levels, driving the equilibrium toward lactate accumulation, which differs from the proportional increase in lactate and pyruvate seen in pyruvate dehydrogenase deficiency.^549^ Therefore, the lactate/pyruvate ratio offers excellent diagnostic accuracy for distinguishing pyruvate dehydrogenase deficiency from other mitochondrial diseases.^550^ Additionally, transaminase testing is essential as hepatopathy can be an early sign of mitochondrial diseases like Alpers-Huttenlocher syndrome.^394^

OXPHOS function measured in blood cells is emerging as a potential biomarker, with an estimated sensitivity of 68.3%, directly reflecting OXPHOS defects.^546^ The dysfunction of OXPHOS also leads to long-term alterations in Δψm as respiratory chain complexes fail to transfer protons.^83^ Notably, significant changes in Δψm were consistently observed in blood cell tests, suggesting that Δψm quantification could be a superior diagnostic method.^546^ Although less sensitive, biomarkers like creatine, creatine kinase, free carnitine, and acylcarnitine in the blood can aid in diagnosing mitochondrial diseases caused by fatty acid β-oxidation disorders.^546,547^ Additionally, amino acid analysis of both plasma and cerebrospinal fluid, along with organic acid analysis of urine, is valuable for diagnosing specific mitochondrial metabolic disorders, although patient selection prior to testing is recommended.^551^ For instance, an elevated monolysocardiolipins/tetralinoleoyl-cardiolipin ratio in blood and increased 3-methylglutaconic acid in urine (a precursor to cardiolipin) are strong biochemical indicators of Barth syndrome.^438^ Notably, two cytokines involved in the mt-ISR, FGF-21 and GDF-15, have shown exceptional diagnostic value for mitochondrial myopathy and tRNA gene mutation-related mitochondrial diseases, and they may also serve as biomarkers for monitoring therapeutic efficacy.^548,552,553^

The significance of extracellular mitochondrial content has been highlighted by *Miliotis* et al.^554^. Recent studies suggest that mitoEVs are involved in encephalopathy associated with mitochondrial disorders, potentially serving as indicators of mitochondrial encephalopathy.^555^ MitoEVs containing mitochondria or mtDNA are emerging as promising biomarkers,^554,556^ as their release is part of the process that recycles or eliminates nonfunctional mitochondrial fragments.^184^ Changes in ncRNAs, which regulate mitochondrial protein expression and mitochondrial function-related signaling pathways, can reflect metabolic and functional alterations in mitochondria.^557^ MicroRNAs, a specific type of ncRNA, play a role in the pathogenesis of mitochondrial diseases and could aid in diagnosis. For example, oxidative stress-induced microRNA-9/9* has been implicated in the MELAS phenotype, while downregulation of microRNA-181a/b, which promotes MQC through the activation of mitochondrial biogenesis and mitophagy, has shown protective effects on RGCs in an LHON mouse model.^558^ Further studies on microRNA-181a/b downregulation in the treatment of inherited retinal diseases underscore the importance of microRNAs.^559^ Additionally, microRNA-27b-3p has demonstrated significant diagnostic value in patients with MELAS.^560^ However, these biomarker changes are not exclusive to mitochondrial diseases and can also be observed in other conditions,^554,561^ necessitating further research into their sensitivity and specificity.

Furthermore, under conditions of stress, apoptosis, or necrosis, damaged mtDNA can be released from cells as cell-free circulating mtDNA (ccf-mtDNA).^562^ The accumulation of damaged mtDNA contributes to the increase of ccf-mtDNA in plasma, making it a potential biomarker for mtDNA genetic disorders, particularly MELAS, with a sensitivity of 44% and specificity of 94%.^554^

Given the profound impact of metabolic and functional disorders on genetic mitochondrial diseases, the fields of proteomics, lipidomics, and metabolomics are being explored for their potential applications.^563–567^ While their diagnostic value is not yet fully established, these approaches hold great promise for the future.

### DNA and RNA sequencing

Given that mtDNA mutations are the primary cause of genetic mitochondrial diseases, mtDNA testing should be prioritized in the diagnostic process. The advent of NGS has significantly shifted the diagnostic approach from a biopsy-first strategy to a genetic-first strategy.^568,569^ With the development and widespread application of NGS, the genetic diagnostic yield for these disorders has increased from 10–20% in the pre-NGS era to 40–60% today.^41,568^ However, the diagnostic yield of NGS in suspected mitochondrial disease cases varies, with reports indicating a range of 7% to 70%.^227^ Recently, the United Kingdom introduced new practice guidelines for the genetic diagnosis of mitochondrial diseases, aiming to standardize and guide the use of DNA sequencing.^569^

Comprehensive mtDNA testing should include sequencing the entire mtDNA genome, analyzing mtDNA deletions, assessing mtDNA copy number, and determining the heteroplasmy levels of mtDNA mutations.^7,547,551^ NGS has become the first-line methodology for mtDNA testing.^547^ Various laboratory techniques, such as polymerase chain reaction-restriction fragment length polymorphism (PCR-RFLP), allele-specific oligonucleotide polymerase chain reaction (ASO-PCR), single-strand conformation polymorphism (SSCP), long-range PCR, and Southern blot, have traditionally been used to screen for point mutations and deletions in mtDNA.^551^ For analyzing mtDNA copy number, which often reflects mtDNA depletion, real-time quantitative PCR (RT-qPCR) is commonly used.^551^ Assessing the heteroplasmy level of mutant mtDNA is also necessary, as it may correlate with disease severity and progression; this is typically done using methods like pyrosequencing or PCR,^546^ though these approaches are increasingly being replaced by NGS.^570^ A promising approach combines long-range PCR-based enrichment with NGS, enabling both quantitative and qualitative detection of every base in the entire mitochondrial genome.^570^ This method can detect heteroplasmy levels as low as 1–10%, with improved sensitivity due to specific mtDNA enrichment strategies and NGS platforms.^7^ However, it is essential to distinguish these findings from normal tissue states, which may carry low-level heteroplasmy.^228^ Although blood is the most commonly used sample for mtDNA testing, it is also advisable to assess mtDNA in urine and other affected tissues due to the possibility of tissue-specific mutations.^547^

As discussed, multiple mtDNA deletion and depletion are often caused by nDNA mutations. Consequently, 75–90% of pediatric mitochondrial diseases are attributed to nDNA mutations, making nDNA sequencing particularly important for diagnosing childhood-onset mitochondrial diseases.^551^ NGS of nuclear genes involved in mtDNA maintenance, mitochondrial function, and metabolism using targeted gene panels and whole exome sequencing is a preferred approach.^571^ Other sequencing methods also play vital roles; for example, Sanger sequencing remains a valuable first-line test for identifying common mutant genes in certain populations.^569^

Emerging RNA sequencing, or transcriptomics, has recently shown promise for diagnosing mitochondrial diseases.^571^ This technique serves as an essential complement to genome sequencing and can help diagnose suspected mitochondrial diseases that remain genetically undetermined after genome sequencing.^572^ RNA sequencing methods can be divided into short-read cDNA sequencing, long-read cDNA sequencing, and direct RNA sequencing.^573^ RNA sequencing provides insights into aberrant splicing and altered transcript levels due to abnormal gene expression or mono-allelic expression.^571^ For instance, RNA sequencing has detected a splicing variant in the *CLPP* gene associated with Perrault syndrome and mono-allelic expressed variants in the *ALDH18A1* gene, which encodes an enzyme involved in mitochondrial proline metabolism, linked to cutis laxa III.^572^ RNA sequencing also has identified splice site mutations in mitochondrial diseases.^574,575^ Furthermore, tRNA sequencing can uncover defective tRNAs and reduced tRNA levels, which are implicated in many mitochondrial diseases.^571^ For example, defective N1-methyladenosine (m^1^A) modification in mt-tRNA^Lys^ and mtDNA mutation-caused tRNA^Ala^ reduction, discovered through tRNA sequencing, have provided valuable insights into the pathogenesis of mitochondrial diseases.^69,280^

### Biopsy

A tissue biopsy is widely regarded as the diagnostic gold standard for mitochondrial diseases, particularly in cases where genetic testing has not provided a definitive diagnosis.^547^ Detecting OXPHOS or mtDNA defects in biopsy specimens provides compelling evidence for the presence of mitochondrial disease. Muscle tissue is often preferred for biopsy due to its high energy demand, making it particularly susceptible to OXPHOS dysfunction.^549^ Skin biopsies, which allow for the analysis of fibroblasts, are also a viable alternative.^576^ Genetic, histopathological, and biochemical evaluations of biopsy specimens are crucial in this diagnostic process.

Testing mtDNA in biopsy specimens offers greater sensitivity in detecting low-level mtDNA heteroplasmy and assessing mtDNA copy number compared to blood samples.^547^ Histological stains such as Haematoxylin and eosin (H&E) and modified Gomori trichrome are used to examine mitochondrial structure and morphology. The modified Gomori trichrome stain, in particular, can reveal ragged-red fibers, which indicate abnormal mitochondrial proliferation in response to energy failure, and can also highlight cytochrome c oxidase (COX, respiratory complex IV) deficiency.^577,578^ Sequential staining with succinate dehydrogenase (SDH, respiratory chain complex II) and COX provides additional insights into the activity of these complexes.^549^ Since SDH is entirely encoded by nuclear DNA, while mtDNA encodes three subunits of COX, COX activity is a more direct reflection of mtDNA maintenance.^579^ In normal tissue, SDH staining appears as ragged-blue fibers.^580,581^ Due to the uneven distribution of mtDNA mutations along muscle fibers, COX staining often shows a mosaic pattern of COX-negative and COX-positive fibers in cross-sections.^579^ In combined COX and SDH staining, the COX activity is typically indicated by a brown stain that overshadows the blue SDH stain. However, reduced COX activity allows the SDH stain to become more visible, presenting as blue fibers.^579^ This staining combination provides two key insights: a mosaic pattern of SDH-positive but COX-negative fibers indicates an mtDNA defect involving COX, while a mosaic pattern of both SDH-positive and COX-positive fibers suggests an mtDNA defect involving NADH dehydrogenase (respiratory complex I) or cytochrome c reductase (respiratory complex III).^579^ To specifically detect NADH dehydrogenase activity, nicotinamide adenine dinucleotide tetrazolium reductase (NADH-TR) staining should be employed.^582^ A reduction in NADH-dehydrogenase activity results in decreased or absent blue-purple color in NADH-TR staining, with an increase in stained ragged blue fibers in the subsarcolemmal region.^549^

In addition to assessing OXPHOS function in blood, as discussed earlier, the biochemical analysis of mitochondrial respiratory chain enzymes, isolated from muscle tissue or cultured fibroblasts, can directly reflect OXPHOS defects.^583,584^ CoQ10 assessment is also important, given its critical role in electron transport within OXPHOS.^585^ Spectrophotometric or colorimetric assays are commonly used to measure enzymatic activity.^584,586^ Successful enzymological analysis requires the use of internal controls, typically normalized to SDH activity or mitochondrial citrate synthase levels.^547,586^

Electron microscopy offers detailed visualization of mitochondrial quantity, inclusions, and ultrastructural abnormalities, demonstrating its diagnostic potential for mitochondrial nephropathy, cardiomyopathy, and hepatopathy.^587–589^

### Respirometry

High-resolution respirometry is a valuable methodology for assessing mitochondrial respiration, specifically OXPHOS function.^590^ This technique can be applied to various subjects, including isolated mitochondria, intact cells, and three-dimensional systems such as tissue slices, and it is typically measured using two primary setups: chamber-based platinum electrodes and microplate-based fluorescent readings.^591^ The substrate-uncoupler-inhibitor titration (SUIT) protocol is particularly effective in this context, as it allows for the measurement of oxygen consumption (flux) by specific respiratory complexes, thereby reflecting enzyme activity.^590,592,593^ By calculating flux control ratios—ratios of oxygen flux under different respiratory control conditions—the SUIT protocol enables internal normalization, facilitating comparisons across different studies.^593^ This method has shown promise in the diagnosis of mitochondrial diseases in several studies.^594–596^

However, there are no perfect diagnostic methodologies. Diagnosing atypical and novel genetic mitochondrial disorders requires a thoughtful combination of these various diagnostic approaches. Moreover, the importance of integrating clinical observations with laboratory examinations cannot be overstated, as this connection is crucial for accurate diagnosis.

## Potential therapeutic strategies of mitochondria in genetic disorders

**Fig. 7:**
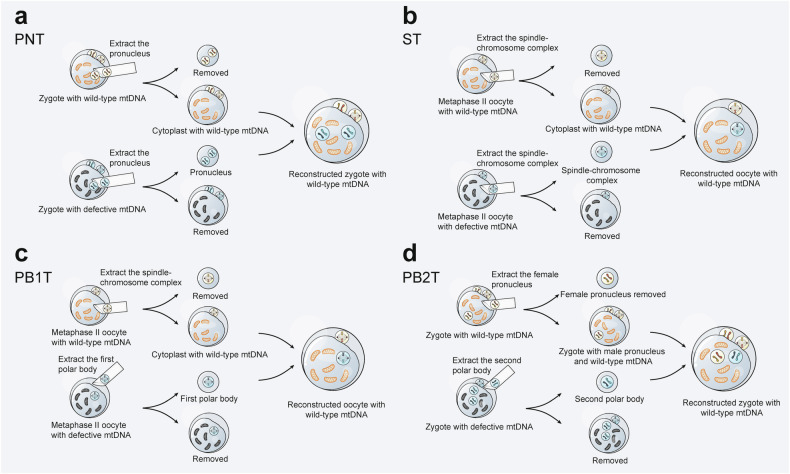
Procedure for Mitochondrial Replacement Therapy. **a** The procedure for pronuclear transfer (PNT) involves extracting the pronucleus from a zygote of a healthy donor with wild-type mtDNA and from a patient with defective mtDNA. The cytoplast from the patient and the pronucleus from the healthy donor are then removed. Finally, the pronucleus from the patient and the cytoplast containing wild-type mtDNA from the healthy donor are fused, reconstructing a zygote with the patient’s pronucleus and wild-type mtDNA. **b** The procedure for spindle-chromosome complex transfer (ST) involves extracting the spindle-chromosome complex from a metaphase II oocyte of a healthy donor with wild-type mtDNA and from a patient with defective mtDNA. The cytoplast from the patient and the spindle-chromosome complex from the healthy donor are removed. Finally, the spindle-chromosome complex from the patient and the cytoplast containing wild-type mtDNA from the healthy donor are fused, reconstructing an oocyte with the patient’s spindle-chromosome complex and wild-type mtDNA. **c** The first polar body transfer (PB1T) procedure involves extracting the spindle-chromosome complex from a metaphase II oocyte of a healthy donor with wild-type mtDNA and the first polar body from a metaphase II oocyte of a patient with defective mtDNA. The cytoplast from the patient and the spindle-chromosome complex from the healthy donor are then removed. Finally, the first polar body from the patient and the cytoplast containing wild-type mtDNA from the healthy donor are fused, reconstructing an oocyte with the patient’s first polar body and wild-type mtDNA. **d** The second polar body transfer (PB2T) procedure involves extracting the female pronucleus from a zygote of a healthy donor with wild-type mtDNA and the second polar body from a zygote of a patient with defective mtDNA. The zygote from the patient and the female pronucleus from the healthy donor are then removed. Finally, the second polar body from the patient and the zygote containing wild-type mtDNA and the male pronucleus from the healthy donor are fused, reconstructing a zygote with the patient’s second polar body and wild-type mtDNA

**Fig. 8:**
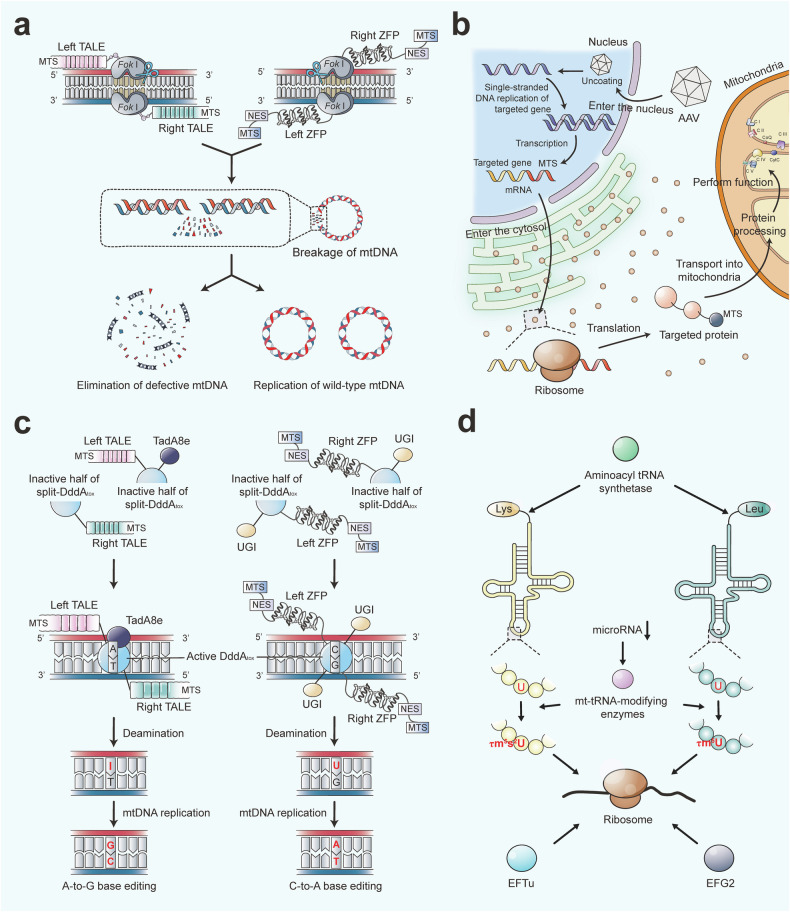
Gene Therapy and Post-Transcriptional Modification Strategies for Mitochondrial Genetic Disorders. **a** MitoTALENs consist of TALE fused with FokI nucleases, while mtZFNs are composed of ZFP linked to FokI nucleases. MTS guides mtZFNs and mitoTALENs to the mitochondria. ZFP and TALE selectively bind to predetermined defective mtDNA target sequences, after which FokI dimerizes and cleaves the mtDNA adjacent to these binding sites, causing double-strand breaks that lead to the elimination of defective mtDNA. The remaining wild-type mtDNA can then replicate, altering the heteroplasmy ratio. **b** The targeted gene sequence, along with transcriptional regulatory elements and MTS, is packaged into an AAV vector, which is then delivered to the nucleus of the recipient cell. Inside the nucleus, the AAV uncoats and releases single-stranded DNA, which replicates to form double-stranded DNA. RNA polymerase then transcribes this DNA into mRNA. The mRNA exits the nucleus and is translated into the corresponding protein at the ribosomes in the cytoplasm. MTS guides these proteins to the mitochondria, where they undergo further processing and perform their respective functions. **c** DdCBEs are engineered by fusing MTS, split-DddA_tox_ halves, UGIs, and either ZFPs or TALEs. DddA_tox_ catalyzes the deamination of cytosine to uracil, while UGIs prevent uracil-DNA glycosylase from excising uracil, resulting in C-to-T editing during replication. Additionally, by linking MTS, split-DddA_tox_ halves, TadA8e (an engineered adenine deaminase), and TALEs, TadA8e catalyzes the deamination of adenine to inosine, which pairs with cytosine during replication, thereby achieving targeted A-to-G editing. **d** Modifying the tRNA binding domain of nuclear-encoded aminoacyl tRNA synthetase or overexpressing aminoacyl tRNA synthetase can enhance aminoacylation efficiency and stabilize translation products. The expression of post-transcriptional negative regulators like microRNAs can inhibit the expression of mitochondrial RNA-modifying enzymes, thereby affecting mt-tRNA modifications. Using microRNA antagonists could potentially reverse disease phenotypes. Furthermore, overexpressing mt-tRNA-modifying enzymes can correct anticodon first nucleotide modification defects in mt-tRNA, improving ribosomal translation within mitochondria. Overexpression of mitochondrial translation elongation factors EFTu and EFG2 can also partially suppress amino acid misincorporation caused by mtDNA mutations during the translation elongation process. mtDNA mitochondrial DNA; mitoTALENs mitochondria-targeted transcription activator-like effector nucleases; mtZFNs mitochondria-targeted zinc-finger nucleases; MTS mitochondrial targeting sequence; NES nuclear export signal; DdCBEs DddA_tox_-derived cytosine base editors; ZFP zinc finger proteins; TALE transcription activator-like effector; UGIs uracil glycosylase inhibitors; TadA8e deoxyadenosine deaminase; AAV adeno-associated virus

Effective treatments for most mitochondrial diseases remain elusive. However, mitochondrial replacement therapy (MRT) and gene therapy represent promising foundational approaches for treating mitochondrial genetic disorders and preventing their transmission to future generations. Additionally, ongoing research is exploring the restoration of post-transcriptional tRNA modifications in mitochondria and the development of targeted therapies for specific conditions. Below, we provide a detailed summary of potential therapeutic strategies and discuss their possible clinical applications. (Fig. 7, Fig. 8, Table 3).

**Table 3:** Clinical trials for mitochondrial diseases

Conditions	Interventions	Phases	Study Status	Times	Countries	ID
Mitochondrial diseases	KH176	I	Completed	2015-2015	Belgium	NCT02544217
	KH176	II	Completed	2016-2017	Netherlands	NCT02909400
	KH176	II	Completed	2019-2022	Denmark, Germany, Netherlands, United Kingdom	NCT04165239
	KH176	II	Completed	2021-2023	Netherlands, Denmark, United Kingdom, Germany	NCT04604548
	KH176	II	Not_recruiting	2021-2025 (estimated)	Netherlands	NCT04846036
	EPI-743	I/II	Completed	2012-2019	United States	NCT01642056
	EPI-743	II/III	Completed	2020-2023	United States, Canada, France, Italy, Japan, Poland, Spain, Sweden, United Kingdom	NCT04378075
	EPI-743	III	Enrolling_by_invitation	2022-2025 (estimated)	United States, France, Italy, Japan, Poland, Spain, United Kingdom	NCT05218655
	OMT-28	II	Recruiting	2023-2025 (estimated)	Germany, Italy, Netherlands	NCT05972954
	KL1333	I	Completed	2019-2021	United Kingdom	NCT03888716
	KL1333	I	Completed	2020-2020	United Kingdom	NCT04643249
	KL1333	II	Not_recruiting	2022-2025 (estimated)	United States, Belgium, Denmark, France, Spain, United Kingdom	NCT05650229
	Elamipretide	II	Completed	2016-2017	United States	NCT02805790
	Elamipretide	III	Not_recruiting	2022-2024 (estimated)	United States, Australia, Germany, Hungary, Italy, Netherlands, New Zealand, Norway, Spain, United Kingdom	NCT05162768
	Coenzyme Q10	III	Completed	2007-2013	United States, Canada	NCT00432744
	Sodium succinate	II/III	Completed	2014-2019	Japan	JPRN-UMIN000013512
	N-acetylcysteine	I	Recruiting	2023-2024 (estimated)	United States	NCT05241262
	Cysteamine bitartrate	II	Completed	2014-2016	United States	NCT02023866
Leber hereditary optic neuropathy	NR082	I/II	Not_recruiting	2023-2029 (estimated)	United States	NCT05293626
	NR082	II/III	Recruiting	2021-2028 (estimated)	China	NCT04912843
	NFS-02	I/II	Recruiting	2023-2029 (estimated)	United States, China	NCT05820152
	Curcumin	III	Completed	2005-2007	Thailand	NCT00528151
	Idebenone	I/II	Completed	2013-2017	Japan	JPRN-UMIN000017939
	Idebenone	II	Completed	2007-2010	Canada, Germany, United Kingdom	NCT00747487
	Idebenone	III	Not_recruiting	2022-2025 (estimated)	China	ChiCTR2200059044
	Idebenone	IV	Completed	2016-2021	United States, Austria, Belgium, Bulgaria, Germany, Italy, Poland, Portugal, Spain, United Kingdom	NCT02774005
	Idebenone	IV	Completed	2018-2018	China	ChiCTR-IPR-17013821
	Bezafibrate	II/III	Completed	2019-2023	France	NCT04561466
	Elamipretide	II	Completed	2016-2019	United States	NCT02693119
	rAAV2-ND1	0	Completed	2021-2022	China	ChiCTR2000041574
	rAAV2-ND4	0	Completed	2020-2021	China	ChiCTR2000038570
	rAAV2-ND4	I/II	Completed	2014-2020	France	NCT02064569
	rAAV2-ND4	II/III	Not_recruiting	2017-2025 (estimated)	China	NCT03153293
	rAAV2-ND4	III	Completed	2016-2018	United States, France, Germany, Italy, United Kingdom	NCT02652780
	rAAV2-ND4	III	Completed	2016-2019	United States, France, Germany, Italy, United Kingdom	NCT02652767
	rAAV2-ND4	III	Completed	2018-2022	United States, France, Germany, Italy, United Kingdom	NCT03406104
	rAAV2-ND4	III	Not_recruiting	2018-2024 (estimated)	United States,Belgium, France, Italy, Spain, Taiwan, United Kingdom	NCT03293524
	scAAV2-P1ND4v2	I	Not_recruiting	2014-2024 (estimated)	United States	NCT02161380
	Skin electrical stimulation	I/II	Completed	2018-2019	Japan	JPRN-jRCTs052180066
Friedreich’s ataxia	A0001	II	Completed	2009-2011	United States	NCT01035671
	RT001	I/II	Completed	2015-2016	United States	NCT02445794
	RT001	III	Completed	2019-2021	United States	NCT04102501
	DT-216	I	Completed	2022-2022	United States	NCT05285540
	DT-216	I	Completed	2022-2023	United States	NCT05573698
	EPI-743	II	Completed	2012-2016	United States	NCT01728064
	EPI-743	II	Completed	2013-2016	United States	NCT01962363
	EPI-743	II	Not_recruiting	2022-2024 (estimated)	United States	NCT05485987
	EPI-743	II/III	Completed	2020-2023	United States, Australia, Brazil, Canada, France, Germany, Italy, New Zealand, Spain	NCT04577352
	EPI-743	III	Enrolling_by_invitation	2022-2027 (estimated)	United States, Australia, Brazil, Canada, France, Germany, Italy, New Zealand, Spain	NCT05515536
	EGb 761	II	Completed	2008-2011	France	NCT00824512
	TAK-831	II	Completed	2017-2018	United States	NCT03214588
	MIN-102	II	Completed	2019-2020	Belgium, France, Germany, Spain	NCT03917225
	MIB-626	II	Completed	2021-2022	United States	NCT04817111
	CTI-1601	I	Completed	2019-2020	United States	NCT04176991
	CTI-1601	I	Completed	2020-2021	United States	NCT04519567
	CTI-1601	II	Completed	2022-2023	United States	NCT05579691
	CTI-1601	II	Enrolling_by_invitation	2024-2027 (estimated)	United States	NCT06447025
	VP 20629	I	Completed	2013-2015	United States	NCT01898884
	Calcitriol	IV	Completed	2021-2023	Spain	NCT04801303
	Etravirine	II	Completed	2020-2023	Italy	NCT04273165
	Artesunate	I/II	Recruiting	2022-2023 (estimated)	France	NCT04921930
	Idebenone	I	Completed	2001-2006	United States	NCT00015808
	Idebenone	I	Completed	2004-2006	United States	NCT00078481
	Idebenone	II	Completed	2005-2007	United States	NCT00229632
	Idebenone	III	Completed	2006-2010	Austria, Belgium, France, Germany, Netherlands, United Kingdom	NCT00905268
	Idebenone	III	Completed	2007-2009	United States	NCT00537680
	Idebenone	III	Completed	2007-2012	Austria, Belgium, France, Germany, Netherlands	NCT00993967
	Idebenone	III	Completed	2008-2010	United States	NCT00697073
	Idebenone	III	Completed	2011-2012	Austria, Germany, Netherlands, United Kingdom	NCT01303406
	Pioglitazone	III	Completed	2008-2013	France	NCT00811681
	Deferiprone	I/II	Completed	2008-2009	Australia, Belgium, Canada, France, Italy, Spain	NCT00530127
	Deferiprone	II	Completed	2009-2011	Belgium, France, Italy, Spain	NCT00897221
	Resveratrol	I/II	Completed	2011-2012	Australia	NCT01339884
	Resveratrol	II	Completed	2019-2024	Australia	NCT03933163
	Epoetin alfa	II	Completed	2008-2009	Italy	NCT00631202
	Epoetin alfa	II	Completed	2013-2015	Italy	NCT01493973
	Rosuvastatin	Early_I	Completed	2016-2017	United States	NCT02705547
	Iron chelating	I/II	Completed	2005-2008	France	NCT00224640
	(+)-Epicatechin	II	Completed	2016-2018	United States	NCT02660112
	Interferon γ-1b	II	Completed	2013-2014	United States	NCT01965327
	Interferon γ-1b	II	Completed	2013-2014	Italy	NCT02035020
	Interferon γ-1b	II	Completed	2016-2017	No location data	NCT03888664
	Interferon γ-1b	III	Completed	2015-2016	United States	NCT02415127
	Interferon γ-1b	III	Completed	2015-2017	United States	NCT02593773
	Interferon γ-1b	III	Completed	2016-2017	United States	NCT02797080
	Omaveloxolone	I	Not_yet_recruiting	2023-2024 (estimated)	United States	NCT06054893
	Omaveloxolone	II	Not_recruiting	2015-2024 (estimated)	United States, Australia, Austria, Italy, United Kingdom	NCT02255435
	AAVrh.10hFXN	I/II	Recruiting	2022-2029 (estimated)	United States, Canada	NCT05445323
	AAVrh.10hFXN	I	Recruiting	2022-2029 (estimated)	United States	NCT05302271
	Methylprednisolone	Early_I	Completed	2015-2018	United States	NCT02424435
	Carbamylated erythropoietin	II	Completed	2009-2011	Austria, Germany, Italy	NCT01016366
	Bupropion and citalopram	IV	Completed	2012-2013	No location data	NCT01716221
Mitochondrial myopathy	REN001	I	Completed	2022-2022	England, United Kingdom	ISRCTN57533271
	REN001	II	Completed	2021-2023	United States, Australia, Belgium, Canada, Czechia, Denmark, France, Germany, Hungary, Italy, Netherlands, New Zealand, Norway, Spain, United Kingdom	NCT04535609
	Bocidelpar	II/III	Not_recruiting	2021-2025 (estimated)	United States	NCT04641962
	L-arginine	III	Completed	2008-2012	Japan	JPRN-jRCT2091220023
	Bezafibrate	II	Completed	2015-2017	United Kingdom	NCT02398201
	Elamipretide	I/II	Completed	2015-2016	United States	NCT02367014
	Omaveloxolone	II	Completed	2015-2017	United States, Denmark	NCT02255422
	Periodic acceleration	I	Recruiting	2023-2024 (estimated)	United States	NCT05569122
	Nicotinamide riboside	II	Recruiting	2023-2025 (estimated)	United States	NCT05590468
	Autologous mesoangioblasts	I	Completed	2020-2022	Netherlands	NCT05063721
	Autologous mesoangioblasts	II	Recruiting	2023-2025 (estimated)	Netherlands	NCT05962333
Mitochondrial myopathy, encephalopathy, lactic acidosis and stroke-like episodes	Taurine	III	Completed	2017-2019	Japan	JPRN-jRCTs061180015
	KL1333	I	Completed	2017-2018	Korea	NCT03056209
	Idebenone	II	Completed	2009-2012	United States	NCT00887562
	L-arginine	II	Completed	2012-2013	Canada	NCT01603446
	L-citrulline	I	Recruiting	2021-2024 (estimated)	United States	NCT03952234
	Zagociguat	II	Not_yet_recruiting	2024-2024 (estimated)	United States, Australia, Canada, Germany, Italy, United Kingdom	NCT06402123
	Arginine and citrulline	Early_I	Completed	2009-2016	United States	NCT01339494
	Medium-chain triglycerides	Early_I	Completed	2010-2011	United States	NCT01252979
Leigh syndrome	EPI-743	II	Completed	2012-2015	United States	NCT01721733
	EPI-743	II	Completed	2014-2023	United States	NCT02352896
	EPI-743	III	Completed	2014-2021	Japan	JPRN-jRCT2080222577
Pearson syndrome	MNV-201	I	Recruiting	2023-2027 (estimated)	Israel	NCT06017869
	MNV-BM-BLD	I/II	Completed	2019-2021	Israel	NCT03384420
Pyruvate dehydrogenase complex deficiency	Triheptanoin	I	Recruiting	2024-2027 (estimated)	United States	NCT06340685
	Dichloroacetate	III	Not_recruiting	2020-2025 (estimated)	United States	NCT02616484
	Sodium phenylbutyrate	II	Completed	2018-2020	Italy	NCT03734263
Barth syndrome	Elamipretide	II/III	Completed	2017-2021	United States	NCT03098797
Mitochondrial depletion syndrome	Deoxycytidine and deoxythymidine	II	Recruiting	2021-2026 (estimated)	Canada	NCT04802707

Data from ClinicalTrials.gov and International Clinical Trials Registry Platform

### Mitochondrial replacement therapy

#### Pronuclear transfer

Pronuclear transfer (PNT) is a technique that replaces the mitochondrial genome by transferring the parental pronucleus from zygotes with mutated mtDNA into enucleated zygotes containing healthy mitochondria.^597^ Initial studies show that less than 2% of the donor’s mtDNA persists in early embryos, and it becomes undetectable after development to the blastocyst stage in vitro.^598^ In mouse models with large-scale mtDNA deletions, PNT has successfully corrected mtDNA-related phenotypes in offspring,^599^ demonstrating its potential in treating mitochondrial genetic diseases. In 2015, the United Kingdom Parliament approved regulations allowing the use of PNT and ST, with other countries also exploring these techniques.^600^ However, the long-term effects on live-born offspring remain uncertain. Technical challenges, such as cytoplasmic leakage or incomplete separation of the nucleoplasm, may result in the transfer of a portion of the donor’s cytoplasm during PNT.^601^ Over time, the proportion of donor mtDNA may increase,^599,601^ potentially affecting the efficacy of MRT due to factors like enrichment, genetic drift, and mitochondrial bottleneck effects.^602–605^ This highlights the importance of minimizing or even eliminating mtDNA carryover during embryo transfer procedures.^605^ Vitrification of patient oocytes could help reduce mtDNA carryover and provide the option for oocyte storage.^601,606^ Additionally, female pronuclei, being smaller and containing fewer mitochondria than those used in PNT and ST, are easier to isolate and position, reducing the need for cytoskeletal inhibitors and avoiding premature oocyte activation.^607^ In a notable advancement, researchers isolated the female pronucleus from the second polar body and transferred it to another zygote, resulting in the live birth of four healthy cynomolgus monkeys.^608^ Moreover, techniques that enforce mitochondrial autophagy in reconstructed embryos produced by PNT have shown promise in reducing or eliminating mtDNA heterogeneity, enhancing safety.^605^

Despite these advancements, several potential issues require careful consideration. PNT results in the loss of approximately half of the embryos.^609^ While some ethical frameworks define pronuclear fusion as the beginning of embryonic life, PNT does not involve such fusion, which may reduce ethical concerns.^605,610^ Nevertheless, the loss of embryos remains a significant consideration.^605,610^ Furthermore, interactions between nDNA and mtDNA are extensive, with normal mitochondrial function relying on the coevolution of these two genomes.^611,612^ Disruption of these interactions post-PNT could potentially alter gene expression and phenotypic traits in offspring.^600^ This functional incompatibility between nuclear and mitochondrial genomes could even lead to reproductive isolation in mammals.^613^ To mitigate the risks associated with mito-nuclear interactions after mitochondrial replacement, it is recommended to match mitochondrial genotypes between donors and recipients and to determine acceptable variation levels between donor and patient mtDNA haplotypes.^600^ Until the implications of these interactions are better understood, combining mitochondrial replacement therapy with prenatal screening is advised.^601^ Ongoing follow-up and research will be essential to fully understand and address these issues.

#### Spindle-chromosome complex transfer

A study comparing mtDNA carryover and embryo development outcomes using germinal vesicle nuclear transfer, ST, and PNT in a mouse model^614^ found that germinal vesicle nuclear transfer did not result in blastocyst formation, while PNT and ST performed comparably well.^614^ ST involves replacing mutated mtDNA in patient oocytes by isolating the spindle-chromosome complex from the patient’s unfertilized mature MII oocytes and transferring it into the cytoplasm of enucleated healthy oocytes.^597^ Notably, *Tachibana* et al. successfully employed ST to produce healthy rhesus monkey offspring, which contained nDNA from the spindle donor and nearly homogenous mtDNA from the cytoplasm donor.^44^ In human studies, ST performed on unfertilized mature MII oocytes resulted in zygotes and derived embryonic stem cell lines with normal euploid karyotypes and donor-only mtDNA.^615^ Furthermore, the transfer of vitrified spindles into fresh cytoplasm led to the birth of four healthy rhesus monkey infants, with no significant changes in mtDNA heterogeneity.^615^ The use of cryopreserved human oocytes for ST has also been shown to eliminate mtDNA heteroplasmy while maintaining normal mitochondrial activity.^616^ Cryopreservation primarily affects the oocyte cytoplasm rather than the spindle, making it suitable for storing patient oocytes.^615^

There has been a reported case of a female patient with Leigh syndrome, carrying the m.8993 T > G mutation, giving birth to a healthy child using ST technology.^46^ However, the live birth following ST involved electrofusion,^46^ a technique that can prematurely activate the oocyte to enter late anaphase II, potentially leading to incomplete meiotic recovery post-fertilization and increasing the risk of abnormal pronuclear formation and aneuploidy.^615^ To mitigate the risk of premature oocyte activation, researchers have explored alternative approaches such as using chemical or mechanical methods,^617^ lowering temperatures to induce partial depolymerization of the spindle-chromosome complex in mature MII oocytes,^616^ and adjusting the sequence of intracytoplasmic sperm injection and ST.^618^ Despite the potential of ST, the procedure can result in donor mtDNA carryover.^44,609^ Although low-level mtDNA heteroplasmy often diminishes during MRT, genetic drift can sometimes cause mtDNA to revert to its original genotype, compromising the effectiveness of MRT.^602,619^ This reversion may be linked to the preferential replication of specific D-loop conservative sequence box II region polymorphisms.^619^ To address this, ongoing research is being conducted to reduce mitochondrial carryover and enhance the efficiency and safety of MRT.^620^ Strategies such as lowering cytochalasin B concentration before ST and PNT,^621^ generating aggregated chromosomes using the phosphodiesterase inhibitor 3-isobutyl 1-methylxanthine followed by transfer,^622^ and implementing ST with maximal residue removal in MII oocytes^618^ have shown promise in significantly reducing residual mtDNA levels. While ST is considered safe and effective^44,618^ and ethically acceptable,^623^ the optimal methodology has yet to be determined, and the risks of genetic drift necessitate further research.^623^

#### Polar body transfer

*Wang* et al. demonstrated that germline genomes derived from ST, PNT, and polar body transfer (PBT) in mice could lead to embryos capable of normal fertilization and producing viable offspring.^609^ The F1 generation from PBT exhibited minimal donor mtDNA carryover, which remained stable into the F2 generation.^609^ Notably, blastocysts resulting from first polar body transfer (PB1T) displayed lower average levels of mtDNA carryover compared to those from second polar body transfer (PB2T).^624^ Embryonic stem cells derived from PB1T blastocysts maintained low and stable mtDNA carryover during extended proliferation and differentiation, both in vitro and in vivo.^624^ Due to the mitochondrial inheritance bias during meiosis, polar bodies harbor minimal to undetectable mitochondria.^609^ PBT proceeds without the need for cytoskeletal disruptors,^624^ thereby further reducing donor mtDNA carryover. Additionally, the membrane encasing polar bodies protects the genome and facilitates their isolation.^609,624^ Combining PBT with ST or PNT in individual donor oocytes could potentially halve the number of donor oocytes needed, thereby improving MRT efficiency.^609^ Given that polar bodies and oocytes share the same genome, they might be more suitable as nuclear transfer donors than pronucleus or spindle-chromosome complexes.^609^ *Wang* et al. also demonstrated that PB1T could effectively substitute ST, yielding healthy macaque monkeys with stable mtDNA heteroplasmy below 5% and no mtDNA drift.^625^ However, due to the higher mitochondrial concentration in dense clusters within the first polar body in intact oocytes—compared to the uniform mitochondrial distribution in the ooplasm in ST—and the slightly larger volume of the first polar body relative to ST, the isolated first polar body contained more mtDNA than the isolated spindle complex,^625^ contrary to findings in mice.^609^ Nonetheless, polar bodies are distinct byproducts of meiosis, tasked with extruding the surplus genome, which might affect their quality.^626^ Further refinements in PBT techniques and additional preclinical studies on human oocytes and zygotes are necessary to assess the safety, efficacy, and feasibility of PBT.^620^

### Gene therapy

#### Allotopic expression

Mitochondrial proteins, encoded by nuclear genes, are synthesized in the cytoplasm before being imported into mitochondria.^627^ This process has propelled advances in treating mitochondrial genetic diseases through the allotopic expression approach. This method re-encodes defective mtDNA gene sequences into nuclear-compatible sequences, aligned with the “universal” genetic code.^628,629^ These sequences are combined with appropriate transcriptional regulatory elements and mitochondrial targeting peptides, then delivered to the cell nucleus *via* suitable vectors.^628,629^ The resultant mRNA is translated in the cytosol,^628,629^ after which the mitochondrial targeting peptides facilitate the import of these proteins into mitochondria, achieving the allotopic expression of functional mtDNA genes to compensate for genetic defects.^628,629^ Specifically, re-engineered and stabilized allotopic expression of *MT-ATP6* and *MT-ATP8* allows their processing, import, and integration into complex V,^630–632^ while *MT-ND3*,^633^ *MT-ND4*,^628^ and *MT-ND6*^634^ are successfully imported into mitochondria and incorporated into complex I, rescuing mitochondrial dysfunction caused by corresponding mtDNA mutations. These mutations can lead to the loss of mitochondrial proteins, defects in respiratory chain complexes, and impaired oxidative phosphorylation.

Although human tRNA is typically not imported into mitochondria,^635^ *Kolesnikova* et al. demonstrated partial mitochondrial import and proper aminoacylation of nDNA-encoded yeast tRNA^Lys^ derivatives in human cells, partially restoring mitochondrial function in MERRF cells.^636^ Furthermore, the *Leishmania* RNA import complex, entering human cells *via* a caveolin-1-dependent pathway, can induce the import of endogenous cytosolic tRNA, encoded by the nucleus, into mitochondria, rescuing dysfunction caused by mutated mt-tRNA genes.^637^

In LHON mice or rats, intravitreal injection of adeno-associated virus (AAV) vectors carrying nuclear-encoded human *MT*-*ND4* genes resulted in the allotopic expression of the wild-type *MT-ND4* gene, with subsequent accumulation of mRNA and protein in RGCs and optic nerve axons, successfully importing them into mitochondria.^629,638^ The ND4 protein assembles with three complex I subunits, integrating into the respiratory chain complex I without disrupting its activity.^629^ This integration preserves complex I function, preventing vision loss, RGC apoptosis, and degeneration, as well as optic nerve atrophy induced by mtDNA mutations.^629,639^ AAV-mediated allotopic expression of the nuclear-encoded *MT*-*ND4* gene appears to be a feasible and safe treatment for LHON with mutated mtDNA, with clinical trials underway.^47^ However, the timing of gene therapy is crucial, as oxidative damage, RGC apoptosis, and axonal loss may be partly irreversible.^639^ Therefore, administering gene therapy after vision loss but before optic nerve atrophy, or targeting the asymptomatic contralateral eye in patients with acute unilateral vision loss, may offer benefits.^628,639^

Despite the promise of allotopic expression, challenges remain. Only a small fraction of allotopically expressed mitochondrial proteins localize to mitochondria^632,640^ and functionally integrate,^641^ likely due to the high hydrophobicity of these proteins, which hinders efficient mitochondrial import.^640^ This necessitates further refinement in processing and import efficiency.^632,642^ Combining recoded mtDNA sequences with optimized 3’ untranslated regions and 5’ mitochondrial targeting sequences (MTS) can enhance protein localization and import.^629,642^ The importability of hydrophobic peptides may also be improved by enhancing the expression of molecular chaperones.^640^ Additionally, codon optimization of mitochondrial genes can improve the efficiency and stability of recoded mtDNA gene expression in the nucleus.^632^ LHON is characterized by a 100% m.11778 G > A mutation. Although LHON mammalian models exhibit symptoms of acute vision loss similar to human LHON, the LHON model retains a normal *MT-ND4* gene.^638^ Continued research is needed to explore the therapeutic potential of allotopic expression for other mitochondrial diseases.

#### Gene replacement therapy

Currently, the application of gene replacement therapy for various mitochondrial genetic diseases is the subject of extensive research and development. Normal genes can be delivered *via* AAV transduction to replace defective nuclear genes in mitochondrial genetic disorders, achieving therapeutic effects. Frataxin plays a key role in Fe-S cluster biosynthesis.^643^ In Friedreich’s ataxia, Frataxin deficiency leads to primary Fe-S cluster defects, reducing enzyme activity associated with these clusters, resulting in mitochondrial iron accumulation, dysfunction, and cellular damage.^644^ An AAV vector containing the *Frataxin* gene facilitates the expression of human *Frataxin* in Friedreich’s ataxia mouse models, restoring Fe-S cluster-associated protein levels and enzyme activity in cardiomyocytes.^644,645^ This restoration normalizes Fe-S biosynthesis, corrects iron accumulation, improves mitochondrial ultrastructure and abnormal cardiac myofibrils, and thus prevents and reverses the cardiomyopathy phenotype.^644,645^ However, therapeutic outcomes are highly dependent on the cardiac biodistribution of the vector.^646^ Furthermore, the therapeutic window for AAV-mediated *Frataxin* gene therapy is narrow; overexpression of *Frataxin* may induce oxidative stress and significantly increase labile iron pool levels, leading to hepatotoxicity and cardiotoxicity.^647^

A single intrathecal administration of AAV9/hSURF1 partially restores complex IV levels and activity, showing potential for treating *SURF1*-related Leigh syndrome.^648^ Additionally, intravenous and intracerebroventricular injections of AAV2/9-hNDUFS4 enhance complex I activity, improving weight, motor function, and lifespan in *NDUFS4* knockout Leigh syndrome mouse models.^649^ However, neither intravenous nor intracerebroventricular administration alone fully improved the clinical phenotype, indicating limitations in standard AAV vector transduction.^649^ In contrast, intravenous injection of the brain-penetrating AAV.PHP.B-NDUFS4 vector restored mitochondrial complex I activity and function, improved behavior, corrected brain, retina, and heart pathologies, restored weight, and extended lifespan in mice.^650,651^ However, the AAV.PHP.B vector’s efficacy is limited to certain mouse strains and is not applicable to primates.^652,653^ The use of self-complementary AAV9 vectors, effective across mammals and enhancing the transcription rate of recombinant human *NDUFS4*, restored complex I activity and assembly in Leigh syndrome mice, significantly extending their lifespan.^654^

Delivering CRISPR/Cas9 and *TYMP* cDNA *via* lipid nanoparticles, polymeric nanoparticles, or AAV2/8 viral vector can efficiently integrate *TYMP* into the *TYMP* and *Alb* loci of hepatocytes in the MNGIE mouse model.^655^ This approach increases TP activity in plasma, reduces nucleoside levels, and shows promise for treating MNGIE.^655^ AAV-TYMP has been shown to elevate hepatic TP activity in MNGIE mouse models, normalize nucleoside and mitochondrial nucleotide metabolism, enhance mtDNA replication, correct mitochondrial dysfunction, and alleviate functional phenotypes.^656^ The alpha-1-antitrypsin promoter, demonstrating optimal efficacy,^497^ could also minimize the dosage required for clinical effectiveness.^656^

Moreover, the AAV2/8 vector can mediate the expression of the human *ETHE1* gene in the liver of ethylmalonic encephalopathy mouse models, effectively clearing circulating hydrogen sulfide, correcting plasma thiosulfate levels, restoring sulfur dioxygenase activity, significantly improving disease phenotypes, and extending lifespan—highlighting its potential for future clinical applications in treating ethylmalonic encephalopathy.^657^

In Barth syndrome cells, AAV-TAZ transduction increases mtDNA copy number and enhances mitochondrial structure and function.^658^ Similarly, AAV9-TAZ ameliorates mitochondrial structural defects in a Barth syndrome mouse model, improving cardiac and skeletal muscle function^659,660^ and rescuing neonatal mice from mortality, cardiac dysfunction, and fibrosis. However, its efficacy and duration depend on the number of cardiomyocytes transduced.^660^ Notably, administration of AAV9 following low-intensity aerobic exercise can enhance AAV transduction efficiency in the heart and skeletal muscles.^661^

#### RNA-based therapy

Delivering double-stranded RNA, single-stranded silencing RNA, or antisense oligonucleotides (ASOs) to Friedreich’s ataxia cells can specifically target or excise the intronic GAA trinucleotide repeat sequence, thereby reducing R-loop formation between the expanded repeat RNA and complementary genomic DNA.^662–664^ This intervention reverses the transcriptional silencing of *Frataxin*, decreases the production of aberrant early-terminated *Frataxin* transcripts, and increases both Frataxin mRNA and protein levels, presenting a promising therapeutic strategy for Friedreich’s ataxia.^662–664^ Gapmer oligonucleotides complementary to the adenine-adenine-guanine repeat sequence within the *Frataxin* gene have shown a higher efficacy in activating *Frataxin* RNA and protein expression.^665^ Research by *Li* et al. indicates that co-delivering oligonucleotides targeting the 5’ or 3’ untranslated regions of *Frataxin* can extend the mRNA half-life, leading to increased steady-state levels of *Frataxin* mRNA and protein, suggesting a novel approach to upregulating mRNA levels in any transcriptionally downregulated disorder.^666^ Despite the successful delivery of ASOs in Frataxin mouse models, the anticipated increase in *Frataxin* expression was not observed, possibly due to the limited potency of the compounds, differences in the regulatory mechanisms of the *Frataxin* gene,^667^ or off-target effects.^668^ Additionally, two phosphorothioate-based ASOs with G-rich motifs were identified^668^ that indirectly activate *Frataxin* expression in Friedreich’s ataxia cells but similarly failed to induce *Frataxin* expression in Friedreich’s ataxia mouse models.^668^ Thus, determining the efficacy of double-stranded RNA, single-stranded silencing RNA, and ASOs in animal models is essential, necessitating further investigation.

#### Peptide nucleic acid oligomers

PNAs were the pioneering tool for achieving mitochondrial heteroplasmy shift.^669^ These molecules can selectively bind to complementary DNA or RNA sequences, effectively inhibiting replication and translation.^670^ Researchers have synthesized PNAs that are complementary to human mtDNA templates containing deletion breakpoints or single-base mutations, which specifically inhibit the replication of mutant human mtDNA templates in vitro.^42^ To address the challenge of delivering PNAs to mitochondria within cells, *Muratovska* et al. conjugated an 11-mer PNA to a lipophilic phosphonium cation.^670^ This phosphonium-PNA conjugate is non-cytotoxic, remains stable within cells, and can selectively inhibit the in vitro replication of mtDNA carrying the human m.8344 A > G mutation associated with MERRF.^670^ Despite the promising results PNAs have shown in inhibiting mutant mtDNA replication, their broader application is constrained. A PNA must be at least 7-mer in length to target a unique site within mtDNA.^669^ Additionally, during mtDNA replication, nucleic acid derivatives may fail to bind to their complementary sequences, and it remains uncertain whether single-stranded mtDNA at the replication fork can effectively interact with PNAs.^669,670^ Consequently, the widespread use of PNAs remains limited.

#### Mitochondria-targeted restriction endonucleases

Pathogenic mtDNA variations can introduce unique restriction endonuclease cleavage sites.^671^ MTS directs the restriction endonucleases into mitochondria, which cleaves pathogenic mtDNA at specific recognition sites, resulting in double-strand breaks (DSBs) that lead to the elimination of defective mtDNA.^671–673^ This process allows the remaining wild-type mtDNA to replicate, thereby altering the degree of heteroplasmy and offering a potential treatment for mitochondrial genetic disorders.^671–673^ Early research in 2002 demonstrated that mitochondria-targeted *Sma*I restriction endonuclease could selectively eliminate mutated mtDNA, allowing the replication of wild-type mtDNA and restoring normal cellular ATP levels and Δψm.^674^ Subsequent in vivo and in vitro studies have shown that mitochondria-targeted restriction endonucleases, such as *Pst*I,^675^ ApaLI,^676^ Scal,^677^ and R.*Xma*I,^678^ can effectively eliminate mutant mtDNA at corresponding sites, thereby facilitating a shift in heteroplasmy. These interventions resulted in notable improvements in ATP synthase function and alleviated mitochondrial dysfunction, demonstrating the potential of mitochondria-targeted restriction endonucleases (mtREs) to prevent disease onset or reverse clinical symptoms in patients with specific pathogenic heteroplasmic mtDNA mutations. Additionally, they may inhibit the transgenerational transmission of human mitochondrial diseases.^673,676,678–680^ Compared to other gene-editing technologies, mtREs offer a significantly higher specificity, minimizing off-target activity and preventing mtDNA copy number depletion.^681^ However, two major challenges may limit the application of this method. First, the cellular delivery of mtREs could raise safety concerns.^680^ Second, the utility of mtREs is restricted to targeting specific heterogeneous mtDNA mutations.^680^ Many mtDNA mutations do not create new restriction enzyme sites, rendering mtREs ineffective against these mutations,^42^ which hinders their clinical application.^669^ To extend this approach to other pathogenic mtDNA mutations, the development of nucleases with novel specificities is essential.^679^

#### Mitochondria-targeted zinc-finger nucleases

Zinc finger nucleases (ZFNs) consist of zinc finger proteins (ZFPs) linked to FokI nucleases, with ZFPs further connected to MTS and nuclear export signals (NES). These modifications guide mitochondria-targeted zinc-finger nucleases (mtZFNs) to the mitochondria, where ZFPs can selectively bind to specific DNA target sequences. Upon binding, FokI nucleases dimerize and cleave the DNA adjacent to the ZFP binding sites.^669,682^ While traditional ZFNs can target mtDNA, identifying suitable ZFN pairs for certain mutations poses a challenge. To address this, researchers developed heterodimeric ZFNs that bind both mutant and adjacent wild-type mtDNA sequences. However, this approach often results in the degradation of wild-type mtDNA and rapid mtDNA depletion. To overcome this, researchers created single-chain ZFNs by conjugating two FokI nuclease catalytic domains to ZFPs. These single-chain ZFNs demonstrated greater selectivity for pathogenic point mutations in mtDNA^682^ and proved more effective than their single-domain counterparts.^682^ Despite this improvement, single-chain ZFNs are ineffective against large mtDNA deletions and present potential safety concerns.^683^ To address these limitations, researchers redesigned conventional dimeric mtZFNs, ensuring that monomers did not affect mtDNA.^683^ The improved mtZFNs effectively eliminated point mutations and large-scale mtDNA deletions, reducing the mutant mtDNA haplotype load below the pathogenic threshold, thereby restoring OXPHOS function and improving mitochondrial respiration.^683^ Both in vitro and in vivo experiments with mtZFN-AAV targeting the m.5024 C > T tRNA^Ala^ mutation demonstrated a partial shift in heteroplasmy, leading to improved steady-state levels of mt-tRNA^Ala^, as well as enhanced mitochondrial respiration and metabolic function.^672^ Additionally, *Gammage* et al. achieved near-complete correction of mtDNA mutations and rescued mitochondrial respiratory function and metabolic defects through either consecutive short-term mtZFN treatments or finely controlled, optimized mtZFN expression.^681^ This approach minimized off-target effects and unwanted depletion of mtDNA copy numbers, proving more efficient than mtREs and mitochondria-targeted transcription activator-like effector nucleases (mitoTALENs).^681^ Despite these advancements, ZFN expression is associated with cytotoxicity due to off-target site cleavage. This issue might be mitigated by equipping mtZFNs with tightly regulated expression systems or by optimizing NES and ZFP sequences to reduce cytotoxicity.^682,684^

#### Mitochondria-targeted transcription activator-like effector nucleases

MitoTALENs are composed of targeted transcription activator-like effectors (TALEs) that bind to specific DNA sequences, coupled with FokI nucleases that dimerize to cleave mtDNA. Numerous in vitro and in vivo studies have demonstrated that mitoTALENs can reduce the load of pathogenic mtDNA and rescue associated functional phenotypes.^685,686^ Additionally, mitoTALENs have shown effectiveness in reducing the human m.3243 A > G mtDNA mutation in porcine oocytes,^685^ as well as the NZB mtDNA in MII oocytes of the NZB/BALB heterozygous mouse model.^680^ Targeting human m.14459 G > A and m.9176 T > C mutant mtDNA using mitoTALENs has led to the specific elimination of these mutant mitochondrial genomes.^680^ These results highlight the potential of mitoTALENs for selectively eliminating mutant mtDNAs and preventing their germline transmission. Furthermore, mitoTALEN nickases, which are derivatives of mitoTALEN with an inactive FokI domain on one monomer, can induce single-strand breaks at specific sites in human mtDNA. This process leads to mtDNA deletions^687,688^ and facilitates the creation of new animal models for studying single large-scale mtDNA deletion diseases.^689^

Although mitoTALENs may not be as potent as mtREs in preventing the spread of germline mitochondrial diseases,^680^ they offer greater design flexibility compared to mtREs and mtZFNs.^690^ However, mitoTALENs come with several limitations. Unlike more precise gene-editing technologies, ZFN and TALEN are incapable of performing precise single-base editing, making them unsuitable for correcting homogenous mtDNA mutations.^691^ Additionally, most mitoTALENs require a thymidine base at position 0 of the target DNA binding site,^692^ and the mutation’s sequence context, along with methylation or other epigenetic modifications of the mtDNA target sequence, can impact the TALE’s efficiency.^693,694^ The large size of mitoTALENs also complicates their encapsulation in many vector systems.^695,696^ Some researchers have attempted to overcome this by fusing a monomeric nuclease domain derived from the I-TevI homologous endonuclease to the TALE DNA-binding domain^697^ or by designing shorter, more specific mitoTALENs.^698^ However, these approaches often limit DNA sequence recognition.^698^ Another significant challenge is the potential for non-specific cleavage by mitoTALENs, which can lead to substantial depletion of mtDNA copies and induce cytotoxicity,^314,692^ necessitating precise dose control of the constructs.^681^ Moreover, while mitoTALENs exhibit minimal cleavage activity against wild-type mtDNA,^699^ further development is needed to create more effective, safer, and easier-to-deliver mitoTALENs in the future.

#### CRISPR/Cas9

The design of single-guide RNA (sgRNA) for targeting mtDNA mutations involves adding MTS upstream and downstream of the Cas9 gene and the 3’ untranslated region of the target gene.^700,701^ This configuration enables mitochondria-targeted Cas9 (mito-Cas9) to cleave mtDNA at specific sites dictated by the sgRNA.^700,701^ It has been shown that the mito-Cas9 system can be successfully translocated into mitochondria, where it can introduce exogenous single-stranded DNA oligonucleotides into mtDNA, thereby facilitating the creation of cellular models of disease-causing mtDNA mutations.^701^ However, the efficiency of mtDNA editing using mito-Cas9 systems can be limited by several factors, including the sequence characteristics of the target region and the variable targeting efficiencies of different sgRNAs.^701^ Optimizing the mito-Cas9 system might involve enhancing mitochondrial RNA transport^702^ or employing engineered Cas proteins with higher editing efficiency.^703^ Nevertheless, the lack of an RNA transporter system within the double-membrane structure of mammalian mitochondria^31,704^ results in inefficient or defective nucleic acid import into mitochondria.^691,700^ Additionally, the absence of homologous recombination and non-homologous end-joining pathways for repairing DSBs in mitochondria further complicates the manipulation of mtDNA.^689,705^ In summary, any attempt to import synthetic RNA molecules into mitochondria based on naturally occurring mechanisms in human cells is likely to be sporadic and inefficient.^681^ Consequently, the application of CRISPR/Cas9 technology to mtDNA manipulation remains highly challenging and will require further extensive research to overcome these obstacles.

#### Mitochondria-targeted meganucleases

Mitochondria-targeted meganucleases (mitoARCUS), derived from the naturally occurring I-CreI nucleic acid endonucleases, are small, highly specific single-component proteins.^706^ These nucleases possess the ability to recognize DNA sequences with single-base pair differences, generating DSBs. They can be efficiently packaged into individual viral vectors, requiring only minimal AAV titers, and do not show signs of vector-associated or transient mtDNA depletion toxicity.^707,708^ In heterozygous mice with the m.5024 C > T mutation, AAV9-mitoARCUS significantly altered heteroplasmy and restored mt-tRNA^Ala^ levels.^708,709^ Additionally, AAV9-mitoARCUS modulated heterogeneity and enhanced mitochondrial-encoded protein homeostasis and respiratory function in m.3243 A > G cell lines and mouse models.^707^ However, the need to redesign target-specific mitoARCUS for each mutation and the complexity involved in reengineering I-CreI to recognize new targets remain substantial challenges that must be addressed.^705,707,710^

#### DddA-derived cytosine base editors

DddA-derived cytosine base editors (DdCBEs) are engineered by fusing MTS, split-DddA_tox_ halves, TALE, and uracil glycosylase inhibitors.^710^ The DddA_tox_ enzyme catalyzes the conversion of cytosine to uracil,^710^ while the uracil glycosylase inhibitors prevent uracil-DNA glycosylase from removing uracil, leading to C-to-T mutations during subsequent DNA replication without introducing DSBs. This mechanism ensures high specificity and product purity in targeted edits 715. DdCBEs have facilitated mitochondrial base editing in human embryos^711,712^ and enabled precise, heritable C-to-T base editing at specific mtDNA sites in zebrafish, mice, and rats. These models replicate phenotypes akin to human mitochondrial diseases, thereby advancing the precise modeling of these conditions.^710,713–716^ Additionally, *Mok* et al. developed monomeric DdCBEs derived from non-toxic, full-length DddA_tox_ variants, achieving C-to-T editing with reduced off-target effects.^717^ Zinc finger deaminases, which combine zinc finger DNA-binding proteins, DddA_tox_, and uracil glycosylase inhibitors, also catalyze targeted C-to-T transitions in mtDNA.^718^ Optimizing the architecture of zinc finger DdCBEs has improved editing efficiency, reduced off-target effects, facilitated packaging in AAV vectors, and potentially decreased immunogenicity.^719^ These advances enable the correction of pathogenic mtDNA mutations and the modeling of mitochondrial diseases.

Ongoing research focuses on enhancing the base editing efficiency of DdCBEs and expanding their applications. For example, co-injection of mitoTALEN has been shown to enhance DdCBE-NES-mediated mtDNA editing.^720^ Variants of DddA with increased base editing efficiency and a broader target range have been developed using phage-assisted continuous evolution and phage-assisted discontinuous evolution.^720,721^ The DddA11 variant, in particular, has expanded HC (H = A, C, or T) sequence compatibility, although it remains less effective for GC targets.^722^ CRISPR-mediated nuclear and TALE-based mitochondrial DdCBEs, utilizing dsDNA deaminase derived from *Roseburia intestinalis* interbacterial toxin (riDddA_tox_), have successfully achieved C-to-T editing of HC and GC targets.^723^ Fusion of transactivators to DddA_tox_ or riDddA_tox_ has significantly increased editing efficiency in both nDNA and mtDNA.^723^ Moreover, combining the DddA11 variant with activation-inducible cytidine deaminase has further improved C-to-T editing efficiencies across various targets.^724^ In another approach, TALE-linked deaminases designed by *Cho* et al. catalyze the hydrolytic deamination of adenine to produce inosine, which pairs with cytosine during replication, enabling targeted A-to-G editing in mitochondria with high efficiency.^725^ The A-to-G editing efficiency was further enhanced by fusing the DddA6 variant with TALE-linked deoxyadenosine deaminase.^724^

However, DdCBEs may induce off-target activities in mtDNA,^712,726^ likely due to non-specific interactions between TALE and DNA or spontaneous assembly of split DddA_tox_ deaminases.^727^ To mitigate these issues, *Lee* et al. developed high-fidelity DdCBEs that are both efficient and precise, avoiding off-target mutations.^727^ Adding NES sequences also helps reduce off-target editing while improving on-target efficiency.^724^ DdCBEs offer precise mtDNA base editing both in vitro and in vivo, with the ability for germline transmission.^728^ This method is particularly valuable for creating mitochondrial disease-associated cell lines and animal models, deepening our understanding of mitochondrial disorders and providing potential avenues for correcting both homoplasmic and heteroplasmic pathogenic variants.

### Post-transcriptional modifications

Post-transcriptional modifications of mitochondrial RNA play a critical role in finely regulating the synthesis and stability of the 13 mitochondrial proteins.^280^ These modifications stabilize tRNA and introduce wobble modifications, which can ameliorate mitochondrial translation defects and present potential therapeutic avenues for mitochondrial genetic diseases.^729^ In vitro studies have demonstrated that the m.3290 T > C mutation can restore the hypomodified 5-taurinomethyluridine in mt-tRNA^Leu(UUR)^ with the m.3243 A > G mutation, thereby improving mitochondrial translation in MELAS, facilitating respiratory chain complex formation, and enhancing oxygen consumption rates.^730^ Additionally, acquiring wobble modifications in mt-tRNA^Leu(CUN)^ with the m.12300 G > A mutation can also alleviate respiratory defects associated with the m.3243 A > G mutation.^731^ Introducing wobble modifications in other isoacceptor tRNAs may also yield similar benefits.^731^

In addition, the defects in mt-tRNA modification and subsequent mitochondrial protein translation can be restored by regulating the expression of mt-tRNA-modifying enzymes. Mitochondrial translation optimization 1 (MTO1) and GTP-binding protein 3 (GTPBP3) are responsible for catalyzing the biosynthesis of 5-taurinomethyluridine.^732^ High-dose oral taurine has been shown to increase MTO1 expression,^733^ effectively preventing stroke-like episodes in MELAS by correcting the first anticodon nucleotide modification defect in mt-tRNA^Leu(UUR)^.^734^ Moreover, MTO1 overexpression restores 5-taurinomethyluridine in mutant mt-tRNA^Leu(UUR)^ in MELAS and the 2-thiouridine derivative in mutant mt-tRNA^Lys^ in MERRF, enabling efficient decoding of homologous codons independently of taurine supplementation.^729^ MTO1 may also function as an RNA chaperone, stabilizing pathogenic mt-tRNA mutations, enhancing tRNA aminoacylation efficiency, and supporting mitochondrial protein synthesis.^280,729^ Furthermore, ketogenic diet can improve OXPHOS defects independently of MTO1-mediated tRNA modifications, suggesting an alternative therapeutic approach.^735^ Overexpression of *TRMT61B* can restore the N1-methyladenosine modification at position 58 in mt-tRNA^Lys^ with the m.8344 A > G mutation in MERRF, thereby finely regulating mitochondrial protein synthesis and stability.^280^ Cysteine is essential for the 2-thiomodification of mt-tRNA,^736^ and L-cysteine has been shown to partially rescue mitochondrial translation defects in cells with m.3243 A > G and m.8344 A > G mutations.^737^ Additionally, N-acetyl-cysteine has demonstrated benefits for mitochondrial translation in cells deficient in tRNA 5-methylaminomethyl-2-thiouridylate methyltransferase (TRMU) and MTO1,^736^ indicating that restoring specific tRNA modifications could mitigate mitochondrial disease symptoms.

Further research by *Meseguer* et al. has revealed that retrograde signals from mitochondria to the nucleus, such as ROS or Ca^2+^, can increase microRNA expression in cells with various mtDNA mutations. These microRNAs, acting as post-transcriptional negative regulators, also influence mt-tRNA modifications by modulating the expression of mt-tRNA-modifying enzymes, thereby exacerbating disease phenotypes. Notably, microRNA-335/335* regulates GTPBP3 and MTO1 expression.^738^ High ROS levels induce microRNA-9/9* expression *via* the NF-κB pathway, directly targeting and reducing TRMU, GTPBP3, and MTO1 mRNA and protein levels, impairing non-mutant mt-tRNA modifications and worsening the MELAS phenotype.^264^ Consequently, microRNA antagonists may offer potential strategies to counteract these deleterious effects.^264,738^

Additional approaches to improve mitochondrial protein synthesis function include the use of the mTOR inhibitor rapamycin, which can enhance erythroid differentiation by inhibiting mTOR signaling and protein synthesis, effectively alleviating anemia symptoms in MLASA.^502,739^ Modifying the tRNA-binding domain of nuclear-encoded human mitochondrial phenylalanyl-tRNA synthetase can increase the aminoacylation efficiency of mt-tRNA^Phe^ with the G34A mutation in MERRF.^740^ Overexpressing human mitochondrial leucyl-tRNA synthetase can stabilize tRNA^Leu(UUR)^ and mitochondrial translation products, thereby rescuing respiratory chain defects in cells with the m.3243 A > G mutation.^741^ Furthermore, overexpressing mitochondrial translation elongation factors EFTu and EFG2 can improve quality control during translational elongation, partially suppressing amino acid misincorporation in complex III, complex II, and ATP6 caused by the m.3243 A > G mutation, thus ameliorating respiratory chain assembly defects.^742^

### Drug therapy

Advancements in pharmaceutical technology have led to the development of numerous drugs that are now being applied to treat mitochondrial disorders, with many having entered the clinical trial stage. These include CoQ10, idebenone, EPI-743, N-acetylcysteine, elamipretide, RT001, KH176, omaveloxolone, bezafibrate, pioglitazone, deferiprone, sodium dichloroacetate, L-arginine, interferon-gamma (IFN-γ), recombinant human erythropoietin, deoxynucleotide monophosphate and deoxynucleoside, among others. Each of these drugs operates through distinct mechanisms, targeting various aspects of mitochondrial dysfunction to potentially alleviate the symptoms or slow the progression of these disorders.

#### Antioxidants

CoQ10 plays a critical role in the mitochondrial respiratory chain,^743^ enhancing electron transport and ATP production, regulating redox signaling, stabilizing the mPTP, and preventing autophagy and apoptosis.^744^ Consequently, CoQ10 supplementation can reverse pathophysiological alterations and significantly improve clinical outcomes in conditions such as Leigh syndrome and MELAS.^263,745,746^ Furthermore, combining Idebenone with CoQ10 therapy may enhance therapeutic efficacy.^746^ CoQ10 offers tangible benefits for patients with mitochondrial diseases.

Idebenone, a CoQ10 analog, is the first drug approved in Europe for the treatment of LHON.^45^ It is reduced by cytosolic NAD(P)H oxidoreductase I (NQO1)^747^ and bypasses LHON-associated complex I dysfunction, restoring mitochondrial function by shuttling electrons directly from the cytoplasm to complex III. This mechanism maintains cellular energy production, restores ATP levels, and reduces ROS production.^747,748^ Both in vitro and in vivo studies have demonstrated Idebenone’s protective effects on RGCs and retinal integrity, preserving visual function.^749,750^ Idebenone has been shown to restore or maintain visual function in LHON, prevent color vision loss, and improve extraocular nerve dysfunction.^751,752^ Its long-term efficacy for LHON has been documented,^753^ although the therapeutic effect varies depending on the disease stage and specific pathogenic mtDNA mutation.^754^ Innovative delivery methods, such as PCL intravitreal implants loaded with Idebenone^755^ and biodegradable poly microspheres,^756^ offer controlled and prolonged intraocular administration, providing a new strategy for sustained LHON treatment. Idebenone is also used in the treatment of MELAS,^757^ Leigh syndrome,^758^ and Friedreich’s ataxia.^759^ However, Idebenone’s effect on mitochondrial respiratory chain function is dose-dependent; it can inhibit complex I activity while increasing complex II activity,^760^ potentially transforming from an antioxidant to a prooxidant and inducing mitochondrial dysfunction depending on its concentration and NQO1 expression levels.^761,762^ Due to its narrow therapeutic range, high doses of Idebenone can be cytotoxic, particularly in the ganglion cell layer.^761^ Genetic variations in NQO1 protein levels significantly influence Idebenone’s efficacy and toxicity, especially in NQO1-deficient cell lines,^747,761^ necessitating consideration of the patient’s NQO1 genotype and mtDNA mutation before treatment.^747^ Additionally, Idebenone is ineffective in correcting mitochondrial energy metabolism defects in conditions of CoQ deficiency.^763^

EPI-743, a novel p-benzoquinone therapeutic agent, enhances endogenous glutathione biosynthesis and improves oxidative status by modulating oxidoreductase enzyme activity, leading to clinical improvements in some hereditary mitochondrial disorders.^764,765^ EPI-743 has been shown to delay disease progression in Leigh syndrome^765,766^ and positively impact the recovery of visual function in LHON.^767^ Some evidence suggests that EPI-743 may be more potent than Idebenone.^768^

N-acetylcysteine, a precursor to glutathione, plays a critical role in restoring glutathione balance, improving mitochondrial complex IV dysfunction, reducing cellular oxidative damage, and ameliorating neuromuscular dysfunctions in Leigh syndrome models.^769^ When combined with cysteamine bitartrate, these benefits are further enhanced. Additionally, a combination therapy involving glucose, niacin, and N-acetylcysteine has been shown to synergistically improve respiratory chain complex I dysfunction, reduce mitochondrial stress, and boost metabolic and glutathione levels, thereby increasing resilience and preventing acute neurological and biochemical decompensation.^770^

Elamipretide, a mitochondria-targeted aromatic-cationic tetrapeptide, efficiently penetrates the OMM and rapidly localizes to the inner membrane, where it binds to cardiolipin or monolysocardiolipin.^771^ By improving the function of specific proteins involved in mitochondrial dynamics and mitophagy, Elamipretide can restore mitochondrial morphology and function,^772^ thereby enhancing skeletal muscle and cardiovascular performance in Barth syndrome and alleviating related clinical symptoms and disease progression.^773,774^ Additionally, Elamipretide may improve visual function in LHON^775^ and increase exercise capacity in patients with primary mitochondrial myopathy.^776^

RT001, a deuterated ethyl linoleate, is an orally bioavailable synthetic deuterated polyunsaturated fatty acid designed to inhibit lipid autoxidation and protect cells from oxidative stress.^777^ It has shown potential therapeutic effects in Friedreich’s ataxia, where it has been demonstrated to improve peak work capacity and oxygen consumption.^778^ However, clinical trials have yielded mixed results, with one trial indicating that RT001 may not be beneficial for treating Friedreich’s ataxia.^779^

KH176 exhibits dual antioxidant and redox-modulating properties.^780^ As a ROS-redox modulator, KH176 preserves the microstructure of the NDUFS4 mouse brain, reduces lipid peroxidation, and mitigates RGC degeneration, leading to improved rotational and gait performance in NDUFS4 mice.^781^ By targeting the thioredoxin/peroxiredoxin system, KH176 effectively lowers ROS levels and offers protection to cells with OXPHOS deficiencies.^780^ It also ameliorates neuronal network dysfunction and transcriptomic changes linked to m.3243 A > G heteroplasmy in neurons derived from human iPSCs.^782^ However, clinical studies by *Janssen* et al. suggest that KH176 may not significantly enhance clinical outcomes in patients with mitochondrial m.3243 A > G spectrum disorders.^783^

#### Metabolic modifiers

Omaveloxolone, an Nrf2 activator and NF-kB inhibitor, targets inflammatory and metabolic pathways.^784,785^ It enhances substrate availability and complex I activity, reduces endogenous lipid peroxidation and mitochondrial ROS levels, and elevates glutathione levels, thereby protecting cells from oxidative stress, maintaining Δψm, promoting mitochondrial respiration, and preventing cell death.^786^ Omaveloxolone has shown significant improvements in neurological function in Friedreich’s ataxia and can markedly slow disease progression with a favorable safety and tolerability profile.^787^ It is the first drug approved in the United States and Europe for treating Friedreich’s ataxia in patients aged 16 and older.^48^ However, given the multi-system nature of Friedreich’s ataxia, a cure may ultimately require combination therapy. Omaveloxolone may also enhance mitochondrial function and submaximal exercise tolerance, reducing heart rate and lactate production during exercise, which could benefit those with mitochondrial myopathy.^788^ Other compounds, such as (+)-Epicatechin,^789^ A0001,^790^ and Nomlacofusp,^791^ have also shown potential in treating Friedreich’s ataxia, though further research is necessary.

Bezafibrate, a PPAR activator, upregulates downstream PPAR target genes, increases mitochondrial biogenesis, and prevents cardiac dysfunction in mouse models of Barth syndrome^792,793^ and exercise intolerance.^793^ It also improves disturbances in the antioxidant system, mitochondrial quality control proteins, and mitochondrial function, offering potential treatment for Barth syndrome and dilated cardiomyopathy with ataxia syndrome.^794^ Additionally, bezafibrate enhances metabolic programming in Leigh syndrome neural progenitor cells by promoting *SURF1* gene expression and inducing PGC-1α, thereby restoring neuronal morphogenesis.^795^ It increases survival rates and mitigates disease progression in Leigh syndrome mouse models.^796^ In carriers of the m.3243 A > G mutation, bezafibrate induces mitochondrial biogenesis, improves cardiac function, and alters metabolomic profiles while increasing mitochondrial disease biomarkers in serum.^797^

Pioglitazone, a PPAR-γ agonist, increases levels of the human insulin-degrading enzyme and PITRM1 protein through PPAR-γ activation.^798^ This mechanism restores mitochondrial targeting sequence pre-processing and alleviates feedback inhibition of mitochondrial processing peptidase activity in fibroblasts from PITRM1-deficient patients, improving Frataxin maturation and mitochondrial function.^798^ In combination with deoxynucleosides, pioglitazone may increase mtDNA copy number and mitochondrial mass, reduce mtDNA-encoded transcripts, and improve mitochondrial respiration in MELAS cells with the m.3243 A > G mutation.^799^ Similarly, rosiglitazone, another PPAR-γ agonist, enhances Frataxin levels in Frataxin-deficient dorsal root ganglia neurons, boosting mitochondrial biogenesis, function, calcium homeostasis, and cell survival.^800^ Rosiglitazone also improves energy metabolism by increasing fatty acid β-oxidation in Frataxin-deficient cardiomyocytes and enhances motor function in Friedreich’s ataxia mouse models,^800^ indicating its potential as a treatment for this condition both in vitro and in vivo.^800^

Sodium dichloroacetate inhibits accelerated lactate production in mtDNA deletion mice, ameliorates chronic lactic acidosis, improves mitochondrial biogenesis, restores respiratory function, and extends lifespan.^801^ It may offer certain benefits for patients with mitochondrial diseases.^802^ Oral sodium dichloroacetate stimulates cellular energy metabolism by activating residual enzyme activity, effectively reducing blood and cerebrospinal fluid lactate levels in children with congenital lactic acidosis due to pyruvate dehydrogenase complex mutations.^803^

L-arginine, a precursor for nitric oxide synthesis, enhances nitric oxide formation in patients with impaired endothelial function.^804^ It has been shown to improve aerobic capacity and muscle metabolism in MELAS,^805^ and reverse endothelial dysfunction.^806^ L-arginine can also reduce the frequency and severity of stroke-like episodes and slow the progression of MELAS.^807^ Long-term L-arginine supplementation appears promising for MELAS therapy,^808^ though the existing evidence is of poor methodological quality, and both intravenous and oral L-arginine have shown limited clinical benefits in the acute or preventive treatment of MELAS.^809^ L-arginine may primarily serve to prevent the onset of stroke-like episodes.^810^ Citrulline, which increases de novo arginine synthesis and enhances nitric oxide production, could potentially offer better therapeutic effects than arginine.^811^ More rigorous trials are needed to fully evaluate the efficacy and safety of L-arginine and citrulline therapies.

#### Chelator

Deferiprone is known to redistribute iron and stimulate the expression of Frataxin, a mitochondrial iron chaperone.^459,812^ This action facilitates the chelation of mitochondrial labile iron, which plays a role in oxidative stress, and leads to the reactivation of iron-deficient aconitase, reduction of iron accumulation, and mitigation of iron-induced ROS synthesis and mitochondrial stress in Friedreich’s ataxia cardiomyocytes.^459,812^ As a result, deferiprone helps restore respiratory chain protein levels, salvages mitochondrial function, inhibits *TSFR* gene expression, and improves calcium handling dynamics, thereby enhancing cardiac function.^459,812^ However, the use of high doses of deferiprone can negatively impact cellular Fe-S enzyme activity and reduce Frataxin levels.^459,813^ Additionally, *Lim* et al. identified a novel lipophilic iron chelator, 2-pyridylcarboxaldehyde 2-thiophenecarboxyl hydrazone, which rapidly penetrates cells to induce iron efflux and protect Friedreich’s ataxia fibroblasts from hydrogen peroxide-induced cytotoxicity, showing potential as a treatment for iron overload.^814^

#### Others

IFN-γ has emerged as a potential treatment for Friedreich’s ataxia. Both in vitro and in vivo studies have demonstrated that IFN-γ can upregulate Frataxin levels by modulating *Frataxin* gene transcription, which helps improve sensory and motor deficits in Friedreich’s ataxia mouse models.^815^ IFN-γ enhances the expression of Nrf2 and manganese-dependent superoxide dismutase in Friedreich’s ataxia cells, activating the non-canonical Nrf2 pathway *via* p21. This activation reduces the cells’ sensitivity to hydrogen peroxide-induced cell death, thereby offering protective effects.^816^ IFN-γ treatment may also reduce cardiomyocyte damage and improve cardiac function in Friedreich’s ataxia cardiomyopathy.^817^

A single high dose of erythropoietin has been shown to sustainably elevate Frataxin levels in Friedreich’s ataxia, reduce oxidative stress markers, and improve clinical symptoms.^818^ However, this treatment can lead to an increase in hematocrit levels.^818^ In vitro studies have demonstrated that carbamylated erythropoietin, an erythropoietin derivative, can increase Frataxin levels independently of erythropoietin receptor activity and without inducing erythropoiesis.^819^ Despite these promising results, a Phase II clinical trial indicated that carbamylated erythropoietin might not have significant therapeutic effects on Friedreich’s ataxia^820^

In TK2 deficiency, deoxythymidine triphosphate levels are significantly diminished, resulting in an imbalance in the mitochondrial deoxyribonucleoside triphosphate pool.^821^ Administering oral TK2 products such as deoxycytidine and deoxythymidine monophosphates, or rapidly degradable deoxypyrimidine monophosphate products like deoxythymidine and deoxycytidine, can effectively increase deoxythymidine triphosphate concentrations in TK2-deficient mouse models.^822–824^ This treatment has been shown to restore mtDNA copy numbers and improve the activity and levels of mitochondrial respiratory chain enzymes, thereby delaying disease onset, alleviating symptoms, and extending lifespan in these models.^822–824^ Clinical studies have further demonstrated that deoxynucleotide monophosphate and deoxynucleoside therapies significantly enhance survival, swallowing, respiratory, and motor functions in patients with TK2-deficient myopathy, showcasing both efficacy and a favorable safety profile.^825,826^ However, because deoxythymidine and deoxycytidine therapy alone cannot completely halt or reverse the progression of TK2 deficiency, a combined therapeutic approach using AAV-TK2 gene therapy alongside deoxynucleosides may provide a more comprehensive and effective treatment for TK2 deficiency.^827^

### Cell therapy

Unlike skeletal muscles, satellite cells—dormant myoblasts that can be activated to re-enter the cell cycle and fuse with existing muscle fibers—rarely or never harbor mutant mtDNA.^828,829^ This unique characteristic enables muscle fiber regeneration through satellite cells, which can be stimulated by resistance training or injury-induced muscle regeneration,^828–830^ promoting hypertrophy and the incorporation of satellite cells into muscle fibers.^828–830^ This process facilitates the transfer of wild-type mtDNA from satellite cells to mature muscle fibers, altering the heteroplasmy and ultimately enhancing muscle strength and oxidative capacity.^828–830^ Although satellite cells and myoblasts are primarily suited for local muscle administration,^831^ mesoangioblasts—stem cells capable of fusing with damaged muscles—can either directly repair muscle tissue or augment the satellite cell pool to promote muscle regeneration.^832,833^ Mesoangioblasts can adhere to and traverse vascular endothelial cells, allowing for systemic arterial administration.^834^ In vitro studies have shown that the fusion of wild-type mesoangioblasts with myotubes carrying the m.3271 T > C or m.3291 T > C mutations can reduce the mtDNA mutation load and improve mitochondrial function.^831^ Moreover, mesoangioblasts from patients with mtDNA mutations or large-scale deletions show negligible corresponding mtDNA mutations or deletions and demonstrate mitochondrial function, proliferation, and myogenic differentiation abilities comparable to those of wild-type mesoangioblasts.^835^ Thus, mesoangioblasts present a viable option for autologous myogenic cell therapy, enabling the regeneration of new muscle fibers without mtDNA mutations or deletions following muscle injury.^835^ Additionally, eccentric exercise can induce skeletal muscle inflammation, prompting monocyte migration, which may enhance the efficacy of such treatments.^835,836^

In MNGIE, the infusion of healthy donor platelets or the use of hemodialysis and peritoneal dialysis can transiently restore circulating TP and temporarily reduce plasma levels of thymidine and deoxyuridine.^837,838^ However, these effects are short-lived and do not address neurological functions.^837^ HSCT offers a more definitive solution by permanently restoring TP function and thereby curing MNGIE.^839^ Both in vitro and in vivo studies have shown that HSCT can restore TP activity, correct the imbalance in the mitochondrial deoxyribonucleoside triphosphate pool in the liver, and safely halt disease progression.^840,841^ HSCT has been shown to correct the biochemical abnormalities and clinical manifestations of MNGIE,^489,842^ with a standardized protocol now in place.^843^ Recent research has also explored haploidentical transplantation as a potential treatment option.^844^ However, the timing of treatment, the preconditioning regimen, and donor selection are critical factors in determining therapeutic outcomes.^839^ HSCT is recommended for patients with the best donor match, younger age, and milder symptoms.^489^ Due to the increased risk of morbidity and mortality associated with advanced disease progression and complications, HSCT is not advised for patients with advanced MNGIE.^489,845^

### Enzyme replacement therapy

The high mortality risk associated with HSCT and the scarcity of matched donors limit the accessibility of this treatment for many patients.^844,845^ An alternative approach involves lentiviral transduction of hematopoietic stem and erythroid cell lines to produce reticulocytes containing active TP.^846^ Erythrocyte-encapsulated thymidine phosphorylase (EE-TP) can catalyze the metabolism of thymidine and deoxyuridine, which freely diffuse across the red blood cell membrane, converting them into normal products.^847^ This process reduces plasma nucleosides, improves mitochondrial dysfunction, alleviates clinical symptoms, and has shown good safety and tolerability, making EE-TP a potential enzyme replacement therapy for MNGIE.^848^ However, the therapeutic effects are temporary, as metabolite levels return to abnormal values once treatment is discontinued.^848^ Regular intravenous injections of EE-TP can help manage intracellular nucleotide imbalance in patients with MNGIE,^847^ positioning EE-TP as a viable treatment option for those without suitable HSCT donors or for patients with irreversible end-stage disease.^848^ Nonetheless, preclinical toxicity assessments have indicated that EE-TP might pose severe toxicity risks in MNGIE, necessitating careful management of immune reactions.^847^ To address these concerns, *Vocht* et al. developed active TP-encapsulating nanoreactors, which, due to their stability and lack of cytotoxicity and inflammatory response, could offer a more effective and safer enzyme replacement therapy option.^849^

### Organ transplantation therapy

Liver transplantation has emerged as a novel therapeutic option for mitochondrial genetic diseases involving the liver. The liver is a major source of TP,^850^ and in MNGIE, liver transplantation has been shown to restore TP activity, rapidly normalize nucleoside levels, and maintain them at stable low levels, leading to the improvement and stabilization of various clinical symptoms.^851,852^ While liver transplantation may not achieve complete clinical recovery, it offers a potentially safer alternative to allogeneic HSCT, especially in patients with underlying liver disease.^851^ Additionally, liver transplantation has been explored as a treatment for ethylmalonic encephalopathy.^853^ However, in an 18-month-old patient with ethylmalonic encephalopathy, liver transplantation only partially improved symptoms and did not result in a complete cure,^854^ suggesting that the procedure is most effective when performed before irreversible neurological damage occurs. In Wolcott-Rallison syndrome, which is caused by *EIF2AK3* mutations, single or combined transplantation of the liver, pancreas, and kidneys has been associated with improved overall health, although further clinical follow-up is necessary to confirm these outcomes.^855,856^ The decision to perform liver transplantation in patients with mtDNA depletion syndromes and deoxyguanosine kinase deficiency remains controversial, as post-transplant outcomes for these conditions are generally poor.^857^

### Exercise therapy

#### Aerobic training

Exercise intolerance is a prevalent symptom in patients with mitochondrial disease, directly correlating with the severity of impaired muscle OXPHOS.^858,859^ Reduced physical activity significantly contributes to decreased exercise capacity in individuals with mitochondrial myopathies.^860^ Several studies have shown that moderate-intensity aerobic training can enhance OXPHOS in skeletal muscle, promote mitochondrial proliferation, and increase the levels of respiratory chain enzymes.^861^ However, the impact of exercise on muscle mutational load remains unclear, with some studies suggesting that mutational load may increase with training,^861^ while others report no significant change in muscle mtDNA amounts or mutational load levels.^859,860^ Consequently, long-term studies are necessary to evaluate the safety and efficacy of exercise as a treatment for patients with mitochondrial myopathy. Exercise limitations in mitochondrial myopathy may also be compounded by chronic conditions such as cytochrome oxidase deficiency, in addition to primary mitochondrial dysfunction.^862^ Combining aerobic training with oral sodium dichloroacetate therapy could potentially improve aerobic capacity and motor function.^862^ Moreover, low-intensity aerobic exercise has been shown to facilitate targeted transgene delivery to specific organs, potentially enhancing the safety of gene therapy in human patients.^661^ Further research is needed to optimize exercise training regimens to maximize their benefits for patients with mitochondrial disease, ensuring both safety and therapeutic efficacy.

#### Resistance training

Endurance exercise promotes mitochondrial turnover, biogenesis, and angiogenesis by activating PGC-1α expression in skeletal muscle, thereby enhancing mitochondrial function.^863,864^ It also improves mitochondrial morphology and boosts antioxidant capacity.^864^ Training interventions that combine aerobic and resistance exercise have been shown to enhance mTOR-activated signaling pathways, PGC-1α signaling related to muscle mitochondrial biogenesis and anabolism, as well as OXPHOS complex activity and redox balance in muscle tissues.^865^ These combined improvements lead to increased aerobic fitness and muscle strength, as demonstrated in mice.^865^

### Induced pluripotent stem cells and organoid

Recent advancements in generating patient-specific human iPSCs and their derived cells and organoids have established robust models for investigating the pathophysiological mechanisms of various mitochondrial genetic diseases, including MELAS,^866^ MERRF,^283^ Leigh syndrome,^795^ Friedreich’s ataxia,^867^ and LHON^868^ These iPSCs, along with their derived cells and organoids, address the challenges of sample acquisition while preserving patient-specific genetic backgrounds and accurately replicating disease characteristics.^869^ Notably, these technologies have become powerful tools for screening potential therapeutic drugs and developing novel treatment strategies. For example, Guo et al. reprogrammed skin fibroblasts from a patient with *DGUOK*-mutated mtDNA depletion syndrome into iPSCs, which were then differentiated into hepatocyte-like cells and liver organoids.^869^ This study not only uncovered the mechanism linking iron overload to hepatocyte death but also identified N-acetylcysteine as a potential therapeutic intervention to inhibit ferroptosis in patients with mtDNA depletion syndrome.^869^ Similarly, iPSCs derived from patients with Alpers’ syndrome, as well as their differentiated neural stem cells and cortical organoids, revealed that nicotinamide riboside could ameliorate mitochondrial defects and exert neuroprotective effects.^870^ Furthermore, researchers are also utilizing iPSCs derived from patients with mitochondrial diseases to investigate the role and efficacy of gene-editing tools.^685^ Extensive research highlights the potential of iPSCs and their derived cells and organoids in modeling mitochondrial genetic diseases and exploring therapeutic strategies, offering renewed hope to patients. However, mtDNA alterations may occur during iPSC reprogramming, potentially affecting the reprogramming and differentiation processes.^871^ These changes could lead to the emergence of immunogenic neo-epitopes.^872^ Therefore, monitoring and dynamic analysis of mtDNA integrity should be integral to the quality control processes in iPSC production to ensure both safety and efficacy.^871^

Overall, interventions such as MRT, gene therapy, pharmacological treatments, and cell therapy have demonstrated significant therapeutic potential in clinical trials for mitochondrial genetic disorders. These approaches primarily function through mechanisms including the replacement of defective mtDNA/nDNA, antioxidation, metabolic regulation, and mitochondrial protection. Although the widespread clinical application of these technologies continues to face numerous challenges, efforts can be made to overcome these issues. For example, reducing the possibility of genetic drift in MRT might be achievable through gene editing technologies or the development of new biomaterials, such as the generation of in vitro oocytes.^873^ Additionally, the timing of gene therapy, tailored approaches for different mtDNA/nDNA defects, and more efficient delivery systems will further enhance the efficacy and application of gene therapy. Research on personalized therapeutic strategies and the synergistic effects of multiple drugs could mitigate the variability in drug efficacy caused by factors such as administration methods, individual differences, and varying disease stages. Numerous clinically non-translational but potentially effective therapeutic approaches remain. Mesenchymal stem cells can transfer functionally normal mitochondria to cells with hereditary mtDNA defects *via* TNT and mitoEVs, thereby increasing the proportion of normal mtDNA, improving and sustaining mitochondrial function in the long term.^874^ Further in vivo studies on the long-term effects and mechanisms of mitochondrial transfer are essential to ensure the safety and efficacy of these therapies, ultimately optimizing cell therapy for clinical application.^874^ In mitochondrial diseases, oxidative stress due to the disruption of oxidative phosphorylation leads to an imbalance between oxygen delivery and utilization, resulting in oxygen toxicity.^12^ Hypoxia can reduce the production of free radicals or abnormal signaling substrates and activate the vHL-PHD-HIF hypoxic transcriptional program, which in turn activates glycolysis to generate ATP and reduces ROS production caused by impaired electron transfer in ETC, thereby rescuing the ETC-inhibited cellular phenotype.^12^ Reducing oxygen delivery and consumption may offer therapeutic or preventive benefits for mitochondrial diseases.^12,875^ Further research is required to optimize and investigate the long-term effects of hypoxia, as well as its applicability in humans.^875^ The advent of induced pluripotent stem cells and organoids also provides powerful models for exploring the pathogenesis and therapeutic strategies of mitochondrial genetic disorders, potentially leading to the discovery of more effective treatments.

## Conclusion and Future perspectives

Since the first description of LHON in 1871,^19^ and the subsequent discoveries of the roles of mtDNA and nDNA in mitochondrial genetic disorders in 1988^32,33^ and 1995,^37^ the field has transitioned from the pre-molecular era to the molecular era. This deeper understanding of mitochondrial biology has significantly advanced our ability to elucidate pathogenesis and explore effective therapies.

Significant progress has been made in understanding the pathogenesis, improving diagnosis, and developing treatments for mitochondrial diseases. Novel mechanisms of MQC, such as migrasome-mediated exocytosis,^190^ mitolysosome exocytosis,^188^ and mitopherogenesis,^194^ have been identified, highlighting the role of a robust MQC network in enhancing mitochondrial resilience to stress and damage. Beyond OXPHOS disturbances, the broader implications of mitochondrial dysfunction and its downstream effects are receiving increasing attention. Abnormalities induced by mtDNA or nDNA mutations—such as impaired mito-nuclear communication, mitochondrial dynamics, and mitophagy—are now recognized as key factors in mitochondrial diseases.^126,227^ Notably, mutation-specific and cell-specific mito-nuclear communication has been shown to significantly influence the tissue heterogeneity observed in these disorders.^541^ Advances in NGS and other technologies have markedly improved diagnostic accuracy. Promising therapeutic strategies are also advancing, with MRT showing significant potential in treating and preventing mitochondrial genetic diseases and their germline transmission.^609^ The success of ST technology in particular is encouraging.^46^ Gene therapy, especially AAV-mediated allogeneic expression and gene replacement, is another highly promising approach, with several therapies now in clinical trials.^47^ Additional interventions such as exercise,^876^ pharmacological treatments,^48,754,826^ cell therapy,^843^ enzyme replacement therapy,^848^ and organ transplantation^877^ have demonstrated beneficial effects on mitochondrial diseases.

However, several urgent challenges remain. The genotype-phenotype correlation in mitochondrial diseases is complex and not fully understood.^4^ Despite advancements in iPSC and organoid technology, as well as gene editing, developing accurate disease models continues to be a significant challenge, limiting research progress. Existing cell (cybrid) and animal models are insufficient, particularly due to issues such as tissue specificity and heteroplasmy shift.^226^ Additionally, not all mutations can be modeled, as some homozygous mutations result in embryonic lethality.^369^ In terms of diagnosis, DNA sequencing remains the primary method for detecting mitochondrial diseases, but it faces significant limitations. Secondary mtDNA mutations caused by nDNA mutations, tissue heteroplasmy, and overlapping phenotypes complicate sequencing efforts, leading to a low overall diagnostic yield.^549,571^ This often necessitates repeated and combined testing methods, increasing the economic burden. Regarding treatment, a complete cure for mitochondrial diseases remains elusive. The application of MRT faces potential limitations due to functional incompatibilities between nuclear and mitochondrial genomes,^613^ residual donor mtDNA,^609^ and ethical concerns.^878^ Current mtDNA editing approaches are limited to correcting a few specific point mutations,^879^ and concerns about off-target effects and safety undermine confidence in gene therapy.^680,719^ Additionally, allogeneically expressed mitochondrial proteins are highly hydrophobic, and only a small portion successfully localizes to mitochondria, limiting the effectiveness of allogeneic expression therapy.^880^ Significant challenges remain in overcoming these obstacles.

Future research should increasingly focus on the relationship between genetic defects and mitochondrial dysfunction, along with the subsequent consequences. Novel MQC mechanisms offer promising avenues for investigation in mitochondrial diseases. Delving into tissue-specific and cell-specific mito-nuclear communication pathways is crucial to understanding these conditions better. Epigenetics, particularly lactylation, may play a significant role in mitochondrial diseases and warrant further exploration. A deeper understanding of mito-nuclear communication could also provide solutions to mito-nuclear incompatibility issues encountered after MRT. Given the challenges of unstable diagnostic yield and variants of uncertain significance in genome sequencing, integrating multi-omics approaches as complementary diagnostic tools could enhance diagnostic accuracy. To address the delivery limitations of gene therapy, future efforts should concentrate on developing methods to efficiently introduce nuclear-encoded products into mitochondria to ensure they function correctly. Prioritizing the development of gene therapy tools that are more precise, efficient, minimally off-target, safer, and easier to deliver is essential. Additionally, the combination of gene therapy with MRT could yield unexpected therapeutic benefits. Therapies such as drugs and exercise also require clinical evidence-based guidance for their application. Notably, combining different therapeutic approaches may offer synergistic benefits. The use of iPSCs and organoid technologies should be expanded to create models for specific mutations associated with mitochondrial genetic disorders. These models would facilitate the study of disease phenotypes and post-treatment changes, advancing our understanding of disease mechanisms and aiding in the development of new treatment strategies. Exploring the differentiation of pluripotent stem cells into oocytes for MRT could reduce the need for egg retrieval from healthy donors and minimize the waste of oocytes. The application of iPSCs and organoids as models for gene therapy also holds significant potential.

This review systematically summarizes the physiological aspects of mitochondrial metabolism, the intracellular and intercellular MQC network, and mitochondrial inflammation and apoptosis. It highlights potential molecular mechanisms, recent diagnostic advancements, and therapeutic developments in mitochondrial diseases, contributing to a deeper understanding of these conditions and guiding future research design and clinical translation of precise diagnostics and effective therapies. The future directions for research, diagnosis, and treatment outlined here aim to inspire and inform subsequent studies.
